# Biomarkers for Psychosis: Are We There Yet? Umbrella Review of 1478 Biomarkers

**DOI:** 10.1093/schizbullopen/sgae018

**Published:** 2024-08-30

**Authors:** Paola Fuentes-Claramonte, Andrés Estradé, Aleix Solanes, Valentina Ramella-Cravaro, Maria Angeles Garcia-Leon, Javier de Diego-Adeliño, Conrad Molins, Eric Fung, Marc Valentí, Gerard Anmella, Edith Pomarol-Clotet, Dominic Oliver, Eduard Vieta, Joaquim Radua, Paolo Fusar-Poli

**Affiliations:** FIDMAG Germanes Hospitalàries Research Foundation, Barcelona, Spain; Biomedical Research Networking Centre Consortium on Mental Health (CIBERSAM), Instituto de Salud Carlos III, Barcelona, Spain; Department of Psychosis Studies, Early Psychosis: Interventions and Clinical-detection (EPIC) Lab, Institute of Psychiatry Psychology and Neuroscience, King’s College London, London, UK; Institut d’Investigacions Biomèdiques August Pi i Sunyer (IDIBAPS), University of Barcelona (UB), Barcelona, Spain; Department of Psychiatry and Forensic Medicine, Barcelona Autonomous University (UAB), Barcelona, Spain; Department of Psychosis Studies, Early Psychosis: Interventions and Clinical-detection (EPIC) Lab, Institute of Psychiatry Psychology and Neuroscience, King’s College London, London, UK; FIDMAG Germanes Hospitalàries Research Foundation, Barcelona, Spain; Biomedical Research Networking Centre Consortium on Mental Health (CIBERSAM), Instituto de Salud Carlos III, Barcelona, Spain; Biomedical Research Networking Centre Consortium on Mental Health (CIBERSAM), Instituto de Salud Carlos III, Barcelona, Spain; Department of Psychiatry and Forensic Medicine, Barcelona Autonomous University (UAB), Barcelona, Spain; Sant Pau Mental Health Research Group, Institut de Recerca Sant Pau, Hospital de la Santa Creu i Sant Pau, Barcelona, Spain; Psychiatric Service, Hospital Universitari Santa Maria, Lleida, Catalonia, Spain; FIDMAG Germanes Hospitalàries Research Foundation, Barcelona, Spain; Biomedical Research Networking Centre Consortium on Mental Health (CIBERSAM), Instituto de Salud Carlos III, Barcelona, Spain; Institut d’Investigacions Biomèdiques August Pi i Sunyer (IDIBAPS), University of Barcelona (UB), Barcelona, Spain; Bipolar and Depressive Disorders Unit, Institute of Neuroscience, Hospital Clinic de Barcelona, University of Barcelona, Barcelona, Spain; Biomedical Research Networking Centre Consortium on Mental Health (CIBERSAM), Instituto de Salud Carlos III, Barcelona, Spain; Institut d’Investigacions Biomèdiques August Pi i Sunyer (IDIBAPS), University of Barcelona (UB), Barcelona, Spain; Bipolar and Depressive Disorders Unit, Institute of Neuroscience, Hospital Clinic de Barcelona, University of Barcelona, Barcelona, Spain; FIDMAG Germanes Hospitalàries Research Foundation, Barcelona, Spain; Biomedical Research Networking Centre Consortium on Mental Health (CIBERSAM), Instituto de Salud Carlos III, Barcelona, Spain; Department of Psychosis Studies, Early Psychosis: Interventions and Clinical-detection (EPIC) Lab, Institute of Psychiatry Psychology and Neuroscience, King’s College London, London, UK; Department of Psychiatry, University of Oxford, Oxford OX3 7JX, UK; NIHR Oxford Health Biomedical Research Centre, Oxford OX3 7JX, UK; OPEN Early Detection Service, Oxford Health NHS Foundation Trust, Oxford OX3 7JX, UK; Biomedical Research Networking Centre Consortium on Mental Health (CIBERSAM), Instituto de Salud Carlos III, Barcelona, Spain; Institut d’Investigacions Biomèdiques August Pi i Sunyer (IDIBAPS), University of Barcelona (UB), Barcelona, Spain; Bipolar and Depressive Disorders Unit, Institute of Neuroscience, Hospital Clinic de Barcelona, University of Barcelona, Barcelona, Spain; Biomedical Research Networking Centre Consortium on Mental Health (CIBERSAM), Instituto de Salud Carlos III, Barcelona, Spain; Department of Psychosis Studies, Early Psychosis: Interventions and Clinical-detection (EPIC) Lab, Institute of Psychiatry Psychology and Neuroscience, King’s College London, London, UK; Institut d’Investigacions Biomèdiques August Pi i Sunyer (IDIBAPS), University of Barcelona (UB), Barcelona, Spain; Department of Clinical Neuroscience, Center for Psychiatry Research, Karolinska Institutet, Stockholm, Sweden; Department of Psychosis Studies, Early Psychosis: Interventions and Clinical-detection (EPIC) Lab, Institute of Psychiatry Psychology and Neuroscience, King’s College London, London, UK; OASIS Service, South London and the Maudsley NHS Foundation Trust, London, UK; Department of Brain and Behavioral Sciences, University of Pavia, Pavia, Italy

**Keywords:** schizophrenia, psychotic disorders, peripheral biomarkers, electrophysiological biomarkers, neuroimaging biomarkers, neuropathological biomarkers

## Abstract

**Background and Hypothesis:**

This umbrella review aims to comprehensively synthesize the evidence of association between peripheral, electrophysiological, neuroimaging, neuropathological, and other biomarkers and diagnosis of psychotic disorders.

**Study Design:**

We selected systematic reviews and meta-analyses of observational studies on diagnostic biomarkers for psychotic disorders, published until February 1, 2018. Data extraction was conducted according to the Preferred Reporting Items for Systematic reviews and Meta-Analyses (PRISMA) guidelines. Evidence of association between biomarkers and psychotic disorders was classified as convincing, highly suggestive, suggestive, weak, or non-significant, using a standardized classification. Quality analyses used the Assessment of Multiple Systematic Reviews (AMSTAR) tool.

**Study Results:**

The umbrella review included 110 meta-analyses or systematic reviews corresponding to 3892 individual studies, 1478 biomarkers, and 392 210 participants. No factor showed a convincing level of evidence. Highly suggestive evidence was observed for transglutaminase autoantibodies levels (odds ratio [OR] = 7.32; 95% CI: 3.36, 15.94), mismatch negativity in auditory event-related potentials (standardized mean difference [SMD] = 0.73; 95% CI: 0.5, 0.96), P300 component latency (SMD = −0.6; 95% CI: −0.83, −0.38), ventricle-brain ratio (SMD = 0.61; 95% CI: 0.5, 0.71), and minor physical anomalies (SMD = 0.99; 95% CI: 0.64, 1.34). Suggestive evidence was observed for folate, malondialdehyde, brain-derived neurotrophic factor, homocysteine, P50 sensory gating (P50 S2/S1 ratio), frontal *N*-acetyl-aspartate, and high-frequency heart rate variability. Among the remaining biomarkers, weak evidence was found for 626 and a non-significant association for 833 factors.

**Conclusions:**

While several biomarkers present highly suggestive or suggestive evidence of association with psychotic disorders, methodological biases, and underpowered studies call for future higher-quality research.

## Introduction

Schizophrenia spectrum and other psychotic disorders have an estimated mean lifetime prevalence of 9.57 per 1000^1^ and usually begin during youth and early adulthood (meta-analytic peak age of onset at 20.5 years^2^). The initial diagnosis of psychosis usually occurs at the time of the first episode of psychosis (FEP), occurring at a late stage in the neurodevelopmental trajectory of the disorder.^3^ Following a FEP, standard care with antipsychotics is primarily symptom-focused and limited for altering the course of the disorder.^4^ Psychotic disorders continue to be associated with poor clinical outcomes (1 in 7 recovery rate^5^) and a 3-fold excess mortality rate when compared to the general population (standardized mortality rate of 3.08^6^) due to physical illness and increased suicide risk in the initial years following illness onset.^7,8^ Schizophrenia is a global leading cause of health-related disability,^9^ with associated global economic costs of up to 1.65% of the gross domestic product.^10^ Given the suboptimal results of current therapeutics, course-altering preventive interventions during the early developmental stages, such as cannabidiol^11,12^ or oxytocin,^13,14^ constitute promising future avenues for next-stage interventions.^15^ Course-altering interventions require a refined understanding of the pathophysiology and neurobiological substrates of psychosis.^3^ After decades of research, however, the causes of psychotic disorders remain elusive. The etiological models that have received the strongest empirical support suggest a complex combination of direct and interactive effects of genetic, epigenetic, and environmental factors across the developmental cycle that interfere with brain development and maturation.^16,17^

Nonetheless, the past decades have witnessed an explosion of psychosis biomarker research. Biomarkers provide clues for understanding the pathophysiological basis of psychosis,^18^ and could become key tools in the real-world implementation of precision medicine^19,20^ and individualized prediction modeling.^21,22^ For example, biomarkers can aid in developing mechanism-driven preventive interventions,^23^ identifying illness subtypes via biological screening,^24^ or in the introduction of novel diagnostic frameworks based on the pathophysiology of mental disorders.^25^ As such, biomarkers could be helpful for ascertaining the presence of a disorder or specific illness subtypes (“diagnostic” biomarkers), predicting therapeutic response (“predictive” biomarkers), and the course of the disorder (“prognostic” biomarkers), and for monitoring illness progression (“monitoring” biomarkers).^26,27^ However, the progress achieved in biomarker research has not been fully translated into real-world clinical practice,^28^ hence the current “translational gap.”^29^ Over 100 years following Bleuler’s introduction of the term “schizophrenia,”^30^ diagnosis is still based on clinical examination in accordance with DSM (Diagnostic and Statistical Manual of Mental Disorders) or ICD (International Classification of Diseases) diagnostic criteria.^31^ General psychopathological knowledge, and evidence-interventions, are implemented following a trial-and-error approach according to a general clinical profile.^23,32^

One obstacle for the translational potential of scientific findings is the fragmentation of knowledge as primary biomedical studies proliferate and diversify.^33^ This also affects secondary research due to the accumulation of overlapping, often contradictory, systematic reviews (SRs) and meta-analyses (MAs).^34,35^ Umbrella reviews help overcome this challenge by providing a synthesis and critical appraisal of SRs and MAs.^36,37^ For example, umbrella reviews for schizophrenia and psychotic disorders have focused on sociodemographic, developmental, and environmental risk factors,^38^ preventive treatments,^15^ or duration of untreated psychosis.^39^ Regarding biomarker research, umbrella reviews on schizophrenia and FEP have been limited to peripheral biomarkers, while also incorporating environmental exposures^40^ or other severe mental disorders.^41^ As such, to the best of our knowledge, no comprehensive umbrella review of biomarkers for psychosis has been published to date. Our study aims to close this gap by providing a state-of-the-art comprehensive evidence synthesis on the association between peripheral, electrophysiological, neuroimaging, neuropathological, and other biomarkers and psychotic disorders. We focus on observational studies of diagnostic biomarkers comparing individuals with psychosis vs healthy controls, as the analysis of prognostic markers would require a different design and evidence synthesis method which accounts for the time-dependency of outcomes. To increase sampling power, we use an extended sampling approach by including a diagnosis of any non-organic psychotic spectrum disorder, rather than limiting our selection to chronic and more symptomatic cases, such as schizophrenia.

In addition, biomarker research is often affected by underpowered samples, methodological biases, and inconsistent reporting practices.^26,41^ To address this challenge, we provide a hierarchical classification of the robustness of the association for each factor. To achieve this, we conduct a systematic analysis of biases through a set of a priori criteria extensively validated in previous risk factors studies for physical,^42–48^ neurological,^49–52^ and mental disorders,^38,53–56^ as well as in clinical studies.^57–60^ This classification into hierarchical levels of evidence is essential for reducing the ambiguities and contradictions often found in SRs and MAs.^61^

## Methods

We pre-registered the umbrella review protocol with the International Prospective Register of Systematic Reviews (PROSPERO; CRD42017084377).

### Search Strategy and Eligibility Criteria

Various researchers (PF-C and VR; plus JDA, EF, and CM for the neuroimaging part) systematically and independently searched *PubMed*, *Web of Science*, and *Scopus* through February 1, 2018, using the search terms (“systematic review” OR “meta-analysis”) and (“psychosis” OR “schizophrenia”), to identify SRs and MAs of studies examining potential diagnostic biomarkers for psychotic disorders. Reference lists of the SRs and MAs reaching full-text review were also carefully reviewed. Eligibility criteria included: (1) an SR or MA of individual observational studies examining associations between biological markers and psychotic disorders; (2) studies considering only established DSM or ICD diagnoses of non-organic psychotic spectrum disorders (eg, schizophrenia, schizoaffective, schizophreniform, affective psychosis [mania, depression, or bipolar disorder with psychosis], drug-induced psychosis, delusional disorder, brief psychotic disorder/acute, and transient psychotic disorder, psychosis not otherwise specified); (3) inclusion of a healthy control comparison group, and (4) studies reporting sufficient data to perform the analyses (or where data were retrievable from the authors). No language restrictions were applied.

When the biomarker dataset of an article was part of a larger dataset in another article, we only retained the latter. When two articles presented minimally overlapping datasets on the same biomarker, we used both SR or MA conjointly counting the overlapping primary studies only once. We excluded articles with an outcome other than established psychotic disorder, such as those related to relapse, remission, or treatment response of psychosis or symptom severity, and those investigating genetic markers for psychosis. We used the same inclusion/exclusion criteria for each study included in every eligible SR or MA.

### Definition of Biomarker

We used the following accepted definition of biomarker: “a characteristic that is objectively measured and evaluated as an indicator of normal biological processes or pathogenic processes.”^62^ We did not include potential genetic biomarkers because umbrella reviews of genetic variables require different analytical methods and criteria.^63^ Neither did we include potential biomarkers from whole-brain voxel-based neuroimaging studies (although we did include other types of neuroimaging data) because we would need to treat each voxel as a biomarker. Instead, we refer the reader to existing MAs of whole-brain imaging studies in psychosis.^64^

We used the definition for each biomarker provided in the corresponding SR or MA. However, for reporting purposes, we classified biomarkers into the following categories: peripheral, electrophysiologic, neuropathological, neuroimaging, and other (eg, minor physical anomalies, high-frequency heart rate variability). The clustering of different biomarkers was pragmatically operationalized following the definition presented by each individual study. These groups hold only descriptive value as the actual analyses were performed at the single marker level. There was no assumption of biological mechanisms underlying these categories, most of which are yet to be fully elucidated.

### Data Extraction and Selection

Data extraction was conducted in accordance with the Preferred Reporting Items for Systematic Reviews and Meta-analyses (PRISMA 2020) guidelines.^65^ Various investigators (PF-C and MAG-L; plus JDA, EF, CM, and AS for the neuroimaging part) conducted the following steps independently. First, we identified the potential biomarkers assessed in each selected SR or MA. Second, we confirmed that each article included in the SR or MA met our eligibility criteria for the umbrella review (ie, ICD/DSM diagnosis, healthy control group, and sufficient data for analysis). Third, we extracted the following data (from the SR or MA or, otherwise, from the individual study): (1) first author and year of publication, (2) the number of cases and controls, (3) effect size (ES) measure (standardized mean difference [SMD] for continuous biomarkers, odds ratio [OR] for binary biomarkers), and corresponding 95% confidence interval (CI), (4) means and standard deviations for cases and controls for continuous biomarkers, and the number of cases and controls with and without the biomarker for binary biomarkers. An exception was neuroimaging biomarkers, for which we only relied on the information reported in the MAs unless this information showed that the biomarker’s evidence could be stronger than class IV (see “Evidence stratification” section). An independent double extraction process was conducted for those biomarkers that presented unclear data. Fourth, for those biomarkers showing evidence of class I, II, or III, we rated the quality of the SRs or MAs that contributed studies for that biomarker using the Assessment of Multiple Systematic Reviews (AMSTAR) tool.^66^ Our quality ratings obtained a high interrater agreement (intraclass correlation = 0.924). For further information on the quality analysis, see Supplementary table 1.

### Statistical Analyses

We conducted all analyses with the package “metaumbrella” for R (https://metaumbrella.org/),^67^ that performs all the calculations necessary for stratifying evidence in umbrella reviews.^36,38,68–70^ Specifically, it calculated, for each biomarker, the ES (Hedges’ *g* for continuous variables, OR for binary variables) and its confidence and prediction intervals, the between-study heterogeneity (*I*^2^ statistic), the Egger test to detect potential publication/reporting bias, and the excess significance bias test. Hedges’ *g* values represent SMDs between the patient and control groups and are interpreted as indicative of a “small” (*g* = 0.2), “medium” (*g* = 0.5), or “large” effect (*g* = 0.80).^71^ OR provides a measure of the likelihood of presenting any specific biomarker in cases vs healthy controls, with OR >1 indicating increased likelihood and OR <1 decreasing likelihood. The *I*^2^ statistic represents the percentage of total variance resulting from heterogeneity (ie, real differences in the studies’ ES), rather than chance. Egger’s test quantifies the relationship between sample size and ES, with significant results indicating the risk of publication bias.^72^ Finally, the excess significance bias test evaluates the relative presence of studies with excessive significant findings, by comparing the observed vs the expected number of studies with significant results.^73^

### Evidence Stratification

We classified the strength of the evidence according to previous criteria^38^: class I (convincing) when the number of patients >1000, *P* < 10^−6^, *I*^2^ < 50%, the 95% prediction interval excludes the null, and no publication/reporting or excess significance biases are detected; class II (highly suggestive) when the evidence is weaker than convincing but the number of patients >1000, *P* < 10^−6^, and the largest study is statistically significant; class III (suggestive) when the evidence is weaker than highly suggestive but the number of patients >1000 and *P* < 10^−3^; and class IV (weak) when the evidence is lower than suggestive but *P* < .05.

## Results

We included 110 SRs and MAs (Supplementary figure 1) covering 1478 potential biomarkers with a cumulative sample size of 189 180 individuals with psychosis and 203 030 healthy controls. In table 1 we include the biomarkers that achieved highly suggestive (class II) or suggestive (class III) level of evidence. For the full list of biomarkers, see Supplementary tables 2–6.

**Table 1.** Biomarkers With Highly Suggestive or Suggestive Evidence of Association With Psychotic Disorders

Factor	*k*	Diagnosis	ES (95% CI)	Features Used for Classification of Level of Evidence									eOR	CE
				*N*	Cases	Controls	*P*	*I* ^2^	PI 95% CI	Egger	ESB	LS		
Peripheral biomarkers														
tTG autoantibodies^74^	1	SZ	OR, 7.32 (3.36, 15.94)	2301	1401	900	1 × 10^−6^	NA	NA	NA	No	Yes	7.32	II
Folate^75^	16	SZ	SMD, −1.44 (−2.18, −0.71)	2599	1260	1339	1.20 × 10^−4^	98%	−4.71, 1.83	No	No	Yes	0.07	III
Malondialdehyde^76–78^	26	SZ	SMD, 1.38 (0.82, 1.94)	1795	1077	718	1 × 10^−6^	94%	−1.56, 4.32	No	Yes	Yes	12.22	III
BDNF^79–82^	47	FEP, SZ	SMD, −0.69 (−1.05, −0.33)	4955	2756	2199	2.00 × 10^−4^	91%	−3.2, 1.82	No	Yes	Yes	0.29	III
Homocysteine^83^	19	SZ	SMD, 0.6 (0.31, 0.89)	3320	1303	2017	5.1 × 10^−5^	89%	−0.7, 1.91	No	Yes	Yes	2.98	III
Electrophysiologic biomarkers														
Mismatch negativity in auditory event-related potentials^84–87^	47	SZ	SMD, 0.73 (0.5, 0.96)	5649	2871	2778	<1 × 10^−6^	86%	−0.81, 2.28	No	Yes	Yes	3.77	II
P300 component latency^88,89^	56	SZ	SMD, −0.6 (−0.83, −0.38)	3502	1735	1767	<1 × 10^−6^	90%	−2.22, 1.01	No	Yes	Yes	0.33	II
P50 sensory gating (P50 S2/S1 ratio)^88,90–93^	80	AP, SZ	SMD, 0.79 (0.43, 1.16)	4999	2107	2892	2.0 × 10^−5^	93%	−2.43, 4.02	No	No	Yes	4.22	III
Neuroimaging biomarkers														
Ventricle-brain ratio^94^	72	SZ	SMD, 0.61 (0.5, 0.71)	6099	3463	2636	<1 × 10^−6^	68%	(−0.14, 1.35)	No	Yes	Yes	3.00	II
Frontal NAA^95^	68	SZ	SMD, −0.34 (−0.47, −0.2)	2868	1444	1424	1 × 10^−6^	63%	(−1.22, 0.55)	No	Yes	No	0.54	III
Other biomarkers														
Minor physical anomalies^96^	14	SZ	SMD, 0.99 (0.64, 1.34)	2160	1153	1007	<1 × 10^−6^	93%	−0.45, 2.42	No	Yes	Yes	5.99	II
High-frequency heart rate variability^97^	28	SZ	SMD, −0.99 (−1.41, −0.58)	3008	1330	1678	2 × 10^−6^	98%	−3.24, 1.25	Yes	Yes	Yes	0.16	III

*Note*: AP, affective psychosis; BDNF, brain-derived neurotrophic factor; CI, confidence interval; CE, class of evidence; eOR, equivalent odds ratio; Egger, significant Egger test; ES, effect size; ESB, excess significance bias; FEP, first episode of psychosis; *k*, number of studies for each factor; LS, largest study with significant effect; *N*, total number of participants; NA, not assessable; NAA, *N*-acetylaspartate; OR, odds ratio; PI, prediction interval; SMD, standardized mean difference; SZ, schizophrenia; tTG, transglutaminase.

### Peripheral Biomarkers

**Fig. 1.**
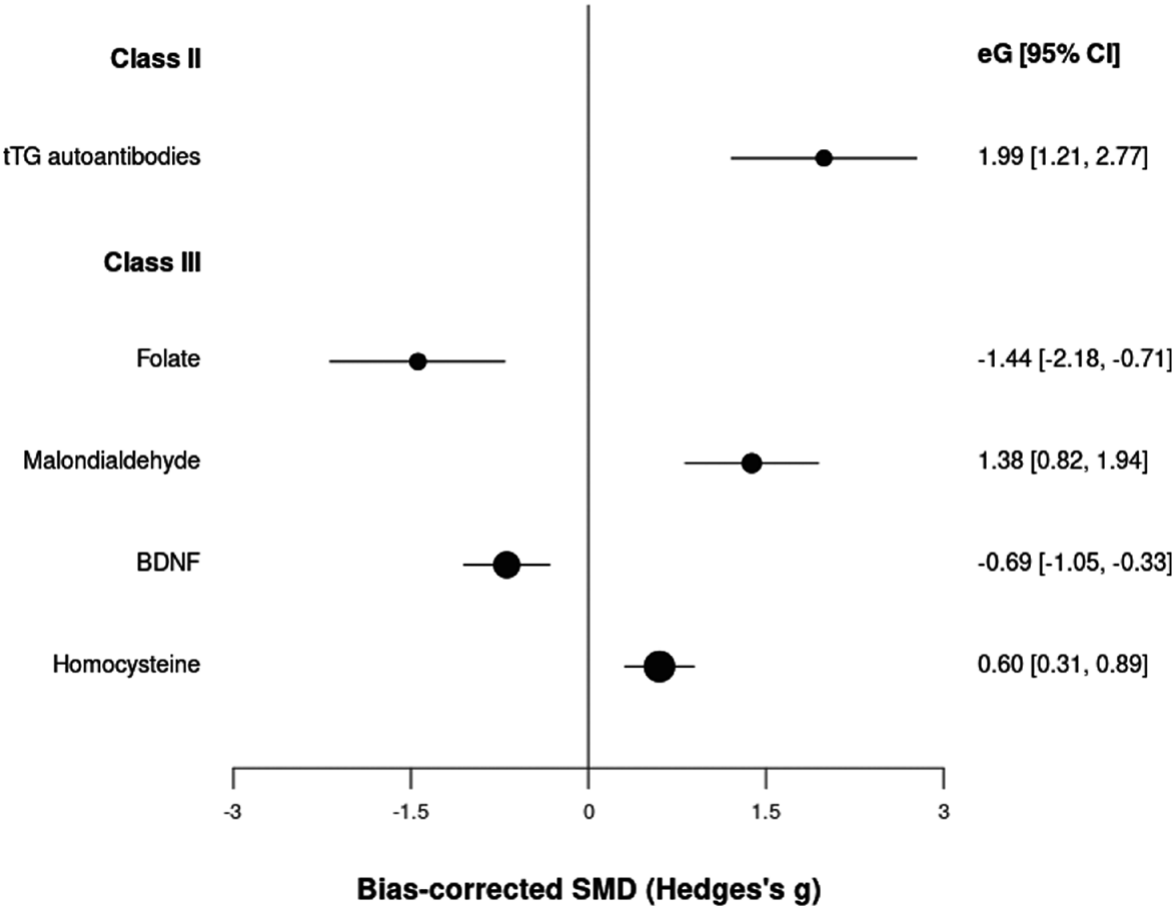
Standardized mean differences for peripheral biomarkers and psychotic disorders.

We included 284 peripheral biomarkers from 63 390 patients and 79 410 controls (cumulated sample sizes; Supplementary table 2). Transglutaminase (tTG) autoantibody levels achieved highly suggestive (class II) evidence with an OR = 7.32 (CI: 3.36, 15.94) (table 1; figure 1); we could not assess heterogeneity and potential biases because only 1 study was available for this analysis. Blood levels of folate, malondialdehyde (MDA), brain-derived neurotrophic factor (BDNF), and homocysteine (Hcy) showed class III (suggestive) evidence of association, with ESs of −1.44 (CI: −2.18, −0.71), 1.38 (CI: 0.82, 1.94), −0.69 (CI: −1.05, −0.33) and 0.60 (CI: 0. 31, 0.89), respectively. These class III biomarkers showed very large heterogeneity and (except for folate) potential excess significance bias. One hundred sixty-three other peripheral biomarkers achieved class IV evidence.

### Electrophysiologic Biomarkers

**Fig. 2.**
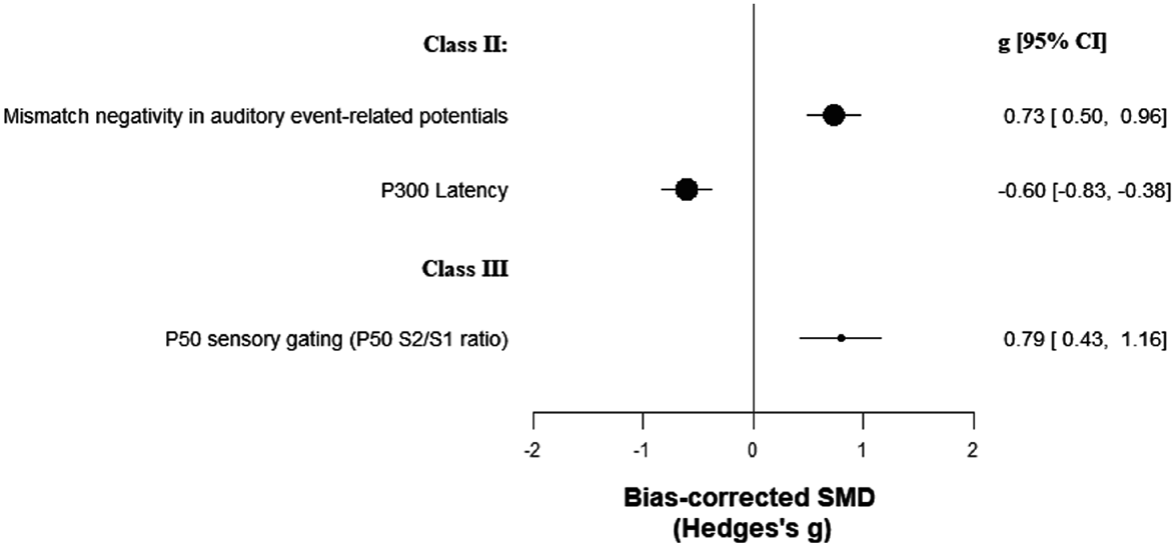
Standardized mean differences for electrophysiological biomarkers and psychotic disorders.

We examined 79 electrophysiologic biomarkers in 18 151 patients vs 19 346 controls (cumulated sample sizes; Supplementary table 3). Two biomarkers achieved class II evidence: mismatch negativity in auditory event-related potentials (ERP) (*g* = 0.73, CI: 0.50, 0.96) and P300 component latency (*g* = −0.60, CI: −0.83, −0.38) (table 1; figure 2). However, they showed very large heterogeneity and potential excess significance bias. On the other hand, P50 sensory gating achieved class III evidence with an ES of *g* = 0.79 (CI: 0.43, 1.16), but showed very large heterogeneity. Forty-one other biomarkers achieved class IV evidence.

### Neuroimaging Biomarkers

**Fig. 3.**
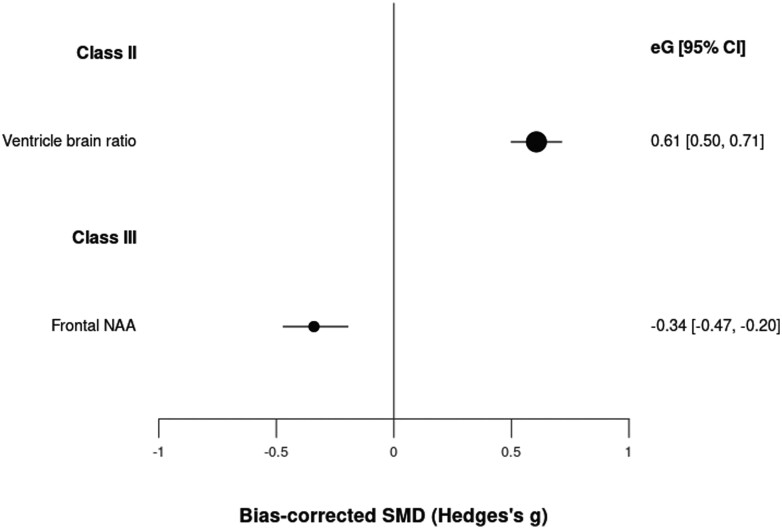
Standardized mean differences for neuroimaging biomarkers and psychotic disorders.

We included 238 neuroimaging biomarkers in a cumulative sample size of 77 849 patients vs 73 184 controls (Supplementary table 4). Two of them showed class II or III evidence: ventricle-brain ratio (class II, *g* = 0.61, CI: 0.50, 0.71) and frontal *N*-acetylaspartate (NAA) levels (class III, *g* = −0.34, CI: −0.47, −0.20) (table 1; figure 3). The analyses suggested potential excess significance bias in both cases and the heterogeneity was large (>50%). One hundred forty-nine other neuroimaging biomarkers achieved class IV evidence.

### Neuropathological Biomarkers

We studied a total of 406 neuropathological biomarkers in a cumulative sample size of 6170 patients vs 6526 controls (Supplementary table 5). One hundred thirteen biomarkers showed a statistically significant association with psychosis, all with class IV (weak) evidence.

### Other Biomarkers

**Fig. 4.**
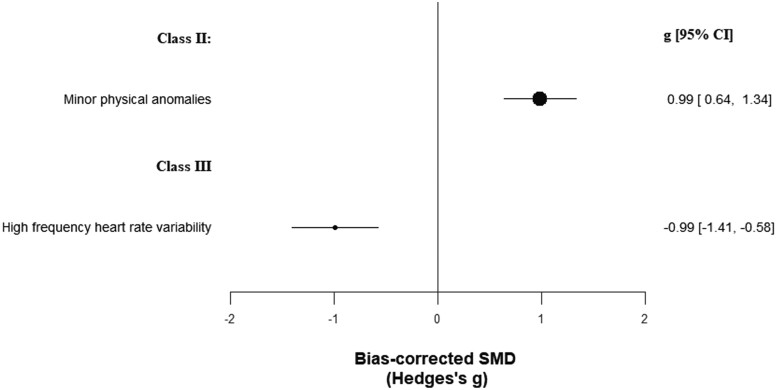
Standardized mean differences for other biomarkers and psychotic disorders.

The other biomarkers category enclosed 471 biomarkers in 23 620 patients vs 24 564 controls (cumulated sample sizes; Supplementary table 6). Minor physical anomalies achieved class II evidence (*g* = 0.99, CI: 0.64, 1.34) (table 1; figure 4), although with very large heterogeneity and potential excess significance bias. High-frequency heart rate variability achieved class III evidence (*g* = −0.99, CI: −1.41, −0.58), although, beyond very large heterogeneity, it also showed potential publication and excess significance biases. One hundred sixty other biomarkers achieved class IV evidence.

## Discussion

To the best of our knowledge, this is the first comprehensive umbrella review of diagnostic biomarkers for psychosis that incorporates peripheral, electrophysiologic, neuroimaging, neuropathological, and other biomarkers, in addition to a robust hierarchical classification of evidence. Overall, 110 SRs and MAs, a total of 3892 individual studies and 392 210 participants, and 1478 potential biomarkers were included. None of the evaluated biomarkers presented convincing evidence (class I) of association. Nonetheless, there was highly suggestive evidence (class II) for 5 biomarkers (0.3% of all evaluated factors), and suggestive evidence (class III) for 7 biomarkers (0.5%). Among the remaining biomarkers, 626 (42.6%) presented significant but weak (class IV) evidence. The remaining 833 (56.6%) presented non-significant associations.

Associations supported by highly suggestive and suggestive evidence merit discussion. Regarding peripheral biomarkers, highly suggestive evidence was only found for elevated levels of tTG autoantibodies. This finding provides evidence, for a subset of individuals, towards a link between psychosis and celiac disease.^98,99^ The relationship between celiac disease or gluten sensitivity and neurologic and psychiatric disorders was initially reported almost 70 years ago,^100,101^ with celiac disease having since been linked to an increased risk of various neurological^102^ and mental disorders.^103^ In addition, it provides evidence for psychosis as a systemic disorder with potential autoimmune components.^104,105^ While the specific nature of the association remains unknown, possible mechanisms include shared genetic susceptibility or immunological abnormalities.^106^ However, the estimate is based on a single study. As a result, heterogeneity or publication bias could not be assessed. Nonetheless, the large sample of cases (*n* = 2301) grants this association a class II level of evidence. In addition, we found suggestive evidence for reduced blood levels of folate among individuals with psychosis, although this association was characterized by very large heterogeneity. This finding further indicates potential nutrition-related mechanisms in mental disorders.^107–109^ For example, maternal exposure to nutritional deprivation during pregnancy^110^ could result in deficits in micronutrients involved in 1-carbon metabolism, particularly folate (vitamin B9) and vitamin B12, leading to increased risk due to epigenetic changes via disruptions in DNA methylation.^111–113^ Findings of decreased levels of vitamin B12 have been inconsistent^114,115^ and additional epidemiological and laboratory studies are required. We also acknowledge that there might be a bi-directional effect of psychosis on diet^116^ and therefore no causal assumption is made when interpreting diet-related biomarkers. The scarcity of prospective studies is problematic, as folate and vitamin B12 levels could be confounded by the use of antipsychotics.^117^ We also observed suggestive evidence for increased plasma levels of Hcy, further indicating a potential role of 1-carbon metabolism. Hcy is a non-protein amino acid produced in 1-carbon methyl group-transfer metabolism with various functions in brain activity,^118^ and its regulation depends on dietary folate and other B vitamins.^83^ In schizophrenia patients, plasma Hcy levels were found to correlate negatively with folate^119^ and vitamin B12,^120^ and to be associated with symptom severity^118,121,122^ and a progressive course of illness.^123^ Elevated Hcy has been observed in FEP patients,^124,125^ although not in clinical high-risk for psychosis (CHR-P) individuals.^126^ However, research in the CHR-P population is still in an emerging state. Potential mechanisms involved in the association between Hcy and psychosis include aberrant DNA methylation, altered NMDA receptor and glutamatergic transmission, toxic effects on dopaminergic neurons, premature apoptosis, oxidative stress, and placental vascular damage and fetal hypoxia.^118–120,127,128^ Nonetheless, the question remains as to whether hyperhomocysteinemia is a contributor, a consequence, or an epiphenomenon of psychosis.^120^ Adjunct folate therapy has been found to improve symptoms of depressive and bipolar disorders, but not for schizophrenia.^129^ However, emerging evidence suggests moderate effectiveness of pooled vitamin B (ie, B6, B9, B12) supplementation on total schizophrenia symptoms.^130^ It is still unclear what specific symptoms and clinical groups (eg, FEP, chronic) would benefit the most from nutritional interventions, and an opportunity for clinical stratification might exist.^4^ For example, folic acid appears to be more beneficial for the treatment of negative vs positive symptoms.^131^ Likewise, a recent randomised clinical trial (RCT) study suggested that B-vitamin supplementation could have neuroprotective effects in FEP patients with elevated Hcy.^132^ Also, it is unclear how nutritional deficiencies might interact with genetic variants.^83,133^ For example, genetic variants linked to folate-metabolism can be associated with clinical response to adjunct folate therapy.^134,135^ Overall, these findings call for further research into gene-environment interaction^17^ and potential avenues for personalized medicine.

Finally, regarding peripheral biomarkers, elevated levels of MDA and reduced levels of BDNF also presented suggestive evidence which, despite large heterogeneity, was based on large samples (*n* = 1795 and *n* = 4955, respectively). Alongside increased Hcy, Elevated MDA and reduced BDNF levels suggest a role for oxidative stress and inflammation in psychosis pathophysiology, possibly linked to immune dysregulation.^136^ Immune dysregulation in psychosis has been supported by genome-wide association^137,138^ and postmortem studies.^139^ Early-life adversity, including prenatal insults^110^ and childhood trauma,^38^ can also promote a pro-inflammatory state later in life, potentially via epigenetic changes.^140,141^ We also observed weak evidence for other oxidative stress and inflammation biomarkers, such as docosahexaenoic acid (DHA), IL-6, TNF-alpha, and total antioxidant status. Overall, these findings are consistent with evidence suggestive of antioxidant status and pro-inflammatory imbalances among FEP patients^125^ and CHR-P individuals.^142^ However, these associations are often weakened by small samples, scarcity of prospective studies, and high heterogeneity potentially resulting from between-study sample differences (eg, illness phase, developmental stage, antipsychotic status, and comorbid substance-user or other lifestyle factors).^76,125^ Heterogeneity and small ESs might further indicate that immune system involvement varies between individuals and across illness phases.^143^ For example, anti-inflammatory agents have been found to be more effective for early-phase vs chronic schizophrenia.^144^ Also, anti-inflammatory biomarkers can be associated with antipsychotic treatment response.^145^ As such, an opportunity might also exist for personalized medicine approaches^22^ via inflammatory biomarker-informed patient stratification.

In terms of electrophysiologic biomarkers, mismatch negativity (MMN) in auditory ERP achieved highly suggestive evidence. Reduced MMN is a well-replicated feature in first-episode and chronic psychosis,^28,84,85,146^ and might reflect *N*-methyl-d-aspartate (NMDA) receptor function.^147,148^ MMN does not seem to always distinguish between early and chronic schizophrenia,^149,150^ and has been suggested to stabilize following deterioration during the first 1–2 years of diagnosis.^146^ In addition, abnormal auditory MMN has also been observed to precede illness onset among CHR-P individuals,^151–153^ although not consistently,^150^ and with other studies reporting similar patterns in CHR-P and FEP individuals.^154^ Overall, there is translational potential for MMN as a biomarker for monitoring illness progression and the identification of individuals at greater risk of transition.^146,155^ In addition, we observed highly suggestive evidence for P300 component latency. Significant evidence, albeit weak, was also observed for P300 amplitude disturbances. Both P300 component latency and amplitude, a more direct biomarker of cognition than MMN,^156–158^ have been widely replicated in first-episode^159–161^ and chronic psychosis patients,^88,162^ and first-degree relatives.^163^ P300 anomalies have also been reported to respond to antipsychotic treatment in schizophrenia patients,^164^ and might be linked to gray matter volume reduction in both CHR-P^165,166^ and psychosis samples.^161^ As such, ERP deficits have the potential as biomarkers for transition risk in clinical high-risk individuals^160,167–169^ in whom both neurocognitive decline^170^ and reduction in gray matter volume^171^ precede illness onset. Finally, P50 sensory gating (P50 S2/S1 ratio) presented suggestive evidence, further supporting the existence of ERP deficits in psychosis.^28^ All class II and class III electrophysiologic biomarkers were affected by large heterogeneity.

Among neuroimaging biomarkers, we observed highly suggestive evidence of increased ventricle-brain ratio among individuals with psychosis. Since the pioneer neuroimaging studies of the 1970–1980’s,^172–175^ ventricular enlargement has remained one of the most replicated neuroanatomical correlated of schizophrenia, as reflected in the large number of participants in our summary (*n* = 6099). Ventricular enlargement has also been observed in unaffected first-degree relatives^176^ and in transitioning CHR-P samples.^177^ In addition, decreased frontal NAA achieved the suggestive level of evidence. Weak evidence for reduced NAA was also present for other brain regions (eg, temporal, cerebellum, and thalamus), further supporting an association between psychosis and loss of neuronal integrity.^3^ Deterioration in neuronal health might precede illness onset, as suggested by evidence for CHR-P individuals,^95,178^ although findings in this group have been inconsistent^179^ and better-powered prospective studies are required. Overall, neuroimaging evidence is indicative of aberrant brain trajectories that seem to precede illness onset,^180,181^ suggesting a neurodevelopmental component to the disorder,^16^ and that have potential as markers for transition risk in high-risk individuals.^182^ Following illness onset, comorbid substance use, antipsychotic medication, stress, and lifestyle factors can contribute to progressive ventricular volume increases,^183,184^ in addition to underlying neurobiological factors. As such, high between-study heterogeneity observed in neuroimaging biomarkers might reflect methodological differences affecting the selection of controls, diagnostic criteria, and clinical characteristics of included cases.^185^

For neuropathological biomarkers, none of the 406 assessed factors obtained highly suggestive or suggestive evidence. While 113 biomarkers did present significant associations, the level of evidence was weak, with most (95.6%) estimates being based on a single study, and all of them employing small samples (range of *n* = 9–176).

Regarding other biomarkers, minor physical abnormalities presented highly suggestive evidence, although very large heterogeneity and potential excess significance bias were observed. This finding is consistent with extensive evidence of early-life neurodevelopmental deviance in psychosis, including an excess of prenatal and perinatal insults,^110^ childhood motor^186^ and cognitive^187^ abnormalities, and structural brain alterations.^181,188,189^ Originally, these observations led to the neurodevelopmental hypothesis of schizophrenia,^190,191^ later expanded to incorporate environmental adversity occurring during childhood and adolescence.^16,192^ Finally, lower high-frequency heart rate variability achieved suggestive evidence. However, this association is affected by large heterogeneity potential publication, and excess significance bias. Nonetheless, this finding supports the hypothesis of reduced vagal activity among individuals with psychotic disorders as a potential endophenotype associated with executive function,^193^ emotional regulation,^194,195^ and threat inhibition.^196^ This is also consistent with the evidence of an increased risk of cardiovascular disease among individuals with schizophrenia.^197^ While it is unclear how autonomic function alterations manifest in CHR-P individuals,^198,199^ an increase in high-frequency heart rate variability has been observed in CHR-P men following intranasal oxytocin,^14^ suggesting possible novel disease-engagement psychopharmacological targets.

Overall, the evidence summarized in this study presents some limitations. First, we provide estimates of association and do not assume a cause-effect relationship between psychotic disorders and the reported biomarkers. Reverse causality^200^ is an important component in biomarker research, as the temporality of the exposure cannot always be ascertained. Furthermore, we found common methodological limitations in psychosis biomarker research. On one hand, large statistical heterogeneity (*I*^2^ > 50%) was observed in 12.1% of all 1478 factors, and in 91.7% of factors with class II or III evidence. In addition, between-study heterogeneity could not be evaluated for 74.5% of biomarkers, and for 1 factor with class II evidence. This could indicate between-study methodological differences related to data collection and reporting, or sampling criteria, which can limit the reproducibility and real-world translation of findings. High heterogeneity can also be indicative of real differences between clinical or biological subtypes, meriting further exploration.^24,201^ In addition to high heterogeneity, excess significance bias (affecting 75% of class II and III biomarkers) and the limited number of cases of <1000 (affecting 92.4% of all 1478 factors) were frequently observed. Inflated ESs have been suggested to particularly affect newly discovered associations due to suboptimal power.^202^ In our sample, 38.8% of all factors based on a single study reported statistically significant associations. Among 865 estimates based on a single study (affecting 58.5% of all factors), all but one had a sample of cases of <1000 participants. High heterogeneity, excess significance bias, and small samples are methodological limitations not restrictive of psychosis research and have also been observed in biomarker research for other mental disorders.^41,68,203,204^ Furthermore, reporting bias via the Egger test could not be assessed for 87.5% of all factors due to insufficient data or due to only one study being included, and was significant for 1.1% and non-significant for 11.4% of biomarkers. Overall, these results highlight the need for better-powered replication studies with representative samples that will allow us to assess whether these biomarkers are generalizable to other settings with different sociodemographics and service configurations. Without this understanding, the prospects of implementing these biomarkers in real-world clinical care and benefitting patients are severely limited.^205^ Moreover, implementing a biomarker that is only useful in certain (usually majority) sub-populations or settings can lead to harm and perpetuation of existing health inequities.^206,207^ Ongoing large multisite research networks are being conducted to address these limitations.^208,209^ In addition, compliance with common guidelines and practices for evaluating and reporting biomarkers^210^ is needed to increase the replicability of findings and minimize missing data. Another limitation is that the current literature search is not fully updated. This is due to the high complexity of data extraction, data quality check procedures, and synthesis that are needed for this type of analysis. However, our robust method could be leveraged by subsequent evidence syntheses in this field to mainstream updated summaries. As a final limitation of our report, we did not assess the quality of individual primary studies included in the evidence synthesis as this was outside the scope of our review. In the same lines, the scope of this review was limited to diagnostic biomarkers and we did not assess prognostic biomarkers or those useful to forecast drug response.^211^

## Conclusion

Several biomarkers presented highly suggestive or suggestive evidence of association with psychotic disorders, indicating various translational opportunities for personalized medicine. However, the widespread presence of methodological biases and underpowered studies in biomarker research highlight the need for future higher-quality research.

## Supplementary Material

sgae018_suppl_Supplementary_Material
