# Supplementary material for: Biomarkers for Psychosis: Are We There Yet? Umbrella Review of 1478 Biomarkers
Source: Schizophr Bull Open. 2024 Aug 30;5(1):sgae018. doi: 10.1093/schizbullopen/sgae018 (PMC11369642; doi:10.1093/schizbullopen/sgae018)
Supplement: sgae018_suppl_Supplementary_Material [file sgae018_suppl_supplementary_material.docx]

**SUPPLEMENTARY MATERIAL**

**sFigure 1.** PRISMA flow chart

**
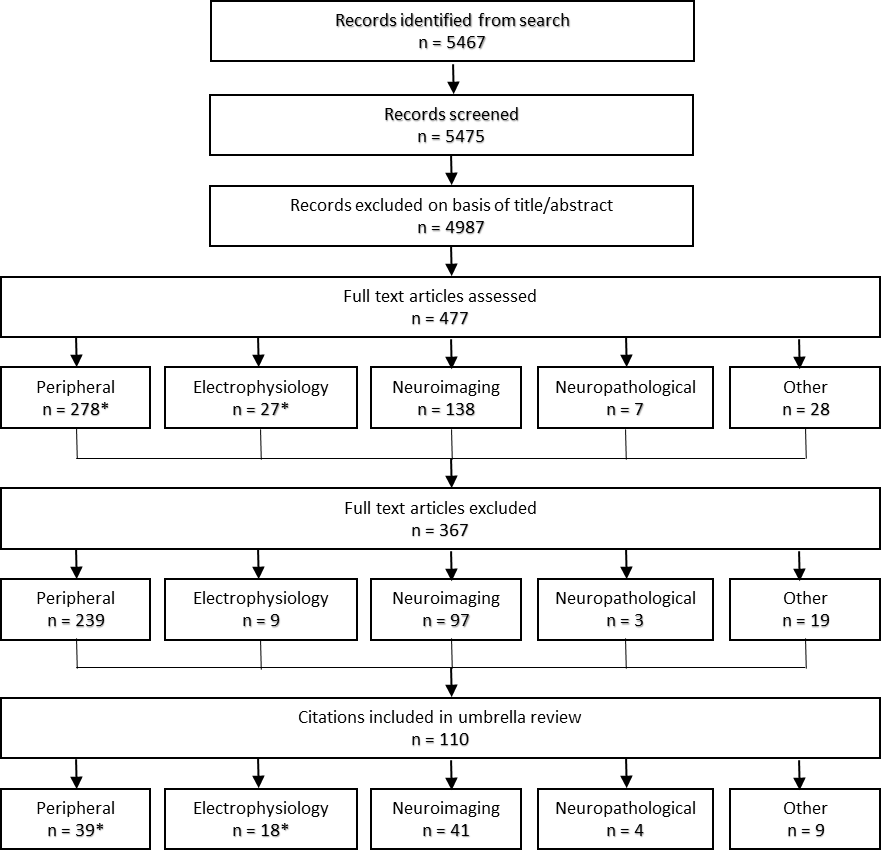
**

*The paper by Buoli 2016 is included for both peripheral and electrophysiological biomarker.

**sTable 1.** AMSTAR rating of systematic reviews and meta-analysis for class II and class III biomarkers*

| **Biomarker** | **Group** | **EC** | **Study** | **AMSTAR item** | | | | | | | | | | |
| --- | --- | --- | --- | --- | --- | --- | --- | --- | --- | --- | --- | --- | --- | --- |
|  |  |  |  | **1** | **2** | **3** | **4** | **5** | **6** | **7** | **8** | **9** | **10** | **11** |
| Transglutaminase (tTG) autoantibodies | Peripheral | II | Ezeoke 2013 | No | Yes | Yes | No | Yes | Yes | No | No | Yes | No | Yes |
| Folate | Peripheral | III | Wang 2016 | No | Yes | Yes | No | Yes | Yes | No | No | Yes | Yes | Yes |
| Malondialdehyde | Peripheral | III | Grignon 2007 | No | No | No | No | Yes | Yes | No | No | Yes | Yes | No |
| Malondialdehyde / Homocysteine | Peripheral | III / III | Davison 2017 | No | No | Yes | No | Yes | Yes | No | No | NA | No | Yes |
| Malondialdehyde | Peripheral | III | Flatow 2013 | No | Yes | Yes | No | Yes | Yes | Yes | Yes | Yes | No | Yes |
| Brain-derived neurotrophic factor (BDNF) | Peripheral | III | Cui 2012 | No | Yes | Yes | No | No | Yes | Yes | Yes | Yes | Yes | Yes |
| Brain-derived neurotrophic factor (BDNF) | Peripheral | III | Green 2011 | No | Yes | Yes | No | No | Yes | Yes | No | Yes | Yes | Yes |
| Brain-derived neurotrophic factor (BDNF) | Peripheral | III | Rowbotham 2015 | No | No | No | No | No | Yes | No | No | No | No | Yes |
| Brain-derived neurotrophic factor (BDNF) | Peripheral | III | Toll 2015 | No | No | No | No | No | Yes | No | No | No | No | Yes |
| Homocysteine | Peripheral | III | Nishi 2014 | No | Yes | No | No | No | Yes | No | No | Yes | Yes | Yes |
| Mismatch negativity in auditory event-related potentials | Electrophysiologic | II | Haigh 2017 | No | No | No | No | No | Yes | No | No | No | No | Yes |
| Mismatch negativity in auditory event-related potentials | Electrophysiologic | II | Avissar 2018 | No | No | No | No | No | Yes | No | No | No | No | Yes |
| Mismatch negativity in auditory event-related potentials | Electrophysiologic | II | Xiong 2017 | No | Yes | Yes | No | No | Yes | No | No | Yes | Yes | Yes |
| Mismatch negativity in auditory event-related potentials | Electrophysiologic | II | Umbricht 2005 | No | No | No | No | Yes | Yes | No | No | No | Yes | No |
| P300 Latency | Electrophysiologic | II | Qiu 2014 | No | Yes | Yes | No | No | Yes | No | No | Yes | Yes | Yes |
| P300 Latency / P50 sensory gating | Electrophysiologic | II / III | Bramon 2004 | No | No | Yes | No | No | Yes | No | No | Yes | Yes | No |
| P50 sensory gating | Electrophysiologic | III | Buoli 2016 | No | Yes | No | No | No | No | No | No | NA | No | Yes |
| P50 sensory gating | Electrophysiologic | III | Cheng 2016 | No | Yes | Yes | No | No | Yes | No | No | Yes | Yes | Yes |
| P50 sensory gating | Electrophysiologic | III | de Wilde 2007 | No | No | Yes | No | No | Yes | No | No | No | Yes | No |
| P50 sensory gating | Electrophysiologic | III | Patterson 2008 | No | No | No | No | No | Yes | No | No | No | No | No |
| Minor physical anomalies | Other | II | Xu 2011 | No | No | Yes | No | No | No | No | No | Yes | Yes | Yes |
| High frequency heart rate variability | Other | III | Clamor 2016 | No | No | Yes | No | No | Yes | No | No | Yes | Yes | Yes |
| Ventricle-brain ratio | Neuroimaging | II | Sayo 2012 | No | No | Yes | No | Yes | Yes | No | No | No | Yes | No |
| Frontal N-acetyl-aspartate (NAA) | Neuroimaging | III | Brugger 2011 | No | No | No | No | No | No | No | No | Yes | Yes | Yes |

* There were no class I biomarkers.

EC – evidence class, NA – not applicable. AMSTAR items: 1) Was an 'a priori' design provided?, 2) Was there duplicate study selection and data extraction?, 3) Was a comprehensive literature search performed?, 4) Was the status of publication used as an inclusion criterion?, 5) Was a list of studies (included and excluded) provided?, 6) Were the characteristics of the included studies provided?, 7) Was the scientific quality of the included studies assessed and documented?, 8) Was the scientific quality of the included studies used appropriately in formulating conclusions?, 9) Were the methods used to combine the findings of studies appropriate?, 10) Was the likelihood of publication bias assessed?, 11) Was the conflict of interest included?

**sTable 2.** Level of evidence for the association of peripheral biomarkers and psychotic disorders^1–39^

| **Factor** | **k** | **ES (95% CI)** | **Features used for classification of level of evidence** | | | | | | | | | **eOR** | **CE** |
| --- | --- | --- | --- | --- | --- | --- | --- | --- | --- | --- | --- | --- | --- |
|  |  |  | **N** | **Cases** | **Controls** | **p** | **I^2^** | **PI 95% CI** | **Egger** | **ESB** | **LS** |  |  |
| Transglutaminase (tTG) autoantibodies | 1 | OR, 7.32 (3.36, 15.94) | 2301 | 1401 | 900 | 0.000001 | NA | NA | NA | No | Yes | 7.32 | II |
| Folate | 16 | SMD, -1.44 (-2.18, -0.71) | 2599 | 1260 | 1339 | 0.00012 | 98% | -4.71, 1.83 | No | No | Yes | 0.07 | III |
| Malondialdehyde | 26 | SMD, 1.38 (0.82, 1.94) | 1795 | 1077 | 718 | 0.000001 | 94% | -1.56, 4.32 | No | Yes | Yes | 12.22 | III |
| Brain-derived neurotrophic factor (BDNF) | 47 | SMD, -0.69 (-1.05, -0.33) | 4955 | 2756 | 2199 | 0.0002 | 91% | -3.2, 1.82 | No | Yes | Yes | 0.29 | III |
| Homocystein | 19 | SMD, 0.6 (0.31, 0.89) | 3320 | 1303 | 2017 | 0.000051 | 89% | -0.7, 1.91 | No | Yes | Yes | 2.98 | III |
| 24h urine volume (ml) 24h urine | 1 | SMD, -4.94 (-7.31, -2.56) | 14 | 7 | 7 | 0.000047 | NA | NA | NA | No | Yes | <0.01 | IV |
| Cortisol awakening response (CAR) | 2 | SMD, -4.85 (-8.33, -1.38) | 125 | 68 | 57 | 0.0062 | 95% | NA | NA | No | Yes | <0.01 | IV |
| D-Lactate Serum | 1 | SMD, -5.48 (-6.7, -4.26) | 52 | 27 | 25 | <0.000001 | NA | NA | NA | No | Yes | <0.01 | IV |
| Docosapentaenoic acid (DPA) | 2 | SMD, -3.16 (-3.96, -2.36) | 68 | 52 | 16 | <0.000001 | 0% | NA | NA | No | Yes | <0.01 | IV |
| Dopamine and metabolites | 2 | SMD, -3.75 (-5.13, -2.36) | 47 | 31 | 16 | <0.000001 | 44% | NA | NA | No | Yes | <0.01 | IV |
| Excimerization of pyrene | 2 | SMD, -14.77 (-17.17, -12.38) | 83 | 38 | 45 | <0.000001 | 0% | NA | NA | No | Yes | <0.01 | IV |
| Glucose Serum | 1 | SMD, -2.93 (-3.73, -2.13) | 52 | 27 | 25 | <0.000001 | NA | NA | NA | No | Yes | <0.01 | IV |
| Glutamate Serum | 1 | SMD, -4.01 (-4.98, -3.04) | 52 | 27 | 25 | <0.000001 | NA | NA | NA | No | Yes | <0.01 | IV |
| Glycine Serum | 1 | SMD, -3.27 (-4.12, -2.42) | 52 | 27 | 25 | <0.000001 | NA | NA | NA | No | Yes | <0.01 | IV |
| High density lipoprotein (HDL) plasma | 1 | SMD, -3.57 (-4.39, -2.75) | 62 | 32 | 30 | <0.000001 | NA | NA | NA | No | Yes | <0.01 | IV |
| Hippurate Urine | 1 | SMD, -7.86 (-9.37, -6.35) | 62 | 32 | 30 | <0.000001 | NA | NA | NA | No | Yes | <0.01 | IV |
| IL18BP | 1 | SMD, -15.42 (-17.19, -13.65) | 154 | 77 | 77 | <0.000001 | NA | NA | NA | No | Yes | <0.01 | IV |
| Kynurenine (KYN) Serum | 1 | SMD, -4.21 (-5.21, -3.21) | 52 | 27 | 25 | <0.000001 | NA | NA | NA | No | Yes | <0.01 | IV |
| L-Lactate Serum | 1 | SMD, -3.32 (-4.17, -2.46) | 52 | 27 | 25 | <0.000001 | NA | NA | NA | No | Yes | <0.01 | IV |
| Lipid Plasma | 1 | SMD, -14.75 (-17.47, -12.02) | 62 | 32 | 30 | <0.000001 | NA | NA | NA | No | Yes | <0.01 | IV |
| Phosphatidylethanolamine (PEA) | 2 | SMD, -5.06 (-6.66, -3.45) | 83 | 38 | 45 | <0.000001 | 67% | NA | NA | No | Yes | <0.01 | IV |
| Tryptophan | 1 | SMD, -4.49 (-4.97, -4) | 245 | 71 | 174 | <0.000001 | NA | NA | NA | No | Yes | <0.01 | IV |
| Tryptophan Serum | 1 | SMD, -4.92 (-6.04, -3.8) | 52 | 27 | 25 | <0.000001 | NA | NA | NA | No | Yes | <0.01 | IV |
| Very low-density/low-density lipoprotein (VLDL/LDL) Plasma | 1 | SMD, -3.17 (-3.94, -2.41) | 62 | 32 | 30 | <0.000001 | NA | NA | NA | No | Yes | <0.01 | IV |
| γ-Glutamylcysteine Serum | 1 | SMD, 8.55 (6.76, 10.34) | 52 | 27 | 25 | <0.000001 | NA | NA | NA | No | Yes | >100 | IV |
| Lysophosphatidylcholine (LPC) (16:0)* Plasma (16:0)* | 1 | SMD, 8.24 (6.66, 9.82) | 62 | 32 | 30 | <0.000001 | NA | NA | NA | No | Yes | >100 | IV |
| Linoleic acid (LA) Serum | 1 | SMD, 6.97 (5.48, 8.47) | 52 | 27 | 25 | <0.000001 | NA | NA | NA | No | Yes | >100 | IV |
| Tele-methylhistamine (t-MH) CSF | 1 | SMD, 6.66 (5.02, 8.31) | 44 | 36 | 8 | <0.000001 | NA | NA | NA | No | Yes | >100 | IV |
| Conjugated dienes | 2 | SMD, 5.71 (4.7, 6.72) | 83 | 38 | 45 | <0.000001 | 0% | NA | NA | No | Yes | >100 | IV |
| Lysophosphatidylcholine (LPC) (18:2)* Plasma (18:2)* | 1 | SMD, 4.98 (3.95, 6.02) | 62 | 32 | 30 | <0.000001 | NA | NA | NA | No | Yes | >100 | IV |
| Lactate Plasma | 1 | SMD, 4.88 (3.86, 5.9) | 62 | 32 | 30 | <0.000001 | NA | NA | NA | No | Yes | >100 | IV |
| Arachidonic acid Serum | 1 | SMD, 4.66 (3.59, 5.74) | 52 | 27 | 25 | <0.000001 | NA | NA | NA | No | Yes | >100 | IV |
| Lysophosphatidylcholine (LPC) (18:1)* Plasma (18:1)* | 1 | SMD, 4.6 (3.63, 5.57) | 62 | 32 | 30 | <0.000001 | NA | NA | NA | No | Yes | >100 | IV |
| sIL-2r CSF | 1 | SMD, 4.41 (2.89, 5.92) | 26 | 16 | 10 | <0.000001 | NA | NA | NA | No | Yes | >100 | IV |
| D-serine Serum | 1 | SMD, 4.36 (3.34, 5.39) | 52 | 27 | 25 | <0.000001 | NA | NA | NA | No | Yes | >100 | IV |
| Lysophosphatidylcholine (LPC) (18:0)* Plasma (18:0)* | 1 | SMD, 4.26 (3.34, 5.18) | 62 | 32 | 30 | <0.000001 | NA | NA | NA | No | Yes | >100 | IV |
| Indoleacetic acid (IAA) 24h urine | 1 | SMD, 4.03 (1.99, 6.06) | 14 | 7 | 7 | 0.0001 | NA | NA | NA | No | Yes | >100 | IV |
| Lysophosphatidylcholine (LPC) | 2 | SMD, 3.7 (2.58, 4.83) | 83 | 38 | 45 | <0.000001 | 56% | NA | NA | No | Yes | >100 | IV |
| 3-Hydroxybutyrate (3-HB) Serum | 1 | SMD, 3.66 (2.75, 4.57) | 52 | 27 | 25 | <0.000001 | NA | NA | NA | No | Yes | >100 | IV |
| IL8 | 18 | SMD, 3.42 (0.02, 6.81) | 1157 | 639 | 518 | 0.049 | 97% | -12.54, 19.37 | No | Yes | No | >100 | IV |
| Glutathione Serum | 1 | SMD, 3.14 (2.31, 3.97) | 52 | 27 | 25 | <0.000001 | NA | NA | NA | No | Yes | >100 | IV |
| Oleic acid (OA) Serum | 1 | SMD, 2.95 (2.15, 3.76) | 52 | 27 | 25 | <0.000001 | NA | NA | NA | No | Yes | >100 | IV |
| 5-Hydroxytryptamine Serum | 1 | SMD, 2.74 (1.97, 3.51) | 52 | 27 | 25 | <0.000001 | NA | NA | NA | No | Yes | >100 | IV |
| Tyrosine Serum | 1 | SMD, 2.63 (1.87, 3.38) | 52 | 27 | 25 | <0.000001 | NA | NA | NA | No | Yes | >100 | IV |
| Palmitic acid (PA) | 2 | SMD, 2.62 (1.89, 3.35) | 68 | 52 | 16 | <0.000001 | 0% | NA | NA | No | Yes | >100 | IV |
| 3,4-dihydroxyphenylacetic (DOPAC) conjugated CSF | 1 | SMD, -2.9 (-3.89, -1.9) | 44 | 36 | 8 | <0.000001 | NA | NA | NA | No | Yes | 0.01 | IV |
| Aspartate Serum | 1 | SMD, -2.52 (-3.26, -1.78) | 52 | 27 | 25 | <0.000001 | NA | NA | NA | No | Yes | 0.01 | IV |
| Cortisol diurnal | 2 | SMD, -2.69 (-3.19, -2.2) | 125 | 68 | 57 | <0.000001 | 0% | NA | NA | No | Yes | 0.01 | IV |
| Creatinine 24h urine | 1 | SMD, -2.77 (-4.36, -1.18) | 14 | 7 | 7 | 0.00065 | NA | NA | NA | No | Yes | 0.01 | IV |
| Dopamine-sulfate CSF | 1 | SMD, -2.38 (-3.31, -1.45) | 44 | 36 | 8 | <0.000001 | NA | NA | NA | No | Yes | 0.01 | IV |
| Unsaturated fatty acids (UFA) Plasma | 1 | SMD, -2.73 (-3.43, -2.03) | 62 | 32 | 30 | <0.000001 | NA | NA | NA | No | Yes | 0.01 | IV |
| 3-indolebutyrate (IBA) fragments Plasma | 1 | SMD, 2.53 (1.85, 3.21) | 62 | 32 | 30 | <0.000001 | NA | NA | NA | No | Yes | 98.66 | IV |
| Uric Acid Urine | 1 | SMD, 2.46 (1.79, 3.12) | 62 | 32 | 30 | <0.000001 | NA | NA | NA | No | Yes | 86.08 | IV |
| Glycine Plasm | 1 | SMD, 2.38 (1.72, 3.04) | 62 | 32 | 30 | <0.000001 | NA | NA | NA | No | Yes | 75.56 | IV |
| Lysine Serum | 1 | SMD, 2.31 (1.6, 3.02) | 52 | 27 | 25 | <0.000001 | NA | NA | NA | No | Yes | 66.20 | IV |
| Tryptamine | 1 | SMD, 2.21 (1.08, 3.34) | 21 | 11 | 10 | 0.00013 | NA | NA | NA | No | Yes | 54.84 | IV |
| Biopyrrin/creatinine | 3 | SMD, 2.2 (1.34, 3.07) | 145 | 45 | 100 | 0.000001 | 74% | -7.8, 12.21 | NA | No | Yes | 54.42 | IV |
| Palmitoleic acid Serum | 1 | SMD, 2.19 (1.49, 2.89) | 52 | 27 | 25 | <0.000001 | NA | NA | NA | No | Yes | 53.21 | IV |
| Tumor necrosis factor (TNF)-α1 | 1 | SMD, 2.18 (1.84, 2.51) | 245 | 71 | 174 | <0.000001 | NA | NA | NA | No | Yes | 51.68 | IV |
| 3-methoxy-4-hydroxyphenylglycol (MHPG) 24h urine | 1 | SMD, -2.2 (-3.05, -1.35) | 36 | 20 | 16 | <0.000001 | NA | NA | NA | No | Yes | 0.02 | IV |
| Dopamine 24h urine | 1 | SMD, -2.28 (-3.14, -1.42) | 36 | 20 | 16 | <0.000001 | NA | NA | NA | No | Yes | 0.02 | IV |
| Homovanillic acid (HVA) 24h urine | 2 | SMD, -2.1 (-2.81, -1.38) | 50 | 27 | 23 | <0.000001 | 0% | NA | NA | No | Yes | 0.02 | IV |
| Phosphatidylcholine Plasma (16:0/18:2) | 1 | SMD, -2.26 (-2.9, -1.61) | 62 | 32 | 30 | <0.000001 | NA | NA | NA | No | Yes | 0.02 | IV |
| Stearic acid (SA) | 2 | SMD, 2.15 (1.47, 2.83) | 68 | 52 | 16 | <0.000001 | 0% | NA | NA | No | Yes | 49.34 | IV |
| Homovanillic acid (HVA) + Dihydroxyphenylacetic (DOPAC) (total) CSF | 1 | SMD, 2.05 (1.16, 2.93) | 44 | 36 | 8 | 0.000006 | NA | NA | NA | No | Yes | 40.86 | IV |
| Tryptophan breakdown index | 1 | SMD, 1.99 (1.67, 2.32) | 245 | 71 | 174 | <0.000001 | NA | NA | NA | No | Yes | 37.20 | IV |
| Glucose Plasma | 1 | SMD, -1.86 (-2.47, -1.26) | 62 | 32 | 30 | <0.000001 | NA | NA | NA | No | Yes | 0.03 | IV |
| Uric Acid Plasma | 1 | SMD, -1.95 (-2.56, -1.34) | 62 | 32 | 30 | <0.000001 | NA | NA | NA | No | Yes | 0.03 | IV |
| N-metil-D-aspartato (NMDA) | 2 | OR, 30.95 (4.01, 238.54) | 351 | 121 | 230 | 0.00099 | 0% | NA | NA | No | Yes | 30.95 | IV |
| α-Linolenic acid Serum | 1 | SMD, 1.89 (1.23, 2.55) | 52 | 27 | 25 | <0.000001 | NA | NA | NA | No | Yes | 30.87 | IV |
| Phenylethylamine (PEA) | 1 | SMD, 1.86 (1.38, 2.33) | 97 | 45 | 52 | <0.000001 | NA | NA | NA | No | Yes | 28.93 | IV |
| Tele-methylhistamine (t-MH) + tele-methylimidazoleacetic acid (t-MIAA) CSF | 1 | SMD, 1.84 (0.98, 2.71) | 44 | 36 | 8 | 0.00003 | NA | NA | NA | No | Yes | 28.24 | IV |
| Pregnanediol Urine | 1 | SMD, 1.82 (1.22, 2.41) | 62 | 32 | 30 | <0.000001 | NA | NA | NA | No | Yes | 26.91 | IV |
| IL4 Blood | 1 | SMD, -1.73 (-2.12, -1.34) | 212 | 38 | 174 | <0.000001 | NA | NA | NA | No | Yes | 0.04 | IV |
| Phenylalanine Serum | 1 | SMD, -1.71 (-2.35, -1.07) | 52 | 27 | 25 | <0.000001 | NA | NA | NA | No | Yes | 0.04 | IV |
| Baseline cortisol Blood | 13 | SMD, 1.71 (0.34, 3.08) | 1092 | 559 | 533 | 0.014 | 91% | -3.91, 7.34 | No | No | Yes | 22.33 | IV |
| Leucine Serum | 1 | SMD, 1.7 (1.06, 2.34) | 52 | 27 | 25 | <0.000001 | NA | NA | NA | No | Yes | 21.68 | IV |
| Threonine Serum | 1 | SMD, 1.7 (1.06, 2.34) | 52 | 27 | 25 | <0.000001 | NA | NA | NA | No | Yes | 21.66 | IV |
| Creatine Plasma | 1 | SMD, -1.66 (-2.24, -1.07) | 62 | 32 | 30 | <0.000001 | NA | NA | NA | No | Yes | 0.05 | IV |
| Total Antioxidant Status | 5 | SMD, -1.61 (-2.39, -0.83) | 387 | 193 | 194 | 0.000056 | 90% | -4.58, 1.36 | No | No | Yes | 0.05 | IV |
| IL23 | 3 | SMD, 1.62 (0.16, 3.08) | 301 | 216 | 85 | 0.029 | 96% | -16.95, 20.19 | No | No | Yes | 18.87 | IV |
| 3,4-dihydroxyphenylacetic (DOPAC) total 24h urine | 1 | SMD, -1.51 (-2.27, -0.76) | 36 | 20 | 16 | 0.000083 | NA | NA | NA | No | Yes | 0.06 | IV |
| Low-density lipoprotein (LDL) Plasma | 1 | SMD, -1.58 (-2.15, -1) | 62 | 32 | 30 | <0.000001 | NA | NA | NA | No | Yes | 0.06 | IV |
| Pros-methylimidazoleacetic acid (p-MIAA) CSF | 1 | SMD, -1.56 (-2.4, -0.72) | 44 | 36 | 8 | 0.00026 | NA | NA | NA | No | Yes | 0.06 | IV |
| Sphingomyelin | 2 | SMD, 1.52 (0.3, 2.75) | 83 | 38 | 45 | 0.015 | 83% | NA | NA | No | Yes | 15.87 | IV |
| Oleic acid (OA) | 2 | SMD, 1.51 (0.3, 2.72) | 68 | 52 | 16 | 0.015 | 73% | NA | NA | No | Yes | 15.39 | IV |
| Cortisol stress reactivity Saliva | 4 | SMD, -1.44 (-2.44, -0.44) | 80 | 38 | 42 | 0.0048 | 70% | -5.79, 2.9 | No | No | Yes | 0.07 | IV |
| Very low-density lipoprotein (VLDL) Plasma | 1 | SMD, -1.46 (-2.02, -0.89) | 62 | 32 | 30 | <0.000001 | NA | NA | NA | No | Yes | 0.07 | IV |
| 3-Hydroxybutyrate (3-HB) Plasma | 1 | SMD, -1.39 (-1.95, -0.84) | 62 | 32 | 30 | 0.000001 | NA | NA | NA | No | Yes | 0.08 | IV |
| Docosahexaenoic acid (DHA) | 6 | SMD, -1.3 (-2.32, -0.29) | 251 | 150 | 101 | 0.012 | 87% | -4.93, 2.32 | No | No | Yes | 0.09 | IV |
| Vitamin D Serum | 16 | SMD, -1.3 (-2.46, -0.15) | 7999 | 972 | 7027 | 0.027 | 93% | -6.48, 3.87 | No | Yes | No | 0.09 | IV |
| Asparagine Serum | 1 | SMD, 1.3 (0.7, 1.9) | 52 | 27 | 25 | 0.000023 | NA | NA | NA | No | Yes | 10.60 | IV |
| IL27 | 2 | SMD, 1.3 (0.66, 1.94) | 169 | 133 | 36 | 0.000074 | 62% | NA | NA | No | Yes | 10.57 | IV |
| Acetoacetate Plasma | 1 | SMD, -1.26 (-1.8, -0.71) | 62 | 32 | 30 | 0.000007 | NA | NA | NA | No | Yes | 0.10 | IV |
| Glutathione | 1 | SMD, -1.26 (-1.85, -0.67) | 54 | 29 | 25 | 0.000028 | NA | NA | NA | No | Yes | 0.10 | IV |
| Vanillylmandelic acid 24h urine | 1 | SMD, -1.28 (-2.01, -0.55) | 36 | 20 | 16 | 0.00057 | NA | NA | NA | No | Yes | 0.10 | IV |
| Phosphatidylserine | 2 | SMD, 1.26 (0.79, 1.74) | 83 | 38 | 45 | <0.000001 | 0% | NA | NA | No | Yes | 9.86 | IV |
| Total n3 fatty acids (Tn3) | 2 | SMD, -1.23 (-2.26, -0.19) | 97 | 52 | 45 | 0.02 | 81% | NA | NA | No | Yes | 0.11 | IV |
| Valine Serum | 1 | SMD, -1.22 (-1.82, -0.63) | 52 | 27 | 25 | 0.000059 | NA | NA | NA | No | Yes | 0.11 | IV |
| Vitamin D Plasma | 1 | SMD, -1.21 (-1.52, -0.9) | 186 | 93 | 93 | <0.000001 | NA | NA | NA | No | Yes | 0.11 | IV |
| IL6 | 69 | SMD, 1.2 (0.49, 1.92) | 7355 | 3388 | 3967 | 0.001 | 95% | -4.86, 7.26 | No | Yes | Yes | 8.84 | IV |
| Norepinephrine and metabolites (of creatinine) | 2 | SMD, 1.19 (0.31, 2.06) | 47 | 31 | 16 | 0.0079 | 39% | NA | NA | No | No | 8.60 | IV |
| IL3 Blood | 1 | SMD, -1.18 (-1.79, -0.58) | 50 | 24 | 26 | 0.00013 | NA | NA | NA | No | Yes | 0.12 | IV |
| Lipoprotein Plasma | 1 | SMD, -1.16 (-1.7, -0.62) | 62 | 32 | 30 | 0.000027 | NA | NA | NA | No | Yes | 0.12 | IV |
| IL1B | 3 | SMD, 1.15 (0.89, 1.41) | 282 | 141 | 141 | <0.000001 | 0% | -0.67, 2.97 | No | No | Yes | 8.08 | IV |
| Histidine Serum | 1 | SMD, 1.15 (0.56, 1.74) | 52 | 27 | 25 | 0.00014 | NA | NA | NA | No | Yes | 8.02 | IV |
| Eicosapentaenoic (EPA) | 2 | SMD, -1.06 (-1.52, -0.6) | 86 | 46 | 40 | 0.000005 | 0% | NA | NA | No | Yes | 0.15 | IV |
| High-density lipoproteins (HDL) | 2 | SMD, -1.04 (-1.47, -0.61) | 106 | 55 | 51 | 0.000002 | 0% | NA | NA | No | Yes | 0.15 | IV |
| Total n7 fatty acids (Tn7) | 2 | SMD, 1.04 (0.12, 1.96) | 97 | 52 | 45 | 0.027 | 77% | NA | NA | No | Yes | 6.58 | IV |
| Tumour Necrosis Factor alpha (TNF-α) | 47 | SMD, 1.02 (0.05, 2) | 5291 | 2413 | 2878 | 0.039 | 98% | -5.86, 7.91 | No | Yes | No | 6.41 | IV |
| Norepinephrine and metabolites | 2 | SMD, -0.97 (-1.63, -0.32) | 47 | 31 | 16 | 0.0035 | 0% | NA | NA | No | No | 0.17 | IV |
| Kynurenic acid (KYNA) Serum | 1 | SMD, 0.97 (0.39, 1.55) | 52 | 27 | 25 | 0.001 | NA | NA | NA | No | Yes | 5.79 | IV |
| Alanine Serum | 1 | SMD, -0.94 (-1.51, -0.36) | 52 | 27 | 25 | 0.0014 | NA | NA | NA | No | Yes | 0.18 | IV |
| Linoleic acid (LA) | 2 | SMD, -0.94 (-1.53, -0.35) | 68 | 52 | 16 | 0.0017 | 0% | NA | NA | No | Yes | 0.18 | IV |
| Prolactin levels | 10 | SMD, 0.93 (0.51, 1.35) | 405 | 188 | 217 | 0.000014 | 73% | -0.48, 2.35 | No | Yes | Yes | 5.44 | IV |
| Cold agglutinin | 2 | OR, 5.37 (2.16, 13.34) | 168 | 111 | 57 | 0.0003 | 0% | NA | NA | No | Yes | 5.37 | IV |
| Glutathione Reductase | 2 | SMD, -0.93 (-1.27, -0.58) | 148 | 62 | 86 | <0.000001 | 0% | NA | NA | No | Yes | 0.19 | IV |
| Norepinephrine 24h urine | 1 | SMD, -0.9 (-1.6, -0.21) | 36 | 20 | 16 | 0.011 | NA | NA | NA | No | Yes | 0.19 | IV |
| Proline Serum | 1 | SMD, -0.92 (-1.49, -0.34) | 52 | 27 | 25 | 0.0017 | NA | NA | NA | No | Yes | 0.19 | IV |
| Vitamin E | 3 | SMD, -0.83 (-1.35, -0.32) | 112 | 62 | 50 | 0.0015 | 32% | -5.63, 3.96 | No | Yes | Yes | 0.22 | IV |
| S100 calcium-binding protein B (S100B) | 20 | SMD, 0.82 (0.46, 1.18) | 1432 | 805 | 627 | 0.00001 | 89% | -0.86, 2.5 | No | Yes | Yes | 4.43 | IV |
| IL1β | 28 | SMD, 0.82 (0.15, 1.48) | 2712 | 1348 | 1364 | 0.016 | 96% | -2.88, 4.51 | No | Yes | No | 4.39 | IV |
| sIL-2r | 13 | SMD, 0.81 (0.51, 1.11) | 661 | 296 | 365 | <0.000001 | 61% | -0.18, 1.8 | No | No | Yes | 4.35 | IV |
| Isoleucine | 2 | SMD, 0.8 (0.37, 1.23) | 102 | 37 | 65 | 0.00027 | 0% | NA | NA | No | Yes | 4.25 | IV |
| Tumour Necrosis Factor alpha (TNF-α) Blood | 1 | SMD, 0.8 (0.46, 1.14) | 148 | 83 | 65 | 0.000004 | NA | NA | NA | No | Yes | 4.25 | IV |
| IL1β Blood | 2 | SMD, 0.79 (0.22, 1.36) | 188 | 98 | 90 | 0.0069 | 61% | NA | NA | No | Yes | 4.19 | IV |
| 3,4-dihydroxyphenylacetic (DOPAC) total CSF^24^ | 1 | SMD, -0.79 (-1.58, -0.01) | 44 | 36 | 8 | 0.048 | NA | NA | NA | No | Yes | 0.24 | IV |
| IL3 | 1 | SMD, 0.78 (0.34, 1.22) | 85 | 42 | 43 | 0.00052 | NA | NA | NA | No | Yes | 4.14 | IV |
| Thiobarbituric acid reactive substances | 17 | SMD, 0.77 (0.27, 1.27) | 1046 | 624 | 422 | 0.0026 | 87% | -1.44, 2.98 | No | Yes | Yes | 4.05 | IV |
| Anti-casein Immunoglobulin G (IgG) | 1 | SMD, 0.75 (0.38, 1.13) | 119 | 54 | 65 | 0.000077 | NA | NA | NA | No | Yes | 3.93 | IV |
| Soluble Tumor Necrosis Factor-α Receptor Type II (sTNF-αRII) | 1 | SMD, -0.74 (-1.25, -0.24) | 65 | 35 | 30 | 0.004 | NA | NA | NA | No | Yes | 0.26 | IV |
| Lysine | 2 | SMD, 0.73 (0.3, 1.16) | 102 | 37 | 65 | 0.00083 | 0% | NA | NA | No | Yes | 3.75 | IV |
| Albumin | 4 | SMD, -0.72 (-1.23, -0.21) | 379 | 239 | 140 | 0.0055 | 81% | -3.03, 1.58 | No | Yes | Yes | 0.27 | IV |
| Cysteine Serum | 1 | SMD, -0.7 (-1.27, -0.14) | 52 | 27 | 25 | 0.014 | NA | NA | NA | No | Yes | 0.28 | IV |
| Vitamin C | 2 | SMD, -0.69 (-1.18, -0.21) | 70 | 35 | 35 | 0.005 | 0% | NA | NA | No | Yes | 0.28 | IV |
| Kynurenic acid (KYNA) | 13 | SMD, 0.68 (0.11, 1.25) | 995 | 497 | 498 | 0.019 | 89% | -1.62, 2.98 | No | Yes | No | 3.45 | IV |
| Leucine | 2 | SMD, 0.67 (0.16, 1.18) | 102 | 37 | 65 | 0.01 | 26% | NA | NA | No | No | 3.36 | IV |
| Soluble Tumor Necrosis Factor-α Receptor Type I (sTNF-αRI) | 1 | SMD, -0.64 (-1.14, -0.14) | 65 | 35 | 30 | 0.012 | NA | NA | NA | No | Yes | 0.31 | IV |
| T helper 1:T helper 2 (Th1:Th2) ratio Serum | 5 | SMD, -0.63 (-1.25, 0) | 293 | 136 | 157 | 0.048 | 85% | -2.93, 1.68 | No | No | No | 0.32 | IV |
| IL12p70 | 1 | SMD, -0.62 (-1.15, -0.09) | 67 | 47 | 20 | 0.023 | NA | NA | NA | No | Yes | 0.33 | IV |
| Phenylalanine | 2 | SMD, 0.6 (0.18, 1.03) | 102 | 37 | 65 | 0.0052 | 0% | NA | NA | No | Yes | 2.98 | IV |
| 5-hydroxyindoleacetic acid (5-HIAA) Urine (%) | 1 | SMD, 0.58 (0.23, 0.93) | 133 | 66 | 67 | 0.001 | NA | NA | NA | No | Yes | 2.87 | IV |
| IL1Ra | 15 | SMD, 0.56 (0.21, 0.91) | 4903 | 1745 | 3158 | 0.0018 | 96% | -0.93, 2.04 | No | Yes | No | 2.74 | IV |
| Glutathione disulfide (GSSG) % | 1 | SMD, 0.55 (0.01, 1.1) | 54 | 29 | 25 | 0.047 | NA | NA | NA | No | Yes | 2.73 | IV |
| Thyroid microsomal | 1 | OR, 2.64 (1.73, 4.04) | 850 | 249 | 601 | 0.000007 | NA | NA | NA | No | Yes | 2.64 | IV |
| White blood cell count (WBC) | 2 | SMD, 0.53 (0.08, 0.99) | 84 | 48 | 36 | 0.022 | 0% | NA | NA | No | Yes | 2.63 | IV |
| Cardiolipin Immunoglobulin G (IgG) | 7 | SMD, 0.53 (0.07, 0.99) | 689 | 326 | 363 | 0.025 | 89% | -1.06, 2.12 | Yes | Yes | Yes | 2.62 | IV |
| sIL-2Rα | 1 | SMD, 0.51 (0.09, 0.93) | 90 | 45 | 45 | 0.017 | NA | NA | NA | No | Yes | 2.53 | IV |
| Baseline cortisol | 1 | SMD, -0.5 (-0.9, -0.11) | 105 | 46 | 59 | 0.012 | NA | NA | NA | No | Yes | 0.40 | IV |
| Rheumatoid factor | 4 | OR, 2.49 (1.05, 5.93) | 759 | 459 | 300 | 0.039 | 52% | 0.09, 69.48 | No | No | No | 2.49 | IV |
| C-reactive protein (CRP) | 11 | SMD, 0.5 (0.01, 0.98) | 2278 | 1077 | 1201 | 0.044 | 95% | -1.35, 2.35 | No | Yes | No | 2.47 | IV |
| IL1β CSF | 4 | SMD, -0.5 (-0.97, -0.02) | 101 | 55 | 46 | 0.041 | 25% | -1.89, 0.9 | No | No | Yes | 0.41 | IV |
| Soluble tumour necrosis factor receptor-1 (sTNFR1) | 4 | SMD, 0.49 (0.22, 0.75) | 1917 | 917 | 1000 | 0.00032 | 81% | -0.71, 1.68 | No | No | Yes | 2.41 | IV |
| Valine | 2 | SMD, 0.47 (0.05, 0.89) | 102 | 37 | 65 | 0.029 | 0% | NA | NA | No | No | 2.33 | IV |
| Histadine | 2 | SMD, -0.46 (-0.88, -0.03) | 102 | 37 | 65 | 0.035 | 2% | NA | NA | No | Yes | 0.44 | IV |
| Methionine | 2 | SMD, 0.43 (0.02, 0.85) | 102 | 37 | 65 | 0.042 | 0% | NA | NA | No | No | 2.20 | IV |
| α-1-antitrypsin | 3 | SMD, 0.43 (0.28, 0.59) | 655 | 322 | 333 | <0.000001 | 56% | -0.58, 1.44 | No | No | Yes | 2.19 | IV |
| Total saturated fatty acids (TSFA) | 2 | SMD, 0.43 (0.02, 0.84) | 97 | 52 | 45 | 0.039 | 0% | NA | NA | No | No | 2.18 | IV |
| Cardiolipin immunoglobulin M (IgM) | 8 | SMD, 0.41 (0.15, 0.67) | 908 | 510 | 398 | 0.002 | 65% | -0.37, 1.2 | No | No | Yes | 2.11 | IV |
| Tumour necrosis factor receptor-1 (TNF-R1) | 3 | SMD, 0.39 (0.18, 0.61) | 388 | 149 | 239 | 0.00037 | 0% | -1.01, 1.8 | No | No | Yes | 2.05 | IV |
| Baseline cortisol Plasma | 16 | SMD, 0.39 (0.06, 0.71) | 968 | 571 | 397 | 0.02 | 80% | -0.91, 1.69 | No | No | Yes | 2.02 | IV |
| Uric Acid | 4 | SMD, -0.32 (-0.56, -0.09) | 444 | 227 | 217 | 0.0072 | 29% | -1.11, 0.46 | No | Yes | No | 0.56 | IV |
| von Willebrand Factor (vWF) | 1 | SMD, -0.3 (-0.52, -0.08) | 362 | 121 | 241 | 0.008 | NA | NA | NA | No | Yes | 0.58 | IV |
| Vitamin D Serum | 13 | SMD, -0.28 (-0.44, -0.13) | 7254 | 592 | 6662 | 0.0004 | 67% | -0.74, 0.17 | Yes | Yes | No | 0.60 | IV |
| Amyloid P component Serum | 1 | SMD, 0.25 (0.07, 0.43) | 483 | 229 | 254 | 0.0056 | NA | NA | NA | No | Yes | 1.58 | IV |
| IL15 | 1 | SMD, 0.24 (0.06, 0.42) | 530 | 180 | 350 | 0.0085 | NA | NA | NA | No | Yes | 1.55 | IV |
| IL5 | 1 | SMD, -0.19 (-0.37, -0.01) | 530 | 180 | 350 | 0.039 | NA | NA | NA | No | No | 0.71 | IV |
| Cholesterol | 18 | SMD, -0.16 (-0.29, -0.02) | 2052 | 1012 | 1040 | 0.02 | 46% | -0.57, 0.26 | No | Yes | Yes | 0.75 | IV |
| Complement component 3 (C3) | 3 | SMD, 5.13 (-0.23, 10.49) | 570 | 290 | 280 | 0.061 | 98% | -63.66, 73.92 | No | Yes | Yes | >100 | ns |
| IL6 Blood | 2 | SMD, 1.95 (-0.49, 4.39) | 252 | 53 | 199 | 0.12 | 97% | NA | NA | No | Yes | 34.29 | ns |
| Kynurenic acid (KYNA) CSF | 2 | SMD, 1.93 (-0.73, 4.58) | 151 | 96 | 55 | 0.15 | 97% | NA | NA | Yes | Yes | 32.94 | ns |
| IL18 | 8 | SMD, 1.88 (-0.1, 3.86) | 1931 | 793 | 1138 | 0.063 | 98% | -5.52, 9.28 | No | Yes | No | 30.21 | ns |
| Total polyunsaturated fatty acids (TPUFA) | 2 | SMD, -1.83 (-4.45, 0.78) | 97 | 52 | 45 | 0.17 | 96% | NA | NA | No | No | 0.04 | ns |
| Arachidonic acid red blood cells (RBCs) | 4 | SMD, -1.63 (-3.74, 0.48) | 154 | 98 | 56 | 0.13 | 94% | -11.77, 8.52 | No | Yes | No | 0.05 | ns |
| IL12 Blood | 2 | SMD, 1.58 (-0.5, 3.66) | 190 | 77 | 113 | 0.14 | 96% | NA | NA | No | Yes | 17.62 | ns |
| Nervonic acid (NA) | 2 | SMD, -1.57 (-5.38, 2.24) | 68 | 52 | 16 | 0.42 | 97% | NA | NA | Yes | Yes | 0.06 | ns |
| Cortisol awakening response (CAR) Saliva | 2 | SMD, -1.5 (-4.15, 1.15) | 151 | 77 | 74 | 0.27 | 98% | NA | NA | Yes | Yes | 0.07 | ns |
| IL12 | 4 | SMD, 1.33 (-0.25, 2.92) | 805 | 314 | 491 | 0.1 | 98% | -6.37, 9.03 | No | Yes | No | 11.17 | ns |
| DA and met/NE and met | 2 | SMD, -1.36 (-6.3, 3.58) | 47 | 31 | 16 | 0.59 | 97% | NA | NA | No | Yes | 0.09 | ns |
| IL4 | 20 | SMD, -1.31 (-3.71, 1.1) | 1809 | 726 | 1083 | 0.29 | 98% | -13.08, 10.47 | No | Yes | Yes | 0.09 | ns |
| Benzylamine | 2 | SMD, 1.32 (-1.43, 4.07) | 122 | 76 | 46 | 0.35 | 97% | NA | NA | Yes | Yes | 10.96 | ns |
| Phosphatidylinositols | 2 | SMD, -1.22 (-3.39, 0.95) | 83 | 38 | 45 | 0.27 | 95% | NA | NA | Yes | Yes | 0.11 | ns |
| Creatinine Urine | 3 | SMD, -1.15 (-2.45, 0.15) | 257 | 130 | 127 | 0.083 | 95% | -17.64, 15.35 | No | Yes | No | 0.12 | ns |
| Arachidonic acid red blood cells (RBCs) | 2 | SMD, -1.13 (-2.92, 0.65) | 97 | 52 | 45 | 0.21 | 93% | NA | NA | No | No | 0.13 | ns |
| Phosphatidylcholine | 2 | SMD, -1.13 (-5.4, 3.13) | 83 | 38 | 45 | 0.6 | 98% | NA | NA | No | Yes | 0.13 | ns |
| Taurine | 2 | SMD, 1.09 (-0.48, 2.67) | 102 | 37 | 65 | 0.17 | 90% | NA | NA | No | No | 7.27 | ns |
| Total n6 fatty acids (Tn6) | 2 | SMD, -1.08 (-3.23, 1.07) | 97 | 52 | 45 | 0.33 | 95% | NA | NA | No | No | 0.14 | ns |
| Dopamine and metabolites (of creatinine) | 2 | SMD, -1.06 (-4.56, 2.43) | 47 | 31 | 16 | 0.55 | 95% | NA | NA | Yes | Yes | 0.15 | ns |
| Vit. D deficiency Plasma | 2 | OR, 6.18 (0.83, 45.85) | 270 | 76 | 194 | 0.075 | 82% | NA | NA | No | Yes | 6.18 | ns |
| Cluster of differentiation 56 (CD56) % | 2 | SMD, 0.99 (-0.03, 2) | 96 | 45 | 51 | 0.056 | 79% | NA | NA | No | Yes | 5.99 | ns |
| Total fatty acid (TFA) | 2 | SMD, -0.9 (-3.43, 1.64) | 97 | 52 | 45 | 0.49 | 96% | NA | NA | No | No | 0.20 | ns |
| 5-hydroxyindoleacetic acid (5-HIAA) CSF | 4 | SMD, 0.71 (-0.01, 1.42) | 213 | 127 | 86 | 0.052 | 81% | -2.49, 3.9 | No | Yes | Yes | 3.60 | ns |
| Leucocyte telomere length | 5 | SMD, -0.7 (-1.87, 0.46) | 755 | 361 | 394 | 0.23 | 98% | -5.27, 3.86 | No | Yes | No | 0.28 | ns |
| 3-methoxy-4-hydroxyphenylglycol (MHPG) CSF | 1 | SMD, -0.66 (-1.44, 0.12) | 44 | 36 | 8 | 0.097 | NA | NA | NA | No | No | 0.30 | ns |
| Double-stranded deoxyribonucleic acid (DNA-ds) | 4 | OR, 3.24 (0.63, 16.78) | 784 | 375 | 409 | 0.16 | 64% | 0, 3022.14 | No | No | No | 3.24 | ns |
| Gastric parietal cell | 1 | OR, 3.06 (0.27, 35.19) | 84 | 34 | 50 | 0.37 | NA | NA | NA | No | No | 3.06 | ns |
| Manganese superoxide dismutase | 5 | SMD, -0.62 (-1.51, 0.28) | 2325 | 1444 | 881 | 0.18 | 98% | -4.14, 2.91 | No | No | Yes | 0.33 | ns |
| 3,4-dihydroxyphenylacetic (DOPAC) free CSF | 1 | SMD, -0.58 (-1.35, 0.2) | 44 | 36 | 8 | 0.15 | NA | NA | NA | No | No | 0.35 | ns |
| Thrombopoietin (TPO) | 1 | OR, 0.37 (0.06, 2.33) | 111 | 70 | 41 | 0.29 | NA | NA | NA | No | No | 0.37 | ns |
| IL17 | 5 | SMD, 0.53 (-1.03, 2.09) | 549 | 383 | 166 | 0.51 | 98% | -5.61, 6.67 | No | Yes | Yes | 2.61 | ns |
| Smooth muscle | 2 | OR, 2.61 (0.97, 7.06) | 195 | 104 | 91 | 0.058 | 0% | NA | NA | No | No | 2.61 | ns |
| Cysteinylglycine Serum | 1 | SMD, -0.52 (-1.08, 0.03) | 52 | 27 | 25 | 0.064 | NA | NA | NA | No | No | 0.39 | ns |
| Homovanillic acid (HVA) Urine | 1 | SMD, -0.51 (-1.18, 0.16) | 36 | 20 | 16 | 0.13 | NA | NA | NA | No | No | 0.39 | ns |
| 11-deoxy-15-keto-13,14-dihydro-11ß,16ε-cyclo-PGE2 | 3 | SMD, -0.5 (-1.5, 0.5) | 92 | 46 | 46 | 0.32 | 72% | -11.77, 10.77 | No | No | No | 0.40 | ns |
| Alanine Plasma | 2 | SMD, 0.47 (-0.17, 1.11) | 102 | 37 | 65 | 0.15 | 54% | NA | NA | No | Yes | 2.33 | ns |
| Normetanephrine 24h urine | 1 | SMD, -0.46 (-1.13, 0.2) | 36 | 20 | 16 | 0.17 | NA | NA | NA | No | No | 0.43 | ns |
| Antinuclear antibodies | 8 | OR, 2.32 (0.86, 6.23) | 1347 | 732 | 615 | 0.095 | 90% | 0.08, 68.39 | No | Yes | Yes | 2.32 | ns |
| Thyroglobulin | 2 | OR, 2.26 (0.25, 20.35) | 896 | 278 | 618 | 0.47 | 85% | NA | NA | No | No | 2.26 | ns |
| Kynurenine (KYN) | 4 | SMD, 0.43 (-1.7, 2.57) | 397 | 131 | 266 | 0.69 | 99% | -9.97, 10.84 | Yes | No | Yes | 2.19 | ns |
| Apolipoprotein E (ApoE) | 1 | SMD, -0.43 (-0.99, 0.13) | 51 | 28 | 23 | 0.13 | NA | NA | NA | No | No | 0.46 | ns |
| HSP 60 | 3 | OR, 2.14 (0.66, 6.97) | 284 | 179 | 105 | 0.21 | 44% | 0, 304445.17 | No | No | Yes | 2.14 | ns |
| Leukocyte telomere length | 5 | SMD, 0.41 (-0.07, 0.89) | 1531 | 759 | 772 | 0.097 | 93% | -1.43, 2.24 | No | Yes | Yes | 2.10 | ns |
| Total n9 fatty acids (Tn9) | 2 | SMD, 0.38 (-0.02, 0.79) | 97 | 52 | 45 | 0.065 | 0% | NA | NA | No | No | 2.00 | ns |
| Th1:Th2 ratio In-vitro | 6 | SMD, 0.38 (-0.31, 1.07) | 610 | 278 | 332 | 0.28 | 93% | -2.13, 2.89 | No | No | Yes | 1.99 | ns |
| Glutamine | 2 | SMD, -0.37 (-0.97, 0.23) | 102 | 37 | 65 | 0.23 | 48% | NA | NA | No | Yes | 0.51 | ns |
| Souble-stranded deoxyribonucleic acid (DNA-ss) | 2 | OR, 1.93 (0.06, 65.95) | 409 | 167 | 242 | 0.71 | 87% | NA | NA | No | Yes | 1.93 | ns |
| Cluster of differentiation 19 (CD19) | 3 | SMD, 0.35 (-0.3, 1) | 164 | 64 | 100 | 0.29 | 72% | -7.11, 7.81 | No | Yes | No | 1.89 | ns |
| 5-hydroxyindoleacetic acid (5-HIAA) 24h urine | 2 | SMD, 0.34 (-1.66, 2.35) | 50 | 27 | 23 | 0.74 | 88% | NA | NA | No | No | 1.87 | ns |
| Smith | 2 | OR, 1.85 (0.6, 5.71) | 397 | 176 | 221 | 0.28 | 55% | NA | NA | No | Yes | 1.85 | ns |
| Transforming growth factor beta (TGF-β) | 11 | SMD, 0.34 (-0.31, 0.98) | 1179 | 473 | 706 | 0.31 | 90% | -2.18, 2.85 | No | Yes | No | 1.84 | ns |
| Gliadin immunoglobulin A (IgA) | 4 | OR, 1.81 (0.5, 6.61) | 3407 | 1992 | 1415 | 0.37 | 94% | 0, 721.93 | No | No | Yes | 1.81 | ns |
| Glutathione disulfide (GSSG) | 1 | SMD, 0.33 (-0.21, 0.87) | 54 | 29 | 25 | 0.23 | NA | NA | NA | No | No | 1.81 | ns |
| Nitric oxid metabolites (Nox) | 3 | SMD, 0.31 (-0.87, 1.5) | 210 | 105 | 105 | 0.6 | 93% | -14.65, 15.28 | No | No | Yes | 1.76 | ns |
| Nitric Oxide | 8 | SMD, -0.3 (-1.22, 0.62) | 545 | 334 | 211 | 0.52 | 94% | -3.65, 3.05 | No | Yes | Yes | 0.58 | ns |
| IL2 Blood | 2 | SMD, 0.28 (-0.97, 1.52) | 252 | 53 | 199 | 0.66 | 91% | NA | NA | No | No | 1.65 | ns |
| Cluster of differentiation 4 (CD4) | 6 | SMD, 0.27 (-0.24, 0.79) | 340 | 170 | 170 | 0.3 | 78% | -1.46, 2 | No | No | Yes | 1.64 | ns |
| Tele-methylimidazoleacetic acid (t-MIAA) CSF | 1 | SMD, -0.27 (-1.04, 0.5) | 44 | 36 | 8 | 0.49 | NA | NA | NA | No | No | 0.61 | ns |
| Cluster of differentiation 3 (CD3) | 6 | SMD, 0.27 (-0.09, 0.62) | 340 | 170 | 170 | 0.14 | 58% | -0.78, 1.32 | No | No | Yes | 1.63 | ns |
| Adiponectin | 13 | SMD, -0.26 (-0.59, 0.08) | 2444 | 860 | 1584 | 0.13 | 93% | -1.55, 1.04 | No | Yes | Yes | 0.63 | ns |
| Tumor necrosis factor receptor 2 (TNFR2) | 1 | SMD, -0.26 (-0.7, 0.19) | 81 | 35 | 46 | 0.26 | NA | NA | NA | No | No | 0.63 | ns |
| Vitamin D Plasma | 2 | SMD, 0.25 (-0.74, 1.24) | 236 | 93 | 143 | 0.62 | 92% | NA | NA | No | No | 1.57 | ns |
| Vitamin E:Cholesterol ratio | 1 | SMD, -0.24 (-0.75, 0.26) | 60 | 30 | 30 | 0.35 | NA | NA | NA | No | No | 0.64 | ns |
| Homovanillic acid (HVA) CSF | 6 | SMD, 0.23 (-0.69, 1.16) | 312 | 166 | 146 | 0.62 | 93% | -3.12, 3.59 | No | Yes | Yes | 1.53 | ns |
| Cluster of differentiation 4/Cluster of differentiation 8 (CD4/CD8) | 8 | SMD, 0.22 (-0.09, 0.53) | 415 | 218 | 197 | 0.16 | 57% | -0.68, 1.13 | No | No | Yes | 1.50 | ns |
| Baseline cortisol Serum | 12 | SMD, 0.22 (-0.11, 0.55) | 698 | 419 | 279 | 0.18 | 72% | -0.93, 1.37 | No | Yes | Yes | 1.49 | ns |
| Thyroid | 1 | OR, 1.48 (0.09, 24.58) | 84 | 34 | 50 | 0.78 | NA | NA | NA | No | No | 1.48 | ns |
| Vit. D deficiency Serum | 2 | OR, 1.48 (0.37, 5.9) | 428 | 254 | 174 | 0.58 | 83% | NA | NA | No | No | 1.48 | ns |
| homocystein Plasma | 1 | SMD, 0.2 (-0.14, 0.55) | 132 | 66 | 66 | 0.24 | NA | NA | NA | No | No | 1.45 | ns |
| IL6 CSF | 2 | SMD, 0.21 (-0.61, 1.02) | 64 | 40 | 24 | 0.62 | 55% | NA | NA | No | No | 1.45 | ns |
| Interferon (IFN) Blood | 1 | SMD, -0.2 (-0.73, 0.32) | 58 | 34 | 24 | 0.45 | NA | NA | NA | No | No | 0.69 | ns |
| 5-hydroxyindoleacetic acid (5-HIAA) CSF | 2 | SMD, -0.19 (-0.63, 0.24) | 120 | 47 | 73 | 0.38 | 24% | NA | NA | No | No | 0.70 | ns |
| Cluster of differentiation 3 (CD3) % | 8 | SMD, -0.2 (-0.58, 0.19) | 516 | 236 | 280 | 0.31 | 77% | -1.45, 1.05 | No | No | Yes | 0.70 | ns |
| Lipid peroxide | 1 | SMD, -0.2 (-0.71, 0.31) | 60 | 30 | 30 | 0.44 | NA | NA | NA | No | No | 0.70 | ns |
| IL10 | 22 | SMD, 0.19 (-0.3, 0.68) | 1780 | 872 | 908 | 0.44 | 92% | -2.2, 2.58 | No | Yes | Yes | 1.41 | ns |
| Monocyte chemoattractant protein-1 (MCP1) | 1 | SMD, 0.19 (-0.33, 0.72) | 56 | 28 | 28 | 0.48 | NA | NA | NA | No | No | 1.41 | ns |
| IL2 | 36 | SMD, -0.18 (-0.58, 0.21) | 2729 | 1252 | 1477 | 0.36 | 95% | -2.57, 2.2 | No | Yes | Yes | 0.72 | ns |
| Cluster of differentiation 56 (CD56) | 3 | SMD, 0.17 (-0.73, 1.08) | 170 | 91 | 79 | 0.71 | 86% | -11.01, 11.36 | No | No | Yes | 1.37 | ns |
| Endothelial | 1 | OR, 0.73 (0.06, 8.35) | 84 | 34 | 50 | 0.8 | NA | NA | NA | No | No | 0.73 | ns |
| Cluster of differentiation 4 (CD4) % | 8 | SMD, 0.16 (-0.09, 0.41) | 458 | 212 | 246 | 0.21 | 36% | -0.46, 0.78 | No | No | No | 1.34 | ns |
| Lymphocytes | 6 | SMD, 0.16 (-0.29, 0.61) | 259 | 123 | 136 | 0.48 | 62% | -1.23, 1.55 | No | Yes | No | 1.34 | ns |
| Superoxide dismutase | 29 | SMD, 0.15 (-0.35, 0.66) | 2095 | 1164 | 931 | 0.55 | 95% | -2.67, 2.98 | No | Yes | Yes | 1.32 | ns |
| Dopamine receptor | 1 | OR, 0.76 (0.09, 6.47) | 96 | 61 | 35 | 0.8 | NA | NA | NA | No | No | 0.76 | ns |
| α-2-macroglobulin | 2 | SMD, 0.15 (-0.96, 1.25) | 613 | 300 | 313 | 0.79 | 97% | NA | NA | No | Yes | 1.31 | ns |
| 3-methoxy-4-hydroxyphenylglycol (MHPG) CSF | 4 | SMD, 0.15 (-0.92, 1.22) | 240 | 112 | 128 | 0.78 | 93% | -4.94, 5.24 | No | No | Yes | 1.31 | ns |
| Triglyceride | 17 | SMD, 0.14 (0, 0.28) | 1825 | 923 | 902 | 0.052 | 46% | -0.31, 0.58 | No | No | No | 1.29 | ns |
| Low-density lipoprotein (LDL) | 15 | SMD, -0.13 (-0.29, 0.03) | 1787 | 861 | 926 | 0.11 | 57% | -0.67, 0.4 | Yes | Yes | Yes | 0.79 | ns |
| Glutamic acid decarboxylase 65-kilodalton isoform (GAD65) | 3 | OR, 1.25 (0.59, 2.67) | 3488 | 1480 | 2008 | 0.56 | 0% | 0.01, 168.26 | No | No | No | 1.25 | ns |
| Hippocampus Immunoglobulin G (IgG) | 2 | SMD, -0.12 (-0.48, 0.24) | 133 | 73 | 60 | 0.5 | 6% | NA | NA | No | No | 0.80 | ns |
| Glutathione Peroxidase | 24 | SMD, -0.12 (-0.58, 0.35) | 1351 | 836 | 515 | 0.63 | 90% | -2.44, 2.21 | No | Yes | Yes | 0.81 | ns |
| Isoleucine Serum | 1 | SMD, 0.1 (-0.45, 0.64) | 52 | 27 | 25 | 0.72 | NA | NA | NA | No | No | 1.20 | ns |
| IL6R | 2 | SMD, 0.1 (-0.72, 0.92) | 123 | 62 | 61 | 0.82 | 77% | NA | NA | No | No | 1.19 | ns |
| Arginine Serum | 1 | SMD, -0.08 (-0.63, 0.46) | 52 | 27 | 25 | 0.76 | NA | NA | NA | No | No | 0.86 | ns |
| Cluster of differentiation 40 (sCD40) ligand | 1 | SMD, 0.08 (-0.14, 0.3) | 362 | 121 | 241 | 0.49 | NA | NA | NA | No | No | 1.15 | ns |
| IL12p40 | 1 | SMD, -0.08 (-0.25, 0.1) | 530 | 180 | 350 | 0.41 | NA | NA | NA | No | No | 0.87 | ns |
| Keratin | 1 | OR, 0.87 (0.14, 5.46) | 111 | 70 | 41 | 0.88 | NA | NA | NA | No | No | 0.87 | ns |
| L-serine Serum | 1 | SMD, 0.07 (-0.47, 0.62) | 52 | 27 | 25 | 0.8 | NA | NA | NA | No | No | 1.14 | ns |
| Norepinephrine CSF | 2 | SMD, -0.07 (-0.51, 0.37) | 99 | 58 | 41 | 0.76 | 0% | NA | NA | No | No | 0.88 | ns |
| Vitamin A | 1 | SMD, -0.07 (-0.58, 0.44) | 60 | 30 | 30 | 0.78 | NA | NA | NA | No | No | 0.88 | ns |
| Sarcolemma | 1 | OR, 1.11 (0.23, 5.32) | 84 | 34 | 50 | 0.89 | NA | NA | NA | No | No | 1.11 | ns |
| Serum soluble Tumor Necrosis Factor Receptor 2 (sTNFR2) | 1 | SMD, 0.06 (-0.38, 0.5) | 81 | 46 | 35 | 0.79 | NA | NA | NA | No | No | 1.11 | ns |
| Baseline cortisol Saliva | 8 | SMD, 0.05 (-0.92, 1.02) | 486 | 277 | 209 | 0.92 | 94% | -3.49, 3.59 | Yes | Yes | No | 1.10 | ns |
| Cluster of differentiation 19 (CD19) % | 2 | SMD, 0.05 (-0.75, 0.85) | 122 | 71 | 51 | 0.9 | 79% | NA | NA | No | No | 1.10 | ns |
| Mitochondria | 1 | OR, 0.91 (0.24, 3.39) | 111 | 70 | 41 | 0.88 | NA | NA | NA | No | No | 0.91 | ns |
| Red cell acetylcholine esterase (U/gb Hb) | 1 | SMD, 0.04 (-0.31, 0.38) | 128 | 61 | 67 | 0.84 | NA | NA | NA | No | No | 1.07 | ns |
| Cluster of differentiation 8 (CD8) | 6 | SMD, -0.03 (-0.28, 0.21) | 340 | 170 | 170 | 0.79 | 37% | -0.52, 0.45 | No | No | No | 0.94 | ns |
| Glutamine Serum | 1 | SMD, 0.03 (-0.51, 0.58) | 52 | 27 | 25 | 0.91 | NA | NA | NA | No | No | 1.06 | ns |
| Phospholipid | 2 | OR, 0.95 (0.46, 1.97) | 956 | 140 | 816 | 0.89 | 0% | NA | NA | No | No | 0.95 | ns |
| Interleukin-2 receptor alpha chain (CD25) % | 2 | SMD, 0.03 (-0.69, 0.74) | 96 | 45 | 51 | 0.94 | 65% | NA | NA | No | No | 1.05 | ns |
| Interferon‐gamma (IFN-γ) | 39 | SMD, -0.02 (-0.51, 0.46) | 3682 | 1657 | 2025 | 0.93 | 96% | -3.13, 3.08 | No | Yes | No | 0.96 | ns |
| Ganglioside | 2 | OR, 0.97 (0.47, 2) | 158 | 85 | 73 | 0.93 | 0% | NA | NA | No | No | 0.97 | ns |
| Nerve growth factor (NGF) | 15 | SMD, -0.01 (-0.39, 0.36) | 1802 | 942 | 860 | 0.94 | 89% | -1.59, 1.56 | No | Yes | Yes | 0.97 | ns |
| Catalase | 17 | SMD, 0.01 (-0.59, 0.61) | 1133 | 653 | 480 | 0.97 | 93% | -2.67, 2.69 | No | Yes | No | 1.02 | ns |
| Lupus anticoagulant | 3 | OR, 0.99 (0.67, 1.46) | 343 | 268 | 75 | 0.94 | 0% | 0.08, 12.36 | No | No | No | 0.99 | ns |
| IL2 CSF | 4 | SMD, 0 (-0.37, 0.37) | 157 | 100 | 57 | 1.0 | 7% | -1.04, 1.04 | No | No | No | 1.00 | ns |

CI – confidence interval, CE – class of evidence, CSF - cerebrospinal fluid, eOR – equivalent odds ratio, Egger – significant Egger test, ES – effect size, ESB – excess significance bias, IL – interleukin, k – number of studies for each factor, LS - largest study with significant effect, N – total number of participants, NA – not assessable, ns – not significant, OR – odds ratio, PI – prediction interval, sIL – soluble interleukin, SMD – standardized mean difference.

**sTable 3.** Level of evidence for the association of electrophysiologic biomarkers and psychotic disorders^31,40–56^

| **Factor** | **k** | **ES (95% CI)** | **Features used for classification of level of evidence** | | | | | | | | | **eOR** | **CE** |
| --- | --- | --- | --- | --- | --- | --- | --- | --- | --- | --- | --- | --- | --- |
|  |  |  | **N** | **Cases** | **Controls** | **p** | **I^2^** | **PI 95% CI** | **Egger** | **ESB** | **LS** |  |  |
| Mismatch negativity in auditory event-related potentials | 47 | SMD, 0.73 (0.5, 0.96) | 5649 | 2871 | 2778 | <0.000001 | 86% | -0.81, 2.28 | No | Yes | Yes | 3.77 | II |
| P300 component latency | 56 | SMD, -0.6 (-0.83, -0.38) | 3502 | 1735 | 1767 | <0.000001 | 90% | -2.22, 1.01 | No | Yes | Yes | 0.33 | II |
| P50 sensory gating (P50 S2/S1 ratio) | 80 | SMD, 0.79 (0.43, 1.16) | 4999 | 2107 | 2892 | 0.00002 | 93% | -2.43, 4.02 | No | No | Yes | 4.22 | III |
| N400 peak latency (unrelated condition) | 1 | SMD, 1.71 (0.93, 2.48) | 36 | 19 | 17 | 0.000016 | NA | NA | NA | No | Yes | 22.11 | IV |
| N400 peak latency (related condition) | 1 | SMD, 1.57 (0.82, 2.33) | 36 | 19 | 17 | 0.000048 | NA | NA | NA | No | Yes | 17.39 | IV |
| Mismatch negativity in auditory event-related potentials Duration (long deviant) | 1 | SMD, 1.48 (0.62, 2.34) | 30 | 10 | 20 | 0.00073 | NA | NA | NA | No | Yes | 14.61 | IV |
| Mismatch negativity in auditory event-related potentials Electrode F4 | 1 | SMD, 1.41 (0.79, 2.04) | 50 | 25 | 25 | 0.000009 | NA | NA | NA | No | Yes | 13.01 | IV |
| N400 amplitude for congruent/related condition Word-based | 1 | SMD, -1.21 (-2.1, -0.33) | 24 | 12 | 12 | 0.0071 | NA | NA | NA | No | Yes | 0.11 | IV |
| Mismatch negativity in auditory event-related potentials Electrode F3 | 1 | SMD, 1.18 (0.58, 1.79) | 50 | 25 | 25 | 0.00012 | NA | NA | NA | No | Yes | 8.58 | IV |
| Mismatch negativity in auditory event-related potentials Varying | 1 | SMD, 1.17 (0.23, 2.11) | 21 | 10 | 11 | 0.015 | NA | NA | NA | No | Yes | 8.33 | IV |
| Mismatch negativity in auditory event-related potentials Duration | 26 | SMD, 1.11 (0.69, 1.54) | 1320 | 654 | 666 | <0.000001 | 87% | -1.08, 3.31 | No | Yes | Yes | 7.52 | IV |
| Mismatch negativity in auditory event-related potentials Frequency | 29 | SMD, 1.06 (0.65, 1.47) | 1211 | 616 | 595 | <0.000001 | 85% | -1.16, 3.28 | No | Yes | No | 6.83 | IV |
| Face processing ERP (N170 latency) | 1 | SMD, 1.04 (0.5, 1.58) | 60 | 30 | 30 | 0.00016 | NA | NA | NA | No | Yes | 6.61 | IV |
| P85 gating ratio | 1 | SMD, 1.02 (0.54, 1.5) | 84 | 28 | 56 | 0.000031 | NA | NA | NA | No | Yes | 6.38 | IV |
| Mismatch negativity in auditory event-related potentials Duration (short deviant) | 1 | SMD, 0.97 (0.17, 1.77) | 30 | 10 | 20 | 0.018 | NA | NA | NA | No | Yes | 5.82 | IV |
| N400 amplitude for congruent/related condition Electrode Cz, metaphor | 1 | SMD, -0.9 (-1.56, -0.25) | 40 | 20 | 20 | 0.0067 | NA | NA | NA | No | Yes | 0.19 | IV |
| N400 peak latency | 7 | SMD, 0.91 (0.5, 1.32) | 218 | 110 | 108 | 0.000012 | 50% | -0.22, 2.05 | No | Yes | Yes | 5.24 | IV |
| Visual Mismatch Negativity | 4 | SMD, 0.8 (0.51, 1.09) | 197 | 98 | 99 | <0.000001 | 0% | 0.16, 1.44 | Yes | No | Yes | 4.27 | IV |
| P200 amplitude target stimulus Electrode Pz | 4 | SMD, 0.8 (0.59, 1) | 400 | 200 | 200 | <0.000001 | 0% | 0.35, 1.24 | No | No | Yes | 4.23 | IV |
| Mismatch negativity in auditory event-related potentials Complex abstract/pattern | 4 | SMD, 0.79 (0.46, 1.11) | 158 | 75 | 83 | 0.000002 | 0% | 0.07, 1.5 | No | No | Yes | 4.16 | IV |
| Microstate class D (coverage) | 5 | SMD, -0.76 (-1.06, -0.46) | 217 | 102 | 115 | 0.000001 | 16% | -1.35, -0.17 | No | No | No | 0.25 | IV |
| P200 amplitude target stimulus Electrode Cz | 4 | SMD, 0.76 (0.53, 0.99) | 400 | 200 | 200 | <0.000001 | 24% | 0.12, 1.4 | No | No | Yes | 3.97 | IV |
| Mismatch negativity in auditory event-related potentials Across all conditions | 8 | SMD, 0.75 (0.5, 1) | 290 | 145 | 145 | <0.000001 | 0% | 0.35, 1.15 | No | Yes | No | 3.90 | IV |
| Microstate class C (ocurrence) | 6 | SMD, 0.75 (0.43, 1.07) | 255 | 122 | 133 | 0.000004 | 33% | -0.03, 1.53 | No | No | No | 3.89 | IV |
| Mismatch negativity in auditory event-related potentials Complex sensory | 15 | SMD, 0.7 (0.48, 0.92) | 771 | 372 | 399 | <0.000001 | 49% | 0.03, 1.37 | Yes | Yes | No | 3.56 | IV |
| Microstate class D (duration) | 6 | SMD, -0.69 (-0.95, -0.43) | 255 | 122 | 133 | <0.000001 | 3% | -1.05, -0.33 | No | No | No | 0.29 | IV |
| Mismatch negativity in auditory event-related potentials Early MMN | 1 | SMD, 0.65 (0.17, 1.13) | 70 | 35 | 35 | 0.0078 | NA | NA | NA | No | Yes | 3.27 | IV |
| Mismatch negativity in auditory event-related potentials Intensity | 3 | SMD, 0.65 (0.24, 1.05) | 154 | 88 | 66 | 0.0019 | 29% | -3.03, 4.33 | No | No | Yes | 3.23 | IV |
| Mismatch negativity in auditory event-related potentials Unpredictable deviant | 1 | SMD, 0.62 (0.1, 1.14) | 60 | 31 | 29 | 0.02 | NA | NA | NA | No | Yes | 3.07 | IV |
| Face processing ERP (N170 amplitude) | 21 | SMD, 0.6 (0.47, 0.74) | 856 | 438 | 418 | <0.000001 | 11% | 0.46, 0.75 | No | Yes | Yes | 2.99 | IV |
| 40-Hz auditory steady-state response (ASSR) - Power | 14 | SMD, -0.6 (-0.85, -0.35) | 549 | 258 | 291 | 0.000003 | 47% | -1.36, 0.16 | No | Yes | No | 0.34 | IV |
| Mismatch negativity in auditory event-related potentials Late MMN | 1 | SMD, 0.58 (0.1, 1.05) | 70 | 35 | 35 | 0.018 | NA | NA | NA | No | Yes | 2.84 | IV |
| Microstate class C (coverage) | 5 | SMD, 0.57 (0.15, 0.98) | 217 | 102 | 115 | 0.0074 | 55% | -0.73, 1.87 | No | No | No | 2.81 | IV |
| Sensory gating (S1-S2 difference) | 4 | SMD, -0.52 (-0.7, -0.33) | 541 | 171 | 370 | <0.000001 | 0% | -0.93, -0.1 | No | No | Yes | 0.39 | IV |
| Face processing ERP (N250 amplitude) | 6 | SMD, 0.49 (0.26, 0.72) | 300 | 149 | 151 | 0.000036 | 0% | 0.16, 0.81 | No | No | No | 2.41 | IV |
| 40-Hz auditory steady-state response (ASSR) - Phase | 11 | SMD, -0.43 (-0.61, -0.24) | 856 | 428 | 428 | 0.000011 | 36% | -0.84, -0.01 | No | No | Yes | 0.46 | IV |
| P200 latency standard stimulus | 7 | SMD, -0.43 (-0.83, -0.02) | 264 | 124 | 140 | 0.037 | 60% | -1.62, 0.77 | No | Yes | No | 0.46 | IV |
| P200 latency target stimulus Electrode Pz | 3 | SMD, 0.42 (0.19, 0.64) | 320 | 160 | 160 | 0.00023 | 0% | -1.02, 1.85 | No | No | Yes | 2.13 | IV |
| P200 amplitude target stimulus Electrode Fz | 4 | SMD, 0.41 (0.09, 0.72) | 400 | 200 | 200 | 0.012 | 60% | -0.87, 1.68 | No | Yes | Yes | 2.09 | IV |
| P300 Amplitude | 56 | SMD, 0.34 (0.09, 0.59) | 3575 | 1789 | 1786 | 0.0086 | 92% | -1.5, 2.17 | Yes | Yes | Yes | 1.84 | IV |
| P200 latency target stimulus Electrode Fz | 3 | SMD, 0.32 (0.1, 0.54) | 320 | 160 | 160 | 0.0044 | 0% | -1.11, 1.75 | No | No | Yes | 1.79 | IV |
| Face processing event-related potentials (P100) | 12 | SMD, 0.31 (0.06, 0.56) | 710 | 352 | 358 | 0.015 | 58% | -0.46, 1.08 | No | No | Yes | 1.75 | IV |
| P200 amplitude standard stimulus Electrode Pz | 5 | SMD, -0.25 (-0.43, -0.08) | 502 | 253 | 249 | 0.005 | 0% | -0.54, 0.03 | No | No | Yes | 0.63 | IV |
| P200 latency target stimulus Electrode Cz | 3 | SMD, 0.23 (0, 0.45) | 320 | 160 | 160 | 0.048 | 0% | -1.28, 1.73 | No | Yes | No | 1.51 | IV |
| N400 effect | 5 | SMD, 0.77 (-0.98, 2.52) | 170 | 84 | 86 | 0.39 | 93% | -6.04, 7.58 | No | No | No | 4.05 | ns |
| N400 amplitude for congruent/related condition Sentence-based | 1 | SMD, -0.77 (-1.61, 0.06) | 24 | 12 | 12 | 0.069 | NA | NA | NA | No | No | 0.25 | ns |
| N400 amplitude for incongruent/unrelated condition Electrode Cz, incongruous | 1 | SMD, -0.56 (-1.2, 0.07) | 40 | 20 | 20 | 0.081 | NA | NA | NA | No | No | 0.36 | ns |
| N400 amplitude for incongruent/unrelated condition Sentence-based | 1 | SMD, -0.55 (-1.36, 0.27) | 24 | 12 | 12 | 0.19 | NA | NA | NA | No | No | 0.37 | ns |
| N400 amplitude for congruent/related condition Electrode Cz, literal | 1 | SMD, -0.48 (-1.1, 0.15) | 40 | 20 | 20 | 0.14 | NA | NA | NA | No | No | 0.42 | ns |
| Microstate class A (ocurrence) | 6 | SMD, 0.44 (-0.03, 0.91) | 255 | 122 | 133 | 0.067 | 70% | -1.07, 1.94 | No | Yes | No | 2.21 | ns |
| Face processing ERP (N170 latency) Gender identification | 1 | SMD, 0.42 (-0.12, 0.97) | 53 | 26 | 27 | 0.13 | NA | NA | NA | No | No | 2.15 | ns |
| Face processing ERP (N170 latency) Emotion identification | 1 | SMD, 0.4 (-0.14, 0.95) | 53 | 26 | 27 | 0.15 | NA | NA | NA | No | No | 2.08 | ns |
| P200 latency target stimulus | 2 | SMD, 0.4 (-0.66, 1.45) | 68 | 34 | 34 | 0.46 | 78% | NA | NA | Yes | Yes | 2.06 | ns |
| Microstate class B (duration) | 6 | SMD, -0.37 (-0.8, 0.07) | 255 | 122 | 133 | 0.096 | 65% | -1.73, 0.99 | No | No | No | 0.51 | ns |
| P200 amplitude standard stimulus | 7 | SMD, -0.36 (-0.73, 0.01) | 284 | 134 | 150 | 0.054 | 57% | -1.43, 0.71 | Yes | No | Yes | 0.52 | ns |
| N400 amplitude for incongruent/unrelated condition Word-based | 1 | SMD, -0.35 (-1.15, 0.46) | 24 | 12 | 12 | 0.4 | NA | NA | NA | No | No | 0.53 | ns |
| Microstate class D (ocurrence) | 6 | SMD, -0.32 (-0.73, 0.09) | 255 | 122 | 133 | 0.13 | 62% | -1.58, 0.94 | No | No | No | 0.56 | ns |
| Face processing ERP (N170 latency) Happy faces | 1 | SMD, -0.27 (-1.13, 0.59) | 21 | 11 | 10 | 0.54 | NA | NA | NA | No | No | 0.62 | ns |
| Face processing ERP (N170 latency) Fearful faces | 1 | SMD, -0.24 (-1.1, 0.62) | 21 | 11 | 10 | 0.58 | NA | NA | NA | No | No | 0.64 | ns |
| P200 amplitude standard stimulus Electrode Cz | 5 | SMD, -0.25 (-0.51, 0.02) | 502 | 253 | 249 | 0.067 | 50% | -1.04, 0.55 | No | Yes | No | 0.64 | ns |
| P200 amplitude target stimulus | 3 | SMD, 0.22 (-0.14, 0.58) | 125 | 69 | 56 | 0.22 | 0% | -2.11, 2.55 | No | No | No | 1.50 | ns |
| Microstate class A (duration) | 6 | SMD, -0.18 (-0.51, 0.14) | 255 | 122 | 133 | 0.27 | 40% | -1.03, 0.66 | No | No | No | 0.72 | ns |
| P200 latency standard stimulus Electrode Pz | 3 | SMD, -0.18 (-0.91, 0.55) | 282 | 141 | 141 | 0.63 | 83% | -9.15, 8.79 | No | No | Yes | 0.72 | ns |
| Microstate class A (coverage) | 5 | SMD, 0.18 (-0.2, 0.55) | 217 | 102 | 115 | 0.36 | 47% | -0.93, 1.28 | No | No | No | 1.37 | ns |
| MMN - P3b difference | 15 | SMD, 0.15 (-0.05, 0.36) | 969 | 491 | 478 | 0.14 | 52% | -0.5, 0.81 | No | No | Yes | 1.32 | ns |
| N400 amplitude for congruent/related condition | 4 | SMD, -0.15 (-0.51, 0.22) | 118 | 58 | 60 | 0.43 | 0% | -0.95, 0.65 | No | No | No | 0.77 | ns |
| Microstate class B (ocurrence) | 6 | SMD, 0.13 (-0.19, 0.46) | 255 | 122 | 133 | 0.43 | 39% | -0.73, 0.99 | No | No | No | 1.27 | ns |
| P200 latency standard stimulus Electrode Fz | 3 | SMD, -0.13 (-0.92, 0.66) | 282 | 141 | 141 | 0.75 | 85% | -9.9, 9.64 | No | No | Yes | 0.79 | ns |
| P50 Latency | 11 | SMD, 0.13 (-0.05, 0.31) | 483 | 232 | 251 | 0.17 | 0% | -0.08, 0.34 | No | No | No | 1.26 | ns |
| Face processing ERP (N170 latency) Right hemisphere | 1 | SMD, -0.11 (-0.83, 0.6) | 30 | 15 | 15 | 0.76 | NA | NA | NA | No | No | 0.81 | ns |
| Microstate class C (duration) | 6 | SMD, 0.11 (-0.27, 0.49) | 255 | 122 | 133 | 0.56 | 56% | -1, 1.23 | No | No | No | 1.23 | ns |
| P200 amplitude standard stimulus Electrode Fz | 5 | SMD, -0.1 (-0.28, 0.08) | 502 | 253 | 249 | 0.26 | 0% | -0.42, 0.21 | No | No | No | 0.83 | ns |
| Face processing ERP (N170 latency) Neutral faces | 1 | SMD, -0.09 (-0.95, 0.77) | 21 | 11 | 10 | 0.84 | NA | NA | NA | No | No | 0.85 | ns |
| Mismatch negativity in auditory event-related potentials Pitch | 9 | SMD, 0.08 (-0.33, 0.49) | 520 | 234 | 286 | 0.7 | 78% | -1.33, 1.49 | No | No | No | 1.16 | ns |
| Face processing ERP (N170 latency) Left hemisphere | 1 | SMD, -0.06 (-0.78, 0.66) | 30 | 15 | 15 | 0.87 | NA | NA | NA | No | No | 0.90 | ns |
| Mismatch negativity in auditory event-related potentials Predictable deviant | 1 | SMD, -0.04 (-0.54, 0.47) | 60 | 31 | 29 | 0.88 | NA | NA | NA | No | No | 0.93 | ns |
| N400 amplitude for incongruent/unrelated condition | 4 | SMD, -0.02 (-0.5, 0.45) | 118 | 58 | 60 | 0.93 | 40% | -1.7, 1.66 | No | No | No | 0.96 | ns |
| Microstate class B (coverage) | 5 | SMD, 0.01 (-0.41, 0.43) | 217 | 102 | 115 | 0.96 | 57% | -1.32, 1.35 | No | No | No | 1.02 | ns |
| P200 latency standard stimulus Electrode Cz | 3 | SMD, 0.01 (-0.48, 0.5) | 282 | 141 | 141 | 0.97 | 72% | -5.59, 5.6 | Yes | No | Yes | 1.01 | ns |

CI – confidence interval, CE – class of evidence, eOR – equivalent odds ratio, Egger – significant Egger test, ERP - event-related potentials, ES – effect size, ESB – excess significance bias, k – number of studies for each fact, LS - largest study with significant effect, MMN - mismatch negativity, N – total number of cases, NA – not assessable, ns – not significant, OR – odds ratio, PI – prediction interval, SMD – standardized mean difference.

**sTable 4.** Level of evidence for the association of neuroimaging biomarkers and psychotic disorders^57–97^

|  | |  |  | **Features used for classification of level of evidence** | | | | | | | | |  | |  |
| --- | --- | --- | --- | --- | --- | --- | --- | --- | --- | --- | --- | --- | --- | --- | --- |
| **Factor** | **k** | | **ES (95% CI)** | **N** | **Cases** | **Controls** | **p** | **I^2^** | **PI 95% CI** | **Egger** | **ESB** | **LS** | | **eOR** | **Class** |
| Ventricle brain ratio | 72 | | SMD, 0.61 (0.5, 0.71) | 6099 | 3463 | 2636 | <0.000001 | 68% | (-0.14, 1.35) | No | Yes | Yes | | 3.00 | II |
| Frontal N-Acetylaspartate (NAA) | 68 | | SMD, -0.34 (-0.47, -0.2) | 2868 | 1444 | 1424 | 0.000001 | 63% | (-1.22, 0.55) | No | Yes | No | | 0.54 | III |
| Right globus pallidus gray matter (GM) volume | 2 | | SMD, 1.34 | 84 | 36 | 48 | 0.000478 | NA | NA | NA | NA | NA | | 11.36 | IV |
| Left Heschl gyrus gray matter (GM) volume, FEP, longitudinal | 3 | | SMD, -1.33 (-1.77, -0.9) | 97 | 42 | 55 | <0.000001 | NA | NA | NA | NA | NA | | 0.09 | IV |
| Left planum temporal gray matter (GM) volume, longitudinal | 3 | | SMD, -1.18 (-2.13, -0.23) | 101 | 42 | 59 | 0.01 | NA | NA | NA | NA | NA | | 0.12 | IV |
| Left posterior superior temporal gray matter (GM) volume, longitudinal | 5 | | SMD, -1.14 (-1.67, -0.62) | 177 | 81 | 96 | 0.000021 | NA | NA | NA | NA | NA | | 0.13 | IV |
| Left globus pallidus gray matter (GM) volume | 2 | | SMD, 1.06 | 84 | 36 | 48 | 0.000002 | NA | NA | NA | NA | NA | | 6.84 | IV |
| Left Heschl gyrus gray matter (GM) volume, longitudinal | 4 | | SMD, -1.05 (-1.68, -0.43) | 125 | 53 | 72 | 0.001 | NA | NA | NA | NA | NA | | 0.15 | IV |
| Striatum dopamine synthesis capacity | 11 | | SMD, 0.87 (0.59, 1.14) | 244 | 113 | 131 | <0.000001 | NA | NA | NA | NA | NA | | 4.82 | IV |
| Right body ventricle volume | 3 | | SMD, 0.86 | 129 | 79 | 50 | 0.000002 | NA | NA | NA | NA | NA | | 4.76 | IV |
| Frontal cognition activation | 20 | | SMD, -0.81 (-1.06, -0.57) | 879 | 413 | 466 | <0.000001 | NA | NA | NA | NA | NA | | 0.23 | IV |
| Left superior temporal gray matter (GM) volume, longitudinal | 6 | | SMD, -0.8 (-1.55, -0.04) | 179 | 79 | 100 | 0.03 | NA | NA | NA | NA | NA | | 0.23 | IV |
| Occipital gray matter (GM) volume, early onset, longitudinal | 3 | | SMD, -0.8 (-1.58, -0.01) | 135 | 63 | 72 | 0.047296 | NA | NA | NA | NA | NA | | 0.24 | IV |
| Left body ventricle volume | 3 | | SMD, 0.78 | 129 | 79 | 50 | 0.000008 | NA | NA | NA | NA | NA | | 4.12 | IV |
| Anterior cingulate cortex (ACC) gray matter (GM) volume | 7 | | SMD, -0.7 | 447 | 212 | 235 | <0.001 | NA | NA | NA | NA | NA | | 0.28 | IV |
| Left anterior superior temporal gray matter (GM) volume, longitudinal | 5 | | SMD, -0.71 (-1.23, -0.2) | 177 | 81 | 96 | 0.006 | NA | NA | NA | NA | NA | | 0.28 | IV |
| Posterior segment superior temporal asymmetry | 5 | | SMD, 0.7 (0.4, 1) | 238 | <1000 | <1000 | 0.000005 | NA | NA | NA | NA | NA | | 3.56 | IV |
| Left parahippocampus gray matter (GM) volume | 8 | | SMD, -0.69 | 353 | 185 | 168 | 0.000717 | NA | NA | NA | NA | NA | | 0.29 | IV |
| Thalamus gray matter (GM) volume, FEP naïve | 7 | | SMD, -0.68 | 412 | 152 | 260 | 0.00083 | NA | NA | NA | NA | NA | | 0.29 | IV |
| Basal ganglia glutamine/glutamate (Glx), FEP | 3 | | SMD, 0.66 (0.28, 1.03) | 115 | 59 | 56 | 0.000562 | NA | NA | NA | NA | NA | | 3.31 | IV |
| Frontal resting-state activation | 32 | | SMD, -0.65 (-0.88, -0.42) | 971 | 500 | 471 | <0.000001 | NA | NA | NA | NA | NA | | 0.31 | IV |
| Right posterior superior temporal gray matter (GM) volume, longitudinal | 5 | | SMD, -0.62 (-0.92, -0.32) | 177 | 81 | 96 | 0.000051 | NA | NA | NA | NA | NA | | 0.32 | IV |
| Sylvian fissure assymetry | 3 | | SMD, -0.62 (-1.04, -0.2) | 185 | <1000 | <1000 | 0.003812 | NA | NA | NA | NA | NA | | 0.32 | IV |
| Third ventricle volume, medicated | 20 | | SMD, 0.6 | 1593 | 820 | 773 | <0.000001 | NA | NA | NA | NA | NA | | 2.97 | IV |
| Third ventricle volume | 22 | | SMD, 0.59 | 1143 | 595 | 548 | <0.000001 | NA | NA | NA | NA | NA | | 2.92 | IV |
| Left occipital horn volume | 3 | | SMD, 0.58 | 129 | 79 | 50 | 0.000148 | NA | NA | NA | NA | NA | | 2.86 | IV |
| Brain gray matter (GM) volume, FEP, longitudinal | 7 | | SMD, -0.58 (-0.9, -0.26) | 678 | 341 | 337 | 0.000382 | NA | NA | NA | NA | NA | | 0.35 | IV |
| Right hippocampus gray matter (GM) volume | 30 | | SMD, -0.58 (-0.74, -0.41) | 1814 | 922 | 892 | <0.000001 | NA | NA | NA | NA | NA | | 0.35 | IV |
| Superior temporal gray matter (GM) volume, medicated | 14 | | SMD, -0.58 | 1181 | 580 | 601 | <0.000001 | NA | NA | NA | NA | NA | | 0.35 | IV |
| Temporal N-Acetylaspartate (NAA) | 33 | | SMD, -0.58 | 1475 | 756 | 719 | <0.0001 | NA | NA | NA | NA | NA | | 0.35 | IV |
| Right occipital horn volume | 3 | | SMD, 0.57 | 129 | 79 | 50 | 0.001429 | NA | NA | NA | NA | NA | | 2.81 | IV |
| Left hippocampus gray matter (GM) volume | 31 | | SMD, -0.55 (-0.74, -0.36) | 1919 | 974 | 945 | <0.000001 | NA | NA | NA | NA | NA | | 0.37 | IV |
| Left superior temporal gyrus (STG) gray matter (GM) volume | 17 | | SMD, -0.55 (-0.72, -0.38) | 1152 | 605 | 547 | <0.000001 | NA | NA | NA | NA | NA | | 0.37 | IV |
| Striatum D2 receptor density | 13 | | SMD, 0.54 (0.03, 1.07) | 343 | 170 | 173 | 0.041816 | NA | NA | NA | NA | NA | | 2.66 | IV |
| Left frontal cognition activation | 12 | | SMD, -0.54 (-0.78, -0.3) | 390 | 178 | 212 | 0.00001 | NA | NA | NA | NA | NA | | 0.38 | IV |
| Parietal white matter (WM) volume | 4 | | SMD, -0.53 (-0.84, -0.23) | 227 | 112 | 115 | 0.001 | NA | NA | NA | NA | NA | | 0.38 | IV |
| Right frontal cognition activation | 11 | | SMD, -0.54 (-0.9, -0.18) | 397 | 193 | 204 | 0.003283 | NA | NA | NA | NA | NA | | 0.38 | IV |
| Left temporal horn volume | 13 | | SMD, 0.53 | 791 | 424 | 367 | 0.00001 | NA | NA | NA | NA | NA | | 2.62 | IV |
| Brain gray matter (GM) volume | 12 | | SMD, -0.52 (-0.76, -0.28) | 928 | 405 | 523 | 0.000022 | NA | NA | NA | NA | NA | | 0.39 | IV |
| Fusiform gray matter (GM) volume, medicated | 8 | | SMD, -0.52 | 690 | 337 | 353 | 0.000012 | NA | NA | NA | NA | NA | | 0.39 | IV |
| Left lateral ventricle volume | 18 | | SMD, 0.51 | 1053 | 557 | 496 | <0.000001 | NA | NA | NA | NA | NA | | 2.52 | IV |
| Cerebellum N-Acetylaspartate (NAA) | 5 | | SMD, -0.5 | 183 | 108 | 75 | 0.0114 | NA | NA | NA | NA | NA | | 0.40 | IV |
| Frontal white matter (WM) volume | 5 | | SMD, -0.51 (-0.76, -0.26) | 323 | 185 | 138 | 0.00006 | NA | NA | NA | NA | NA | | 0.40 | IV |
| Planum temporale asymmetry | 10 | | SMD, -0.51 (-1.04, -0.02) | 368 | <1000 | <1000 | 0.05 | NA | NA | NA | NA | NA | | 0.40 | IV |
| Frontal gray matter (GM) volume, medicated | 13 | | SMD, -0.49 | 1288 | 613 | 675 | <0.000001 | NA | NA | NA | NA | NA | | 0.41 | IV |
| Temporal white matter (WM) volume | 6 | | SMD, -0.48 (-0.76, -0.21) | 259 | 128 | 131 | 0.001 | NA | NA | NA | NA | NA | | 0.41 | IV |
| Left lateral ventricle volume, FEP | 12 | | SMD, 0.49 (0.35, 0.64) | 825 | 396 | 429 | <0.000001 | NA | NA | NA | NA | NA | | 2.43 | IV |
| Ventricles volumen | 30 | | SMD, 0.49 | 1896 | 984 | 912 | <0.000001 | NA | NA | NA | NA | NA | | 2.43 | IV |
| Frontal volumen | 3 | | SMD, -0.48 (-0.78, -0.18) | 226 | 132 | 94 | 0.002 | NA | NA | NA | NA | NA | | 0.42 | IV |
| Left frontal resting-state activation | 23 | | SMD, -0.48 (-0.8, -0.15) | 617 | 356 | 261 | 0.003795 | NA | NA | NA | NA | NA | | 0.42 | IV |
| Left insula gray matter (GM) volume | 15 | | SMD, -0.45 (-0.67, -0.24) | 844 | <1000 | <1000 | 0.00004 | NA | NA | NA | NA | NA | | 0.44 | IV |
| Cerebrospinal fluid volume | 3 | | SMD, 0.45 (0.09, 0.81) | 158 | 60 | 98 | 0.01475 | NA | NA | NA | NA | NA | | 2.27 | IV |
| Lateral ventricles volume, longitudinal | 13 | | SMD, 0.45 (0.19, 0.71) | 821 | 473 | 348 | 0.05 | NA | NA | NA | NA | NA | | 2.26 | IV |
| Basal ganglia N-acetylaspartate to creatine ratio (NAA/Cr) | 4 | | SMD, -0.44 (-0.83, -0.06) | NA | - | - | 0.02 | NA | NA | NA | NA | NA | | 0.45 | IV |
| Frontal gray matter (GM) volume, early onset, longitudinal | 3 | | SMD, -0.44 (-0.68, -0.19) | 268 | 121 | 147 | 0.000438 | NA | NA | NA | NA | NA | | 0.45 | IV |
| Insula gray matter (GM) volume | 17 | | SMD, -0.45 (-0.64, -0.25) | 945 | <1000 | <1000 | 0.00001 | NA | NA | NA | NA | NA | | 0.45 | IV |
| Insula gray matter (GM) volume, medicated | 11 | | SMD, -0.44 | 856 | 415 | 441 | 0.00027 | NA | NA | NA | NA | NA | | 0.45 | IV |
| Prefrontal gray matter (GM) volume, medicated | 14 | | SMD, -0.44 | 1263 | 659 | 604 | <0.000001 | NA | NA | NA | NA | NA | | 0.45 | IV |
| Right insula gray matter (GM) volume | 15 | | SMD, -0.44 (-0.65, -0.23) | 844 | <1000 | <1000 | 0.00004 | NA | NA | NA | NA | NA | | 0.45 | IV |
| Cortex gray matter (GM) volume, medicated | 12 | | SMD, -0.43 | 987 | 495 | 492 | <0.000001 | NA | NA | NA | NA | NA | | 0.46 | IV |
| Hippocampus gray matter (GM) volume, FEP naïve | 8 | | SMD, -0.43 | 445 | 194 | 251 | 0.000008 | NA | NA | NA | NA | NA | | 0.46 | IV |
| Right frontal resting-state activation | 23 | | SMD, -0.43 (-0.74, -0.12) | 617 | 356 | 261 | 0.006555 | NA | NA | NA | NA | NA | | 0.46 | IV |
| Temporal gray matter (GM) volume, medicated | 17 | | SMD, -0.43 | 1433 | 717 | 716 | 0.000001 | NA | NA | NA | NA | NA | | 0.46 | IV |
| Frontal white matter (WM) glutamine/glutamate (Glx) | 7 | | SMD, 0.42 (0.18, 0.66) | 310 | 200 | 110 | 0.000604 | NA | NA | NA | NA | NA | | 2.14 | IV |
| Genu corpus callosum FA, females | 10 | | SMD, -0.42 (-0.69, -0.15) | 1105 | 589 | 516 | 0.002379 | NA | NA | NA | NA | NA | | 0.47 | IV |
| Left arcuate fasciculus FA, patients with auditory-verbal hallucinations | 5 | | SMD, -0.42 (-0.69, -0.16) | 256 | 106 | 150 | 0.001894 | NA | NA | NA | NA | NA | | 0.47 | IV |
| Planum temporal gray matter (GM) volume, medicated | 14 | | SMD, -0.42 | 849 | 444 | 405 | 0.0015 | NA | NA | NA | NA | NA | | 0.47 | IV |
| Prefrontal N-acetylaspartate to creatine ratio (NAA/Cr), subjects at risk | 9 | | SMD, -0.42 (-0.61, -0.23) | 442 | 208 | 234 | 0.000015 | NA | NA | NA | NA | NA | | 0.47 | IV |
| Inferior frontal gray matter (GM) volume, medicated | 8 | | SMD, -0.41 | 657 | 342 | 315 | <0.000001 | NA | NA | NA | NA | NA | | 0.48 | IV |
| Left anterior superior temporal gray matter (GM) volume | 8 | | SMD, -0.41 | 377 | 194 | 183 | 0.015726 | NA | NA | NA | NA | NA | | 0.48 | IV |
| Left HAC gray matter (GM) volume | 23 | | SMD, -0.41 (-0.74, -0.41) | 1302 | 695 | 607 | 0.000001 | NA | NA | NA | NA | NA | | 0.48 | IV |
| Left posterior superior temporal gray matter (GM) volume | 5 | | SMD, -0.4 | 222 | 94 | 128 | 0.027694 | NA | NA | NA | NA | NA | | 0.48 | IV |
| Left thalamus gray matter (GM) volume | 13 | | SMD, -0.4 (-0.53, -0.26) | 957 | 449 | 508 | <0.000001 | NA | NA | NA | NA | NA | | 0.48 | IV |
| Right frontal volume | 33 | | SMD, -0.41 (-0.56, -0.26) | 1951 | 979 | 972 | <0.000001 | NA | NA | NA | NA | NA | | 0.48 | IV |
| Right parahippocampus gray matter (GM) volume | 8 | | SMD, -0.4 | 353 | 185 | 168 | 0.012339 | NA | NA | NA | NA | NA | | 0.48 | IV |
| Right superior temporal gyrus (STG) gray matter (GM) volume | 16 | | SMD, -0.4 (-0.4, -0.65) | 1122 | 590 | 532 | <0.000001 | NA | NA | NA | NA | NA | | 0.48 | IV |
| Medial frontal glutamine (Gln) | 8 | | SMD, 0.4 | 275 | 140 | 135 | 0.045 | NA | NA | NA | NA | NA | | 2.08 | IV |
| Medial temporal glutamine/glutamate (Glx) | 8 | | SMD, 0.4 (0.08, 0.71) | 376 | 197 | 179 | 0.012816 | NA | NA | NA | NA | NA | | 2.07 | IV |
| Right lateral ventricle volume, FEP | 12 | | SMD, 0.4 (0.26, 0.54) | 825 | 396 | 429 | <0.000001 | NA | NA | NA | NA | NA | | 2.07 | IV |
| Right temporal horn volume | 13 | | SMD, 0.4 | 791 | 424 | 367 | 0.000208 | NA | NA | NA | NA | NA | | 2.07 | IV |
| Frontal gray matter (GM) volume, FEP, longitudinal | 4 | | SMD, -0.39 (-0.57, -0.22) | 533 | 294 | 239 | 0.000013 | NA | NA | NA | NA | NA | | 0.49 | IV |
| Left amygdala gray matter (GM) volume | 15 | | SMD, -0.39 (-0.68, -0.1) | 970 | 481 | 489 | 0.008394 | NA | NA | NA | NA | NA | | 0.49 | IV |
| Medial frontal glutamate (Glu) | 9 | | SMD, -0.39 | 337 | 166 | 171 | 0.006 | NA | NA | NA | NA | NA | | 0.49 | IV |
| Right lateral ventricle volume | 18 | | SMD, 0.39 | 1053 | 557 | 496 | <0.000001 | NA | NA | NA | NA | NA | | 2.03 | IV |
| Caudate gray matter (GM) volume, FEP naïve | 10 | | SMD, -0.38 | 721 | 299 | 422 | 0.000001 | NA | NA | NA | NA | NA | | 0.50 | IV |
| Right amygdala gray matter (GM) volume | 17 | | SMD, -0.38 (-0.72, -0.04) | 1109 | 548 | 561 | 0.028485 | NA | NA | NA | NA | NA | | 0.50 | IV |
| Right thalamus gray matter (GM) volume | 13 | | SMD, -0.38 (-0.52, -0.25) | 957 | 449 | 508 | <0.000001 | NA | NA | NA | NA | NA | | 0.50 | IV |
| Lateral ventricles volume, FEP | 8 | | SMD, 0.38 (0.22, 0.54) | 627 | 308 | 319 | 0.000003 | NA | NA | NA | NA | NA | | 1.99 | IV |
| Absent adhesio interthalamica | 11 | | OR, 1.98 (1.33, 2.94) | 1540 | 822 | 718 | 0.0008 | NA | NA | NA | NA | NA | | 1.98 | IV |
| Frontal cognition relative activation | 17 | | SMD, -0.37 (-0.53, -0.22) | 685 | <1000 | <1000 | 0.000003 | NA | NA | NA | NA | NA | | 0.51 | IV |
| Temporal gray matter (GM) volume, FEP, longitudinal | 4 | | SMD, -0.37 (-0.71, -0.04) | 533 | 294 | 239 | 0.028 | NA | NA | NA | NA | NA | | 0.51 | IV |
| Brain gray matter (GM) volume, FEP | 12 | | SMD, -0.36 (-0.5, -0.23) | 850 | 412 | 438 | <0.000001 | NA | NA | NA | NA | NA | | 0.52 | IV |
| Brain gray matter (GM) volume, naïve | 10 | | SMD, -0.36 | 530 | 238 | 292 | 0.000066 | NA | NA | NA | NA | NA | | 0.52 | IV |
| Right HAC gray matter (GM) volume | 22 | | SMD, -0.36 (-0.54, -0.18) | 1238 | 669 | 569 | 0.000089 | NA | NA | NA | NA | NA | | 0.52 | IV |
| Right frontal horn volume | 3 | | SMD, 0.36 | 129 | 84 | 45 | 0.007158 | NA | NA | NA | NA | NA | | 1.92 | IV |
| Anterior cingulate cortex (ACC) gray matter (GM) volume, medicated | 23 | | SMD, -0.34 | 1919 | 946 | 973 | <0.000001 | NA | NA | NA | NA | NA | | 0.54 | IV |
| Frontal gray matter (GM) volume | 9 | | SMD, -0.34 (-0.66, -0.02) | 503 | 262 | 241 | 0.035 | NA | NA | NA | NA | NA | | 0.54 | IV |
| Left frontal volume | 13 | | SMD, -0.34 | 762 | 395 | 367 | 0.00146 | NA | NA | NA | NA | NA | | 0.54 | IV |
| Thalamus N-Acetylaspartate (NAA) | 17 | | SMD, -0.34 | 706 | 393 | 313 | 0.0002 | NA | NA | NA | NA | NA | | 0.54 | IV |
| Frontal resting-state relative activation | 38 | | SMD, -0.32 (-0.43, -0.21) | 1464 | 854 | 610 | <0.000001 | NA | NA | NA | NA | NA | | 0.56 | IV |
| Medial frontal N-Acetylaspartate (NAA) | 19 | | SMD, -0.32 | 779 | 401 | 378 | 0.019 | NA | NA | NA | NA | NA | | 0.56 | IV |
| Middle frontal gray matter (GM) volume, medicated | 9 | | SMD, -0.32 | 677 | 352 | 325 | 0.000032 | NA | NA | NA | NA | NA | | 0.56 | IV |
| Posterior cingulate gray matter (GM) volume, medicated | 7 | | SMD, -0.32 | 635 | 311 | 324 | 0.0062 | NA | NA | NA | NA | NA | | 0.56 | IV |
| Parietal gray matter (GM) volume, medicated | 8 | | SMD, -0.31 | 758 | 354 | 404 | 0.0076 | NA | NA | NA | NA | NA | | 0.57 | IV |
| Cerebrospinal fluid volume, naïve | 7 | | SMD, 0.31 | 468 | 182 | 286 | 0.011 | NA | NA | NA | NA | NA | | 1.75 | IV |
| Lateral ventricles volume | 11 | | SMD, 0.31 (0.14, 0.47) | 896 | 549 | 347 | 0.000177 | NA | NA | NA | NA | NA | | 1.75 | IV |
| Parietal gray matter (GM) volume, FEP, longitudinal | 3 | | SMD, -0.3 (-0.48, -0.12) | 490 | 277 | 213 | 0.001 | NA | NA | NA | NA | NA | | 0.58 | IV |
| Right temporal volume | 30 | | SMD, -0.3 (-0.42, -0.17) | 1945 | 999 | 946 | 0.000003 | NA | NA | NA | NA | NA | | 0.58 | IV |
| Accumbens gray matter (GM) volume, medicated | 11 | | SMD, -0.29 | 904 | 426 | 478 | 0.017 | NA | NA | NA | NA | NA | | 0.59 | IV |
| Heschl gyrus gray matter (GM) volume, medicated | 11 | | SMD, -0.29 | 637 | 301 | 336 | 0.0043 | NA | NA | NA | NA | NA | | 0.59 | IV |
| Prefrontal white matter (WM) volume, medicated | 11 | | SMD, -0.29 | 965 | 511 | 454 | 0.00001 | NA | NA | NA | NA | NA | | 0.59 | IV |
| Superior frontal gray matter (GM) volume, medicated | 9 | | SMD, -0.29 | 874 | 422 | 452 | 0.000028 | NA | NA | NA | NA | NA | | 0.59 | IV |
| Genu corpus callosum FA, males | 10 | | SMD, -0.28 (-0.47, -0.09) | 1105 | 589 | 516 | 0.003806 | NA | NA | NA | NA | NA | | 0.60 | IV |
| Left hemisphere volume | 15 | | SMD, -0.28 | 897 | 463 | 434 | 0.000104 | NA | NA | NA | NA | NA | | 0.60 | IV |
| Right anterior superior temporal gray matter (GM) volume | 7 | | SMD, -0.28 | 347 | 179 | 168 | 0.006665 | NA | NA | NA | NA | NA | | 0.60 | IV |
| Right hippocampus-amygdala gray matter (GM) volume | 15 | | SMD, -0.28 | 731 | 407 | 324 | 0.000005 | NA | NA | NA | NA | NA | | 0.60 | IV |
| Right hemisphere volume | 15 | | SMD, -0.27 | 897 | 463 | 434 | 0.000104 | NA | NA | NA | NA | NA | | 0.61 | IV |
| Brain resting-state blood flow | 22 | | SMD, -0.26 (-0.4, -0.11) | 795 | <1000 | <1000 | 0.000441 | NA | NA | NA | NA | NA | | 0.62 | IV |
| Brain volume, FEP | 21 | | SMD, -0.26 (-0.4, -0.12) | 1458 | 686 | 772 | 0.000273 | NA | NA | NA | NA | NA | | 0.62 | IV |
| Brain volume, postmortem | 27 | | SMD, -0.26 (-0.35, -0.15) | 1533 | 763 | 770 | <0.000001 | NA | NA | NA | NA | NA | | 0.62 | IV |
| Globus pallidus gray matter (GM) volume, medicated | 11 | | SMD, 0.26 | 1144 | 510 | 634 | 0.034 | NA | NA | NA | NA | NA | | 1.60 | IV |
| Prevalence of large cavum septum pellucidum (CSP) | 15 | | OR, 1.59 (1.07, 2.38) | 1920 | 1054 | 866 | 0.02 | NA | NA | NA | NA | NA | | 1.59 | IV |
| Brain volumen | 31 | | SMD, -0.25 | 1867 | 946 | 921 | 0.000104 | NA | NA | NA | NA | NA | | 0.64 | IV |
| Dorsolateral prefrontal cortex (DLPFC) N-acetylaspartate to creatine ratio (NAA/Cr) | 5 | | SMD, 0.24 (0, 0.49) | NA | - | - | 0.05 | NA | NA | NA | NA | NA | | 1.55 | IV |
| Frontal torque asymmetry | 3 | | SMD, 0.24 (0.15, 0.34) | 383 | <1000 | <1000 | 0.000001 | NA | NA | NA | NA | NA | | 1.55 | IV |
| Corpus callosum area | 28 | | SMD, -0.24 (-0.4, -0.07) | 1703 | 894 | 809 | 0.00436 | NA | NA | NA | NA | NA | | 0.65 | IV |
| Left hippocampus-amygdala gray matter (GM) volume | 15 | | SMD, -0.24 | 731 | 407 | 324 | 0.006109 | NA | NA | NA | NA | NA | | 0.65 | IV |
| Parahippocampus gray matter (GM) volume, medicated | 15 | | SMD, -0.24 | 1129 | 561 | 568 | 0.0021 | NA | NA | NA | NA | NA | | 0.65 | IV |
| Right hippocampus gray matter (GM) volume, clinical high risk | 8 | | SMD, -0.24 (0.01, 0.48) | 1429 | 939 | 490 | 0.045321 | NA | NA | NA | NA | NA | | 0.65 | IV |
| Occipital gray matter (GM) volume, medicated | 8 | | SMD, -0.22 | 700 | 339 | 361 | 0.0041 | NA | NA | NA | NA | NA | | 0.67 | IV |
| Temporal volume, medicated | 20 | | SMD, -0.22 | 1228 | 642 | 586 | 0.00069 | NA | NA | NA | NA | NA | | 0.67 | IV |
| Occipital torque asymmetry | 5 | | SMD, 0.22 (0.12, 0.28) | 579 | <1000 | <1000 | <0.000001 | NA | NA | NA | NA | NA | | 1.49 | IV |
| Brain volume, FEP naïve | 15 | | SMD, -0.21 | 854 | 364 | 490 | 0.003 | NA | NA | NA | NA | NA | | 0.68 | IV |
| Orbitofrontal gray matter (GM) volume, medicated | 15 | | SMD, -0.21 | 1141 | 550 | 591 | 0.01 | NA | NA | NA | NA | NA | | 0.68 | IV |
| Brain white matter (WM) volume, FEP naïve | 10 | | SMD, -0.18 | 530 | 238 | 292 | 0.042 | NA | NA | NA | NA | NA | | 0.72 | IV |
| Left temporal volume | 25 | | SMD, -0.18 | 1362 | 693 | 669 | 0.010065 | NA | NA | NA | NA | NA | | 0.72 | IV |
| Left superior temporal gray matter (GM) volume | 10 | | SMD, -0.17 | 585 | 314 | 271 | 0.019925 | NA | NA | NA | NA | NA | | 0.73 | IV |
| Right amygdala negatively balance emotional manipulation activation | 35 | | SMD, -0.17 (-0.37, -0.03) | NA | - | - | 0.012 | NA | NA | NA | NA | NA | | 0.73 | IV |
| Right superior temporal gray matter (GM) volume | 10 | | SMD, -0.17 | 585 | 314 | 271 | 0.019925 | NA | NA | NA | NA | NA | | 0.73 | IV |
| Cerebellum gray matter (GM) volume, medicated | 17 | | SMD, -0.15 | 1402 | 726 | 676 | 0.035 | NA | NA | NA | NA | NA | | 0.76 | IV |
| Intracranial volume, FEP | 17 | | SMD, -0.15 (-0.27, -0.04) | 1148 | 554 | 594 | 0.010574 | NA | NA | NA | NA | NA | | 0.76 | IV |
| Intracranial volume, FEP naïve | 17 | | SMD, -0.14 | 989 | 414 | 575 | 0.041 | NA | NA | NA | NA | NA | | 0.78 | IV |
| Corpus callosum FA | 22 | | SMD, -0.02 (-0.03, -0.02) | 1411 | 729 | 682 | <0.000001 | NA | NA | NA | NA | NA | | 0.96 | IV |
| Splenium corpus callosum FA | 17 | | SMD, -0.02 (-0.03, -0.01) | 950 | <1000 | <1000 | <0.000001 | NA | NA | NA | NA | NA | | 0.96 | IV |
| Genu corpus callosum FA | 19 | | SMD, -0.02 (-0.03, -0.01) | 984 | <1000 | <1000 | 0.000035 | NA | NA | NA | NA | NA | | 0.97 | IV |
| Brain gray matter (GM) volume, longitudinal | 26 | | [decr] | 1985 | 1102 | 883 | 0.017757 | NA | NA | NA | NA | NA | | NA | IV |
| Extracerebral volume | 17 | | [incr] | 566 | <1000 | <1000 | <0.001 | NA | NA | NA | NA | NA | | NA | IV |
| Frontal gray matter (GM) N-Acetylaspartate (NAA) | 19 | | [ratio minus 1], -0.04 | 1063 | 488 | 575 | <0.0008 | NA | NA | NA | NA | NA | | NA | IV |
| Frontal white matter (WM) N-Acetylaspartate (NAA) | 32 | | [ratio minus 1], -0.08 | 613 | 318 | 295 | <0.0001 | NA | NA | NA | NA | NA | | NA | IV |
| Hippocampus N-acetylaspartate to creatine ratio (NAA/Cr) | 9 | | [ratio minus 1], -0.16 | 268 | 132 | 136 | <0.0001 | NA | NA | NA | NA | NA | | NA | IV |
| Intracranial volume | 20 | | [decr] | 644 | <1000 | <1000 | <0.02 | NA | NA | NA | NA | NA | | NA | IV |
| Right ventral striatum reward anticipation activation | 23 | | SMD, -0.7 | 917 | <1000 | <1000 | 0.001 | NA | NA | NA | NA | NA | | 0.28 | IV |
| Left ventral striatum reward feedback activation | 9 | | SMD, -0.57 | 358 | <1000 | <1000 | 0.001 | NA | NA | NA | NA | NA | | 0.36 | IV |
| Right ventral striatum reward feedback activation | 9 | | SMD, -0.56 | 358 | <1000 | <1000 | 0.001 | NA | NA | NA | NA | NA | | 0.36 | IV |
| Left ventral striatum reward anticipation activation | 23 | | SMD, -0.5 | 917 | <1000 | <1000 | 0.001 | NA | NA | NA | NA | NA | | 0.40 | IV |
| Dorsolateral prefrontal cortex (DLPFC) cognition activation | 11 | | [decr] | 276 | 132 | 144 | <0.001 | NA | NA | NA | NA | NA | | NA | IV |
| AFC cognition activation | 11 | | [decr] | 276 | 132 | 144 | ns | NA | NA | NA | NA | NA | | NA | ns |
| Ventrolateral prefrontal cortex (VLPFC) cognition activation | 11 | | [decr] | 276 | 132 | 144 | ns | NA | NA | NA | NA | NA | | NA | ns |
| Hippocampus choline/creatine (Cho/Cr) | 4 | | SMD, 0.94 (-0.2, 2.07) | NA | - | - | 0.11 | NA | NA | NA | NA | NA | | 5.50 | ns |
| Hippocampus N-Acetylaspartate (NAA) | 7 | | SMD, -0.82 (-1.69, 0.05) | 339 | 150 | 189 | 0.06 | NA | NA | NA | NA | NA | | 0.23 | ns |
| Dorsolateral prefrontal cortex (DLPFC) N-Acetylaspartate (NAA) | 6 | | SMD, -0.46 (-1.09, 0.17) | 312 | 140 | 172 | 0.15 | NA | NA | NA | NA | NA | | 0.43 | ns |
| Anterior cingulate cortex (ACC) choline/creatine (Cho/Cr) | 5 | | SMD, 0.44 (-0.3, 1.17) | NA | - | - | 0.24 | NA | NA | NA | NA | NA | | 2.22 | ns |
| Left temporal cognition activation | 10 | | SMD, 0.43 (-0.16, 1.01) | 480 | 254 | 226 | 0.149682 | NA | NA | NA | NA | NA | | 2.18 | ns |
| Pituitary gland gray matter (GM) volume, FEP | 4 | | SMD, 0.39 (-0.05, 0.84) | 403 | 198 | 205 | 0.085847 | NA | NA | NA | NA | NA | | 2.03 | ns |
| Right planum temporal gray matter (GM) volume, longitudinal | 3 | | SMD, -0.37 (-0.76, 0.02) | 101 | 42 | 59 | 0.06 | NA | NA | NA | NA | NA | | 0.51 | ns |
| Right superior temporal gray matter (GM) volume, longitudinal | 6 | | SMD, -0.35 (-1.13, 0.42) | 179 | 79 | 100 | 0.37 | NA | NA | NA | NA | NA | | 0.53 | ns |
| Dorsolateral prefrontal cortex (DLPFC) Glutamine/glutamate (Glx) | 8 | | SMD, -0.32 (-0.85, 0.21) | 304 | 172 | 132 | 0.236661 | NA | NA | NA | NA | NA | | 0.56 | ns |
| Right anterior superior temporal gray matter (GM) volume, longitudinal | 5 | | SMD, -0.32 (-0.82, 0.18) | 177 | 81 | 96 | 0.21 | NA | NA | NA | NA | NA | | 0.56 | ns |
| Thalamus D2 D3 availability | 8 | | SMD, -0.32 (-0.68, 0.03) | 264 | 138 | 126 | 0.077274 | NA | NA | NA | NA | NA | | 0.56 | ns |
| Left frontal horn volumen | 3 | | SMD, 0.29 | 129 | 84 | 45 | 0.119944 | NA | NA | NA | NA | NA | | 1.69 | ns |
| Thalamus glutamate (Glu) | 3 | | SMD, -0.29 | 128 | 64 | 64 | 0.2 | NA | NA | NA | NA | NA | | 0.60 | ns |
| Frontal N-acetylaspartate to creatine ratio (NAA/Cr) | 7 | | SMD, -0.27 (-0.63, 0.08) | NA | - | - | 0.13 | NA | NA | NA | NA | NA | | 0.61 | ns |
| Superior temporal volume, medicated | 12 | | SMD, -0.27 | 800 | 394 | 406 | 0.058 | NA | NA | NA | NA | NA | | 0.61 | ns |
| Left hippocampus gray matter (GM) volume, clinical high risk | 8 | | SMD, -0.25 (-0.04, 0.54) | 1429 | 939 | 490 | 0.0911 | NA | NA | NA | NA | NA | | 0.64 | ns |
| Striatum dopamine transporter density | 13 | | SMD, -0.24 (-0.68, 0.19) | 349 | 202 | 147 | 0.269 | NA | NA | NA | NA | NA | | 0.64 | ns |
| Right putamen gray matter (GM) volume | 7 | | SMD, 0.24 | 320 | 169 | 151 | 0.221551 | NA | NA | NA | NA | NA | | 1.55 | ns |
| Parietal gray matter (GM) volume, early onset, longitudinal | 3 | | SMD, -0.24 (-0.48, 0) | 268 | 121 | 147 | 0.05243 | NA | NA | NA | NA | NA | | 0.65 | ns |
| Brain gamma-aminobutyric acid (GABA) | 10 | | SMD, -0.23 (-0.48, 0.04) | 549 | 277 | 272 | 0.089246 | NA | NA | NA | NA | NA | | 0.66 | ns |
| Left Dorsolateral prefrontal cortex (DLPFC) working memory activation | 28 | | SMD, -0.23 (-0.05, 0.51) | 776 | <1000 | <1000 | 0.11 | NA | NA | NA | NA | NA | | 0.66 | ns |
| Splenium corpus callosum FA, females | 10 | | SMD, -0.23 (-0.55, 0.09) | 1105 | 589 | 516 | 0.15597 | NA | NA | NA | NA | NA | | 0.66 | ns |
| Temporal cortex D2 D3 availability | 6 | | SMD, -0.23 (-0.54, 0.07) | 170 | 84 | 86 | 0.139406 | NA | NA | NA | NA | NA | | 0.66 | ns |
| Anterior cingulate cortex (ACC) N-Acetylaspartate (NAA) | 10 | | SMD, -0.22 (-0.81, 0.38) | 431 | 234 | 197 | 0.48 | NA | NA | NA | NA | NA | | 0.67 | ns |
| Fourth ventricle volume | 5 | | SMD, 0.21 | 253 | 119 | 134 | 0.216883 | NA | NA | NA | NA | NA | | 1.46 | ns |
| Left putamen gray matter (GM) volume | 7 | | SMD, 0.21 | 320 | 169 | 151 | 0.144509 | NA | NA | NA | NA | NA | | 1.46 | ns |
| Superior temporal asymmetry | 15 | | SMD, 0.21 (-0.08, 0.51) | 1020 | - | - | 0.162947 | NA | NA | NA | NA | NA | | 1.46 | ns |
| Splenium corpus callosum FA, males | 10 | | SMD, -0.2 (-0.42, 0.01) | 1105 | 589 | 516 | 0.064231 | NA | NA | NA | NA | NA | | 0.69 | ns |
| Dorsolateral prefrontal cortex (DLPFC) working memory activation | 30 | | SMD, -0.2 (-0.05, 0.44) | 808 | <1000 | <1000 | 0.13 | NA | NA | NA | NA | NA | | 0.70 | ns |
| Frontal gamma-aminobutyric acid (GABA) | 7 | | SMD, -0.2 (-0.56, 0.16) | 489 | 246 | 243 | 0.287726 | NA | NA | NA | NA | NA | | 0.70 | ns |
| Right posterior superior temporal gray matter (GM) volume | 4 | | SMD, 0.19 | 192 | 79 | 113 | 0.233063 | NA | NA | NA | NA | NA | | 1.41 | ns |
| Hippocampus choline-containing compounds (Cho) | 7 | | SMD, -0.19 (-1.09, 0.71) | 339 | 150 | 189 | 0.68 | NA | NA | NA | NA | NA | | 0.71 | ns |
| Right arcuate fasciculus FA, patients with auditory-verbal hallucinations | 5 | | SMD, -0.19 (-0.47, 0.09) | 256 | 106 | 150 | 0.183526 | NA | NA | NA | NA | NA | | 0.71 | ns |
| Right Dorsolateral prefrontal cortex (DLPFC) working memory activation | 28 | | SMD, -0.15 (-0.13, 0.42) | 776 | <1000 | <1000 | 0.34 | NA | NA | NA | NA | NA | | 0.76 | ns |
| Basal ganglia choline-containing compounds (Cho) | 6 | | SMD, 0.15 (-0.37, 0.68) | 225 | 120 | 105 | 0.57 | NA | NA | NA | NA | NA | | 1.31 | ns |
| Dorsolateral prefrontal cortex (DLPFC) choline-containing compounds (Cho) | 6 | | SMD, 0.15 (-0.44, 0.74) | 312 | 140 | 172 | 0.62 | NA | NA | NA | NA | NA | | 1.31 | ns |
| Frontal choline/creatine (Cho/Cr) | 5 | | SMD, 0.14 (-0.34, 0.61) | NA | - | - | 0.58 | NA | NA | NA | NA | NA | | 1.29 | ns |
| Brain white matter (WM) volume, FEP | 6 | | SMD, -0.14 (-0.32, 0.03) | 493 | 233 | 260 | 0.116888 | NA | NA | NA | NA | NA | | 0.78 | ns |
| Occipital gray matter (GM) volume, longitudinal | 6 | | SMD, -0.14 (-0.26, 0.55) | 590 | 321 | 269 | 0.48 | NA | NA | NA | NA | NA | | 0.78 | ns |
| Righ Heschl gyrus gray matter (GM) volume, FEP, longitudinal | 3 | | SMD, -0.14 (-0.53, 0.25) | 97 | 42 | 55 | 0.48 | NA | NA | NA | NA | NA | | 0.78 | ns |
| Frontal white matter (WM) volume, medicated | 11 | | SMD, -0.13 | 1052 | 506 | 546 | 0.14 | NA | NA | NA | NA | NA | | 0.79 | ns |
| Left amygdala negatively balance emotional manipulation activation | 35 | | SMD, -0.13 (-0.31, 0.04) | NA | - | - | 0.136 | NA | NA | NA | NA | NA | | 0.79 | ns |
| Left hippocampus cognition and resting-state activation | 10 | | SMD, -0.13 (-0.69, 0.43) | 415 | 190 | 225 | 0.649115 | NA | NA | NA | NA | NA | | 0.79 | ns |
| Left temporal resting-state activation | 19 | | SMD, -0.13 (-0.5, 0.23) | 608 | 307 | 301 | 0.485134 | NA | NA | NA | NA | NA | | 0.79 | ns |
| Occipital gray matter (GM) volume, FEP, longitudinal | 3 | | SMD, -0.13 (-0.31, 0.05) | 490 | 277 | 213 | 0.15 | NA | NA | NA | NA | NA | | 0.79 | ns |
| Right Heschl gyrus gray matter (GM) volume, longitudinal | 4 | | SMD, -0.13 (-0.48, 0.21) | 125 | 53 | 72 | 0.44 | NA | NA | NA | NA | NA | | 0.79 | ns |
| Thalamus choline-containing compounds (Cho) | 8 | | SMD, -0.13 (-0.41, 0.16) | 309 | 171 | 138 | 0.38 | NA | NA | NA | NA | NA | | 0.79 | ns |
| Caudate gray matter (GM) volume | 9 | | SMD, 0.12 (-0.11, 0.34) | 363 | 192 | 171 | 0.30795 | NA | NA | NA | NA | NA | | 1.23 | ns |
| Temporal horn lateral ventricle asymmetry | 8 | | SMD, -0.11 (-0.61, 0.4) | 629 | <1000 | <1000 | 0.669436 | NA | NA | NA | NA | NA | | 0.82 | ns |
| Pituitary gland gray matter (GM) volume | 9 | | SMD, 0.1 (-0.2, 0.4) | 1225 | 617 | 608 | 0.513549 | NA | NA | NA | NA | NA | | 1.20 | ns |
| Putamen gray matter (GM) volume, medicated | 21 | | SMD, 0.1 | 1956 | 950 | 1006 | 0.24 | NA | NA | NA | NA | NA | | 1.20 | ns |
| Prevalence of cavum septum pellucidum (CSP) | 15 | | OR, 1.19 (0.82, 1.72) | 1920 | 1054 | 866 | 0.37 | NA | NA | NA | NA | NA | | 1.19 | ns |
| Basal ganglia N-Acetylaspartate (NAA) | 17 | | SMD, -0.09 | 597 | 325 | 272 | 0.3195 | NA | NA | NA | NA | NA | | 0.85 | ns |
| Brain gray matter (GM) volume, early onset, longitudinal | 4 | | SMD, -0.09 (-0.33, 0.13) | 287 | 140 | 147 | 0.437068 | NA | NA | NA | NA | NA | | 0.85 | ns |
| Parietal N-Acetylaspartate (NAA) | 5 | | SMD, -0.08 | 175 | 92 | 83 | 0.6233 | NA | NA | NA | NA | NA | | 0.86 | ns |
| Dorsolateral prefrontal cortex (DLPFC) choline/creatine (Cho/Cr) | 5 | | SMD, 0.07 (-0.02, 0.15) | NA | - | - | 0.12 | NA | NA | NA | NA | NA | | 1.14 | ns |
| Right hippocomapus cognition and resting-state activation | 10 | | SMD, -0.07 (-0.6, 0.46) | 415 | 190 | 225 | 0.795741 | NA | NA | NA | NA | NA | | 0.88 | ns |
| Frontal choline-containing compounds (Cho) | 10 | | SMD, -0.06 (-0.27, 0.15) | 355 | 195 | 160 | 0.57 | NA | NA | NA | NA | NA | | 0.90 | ns |
| Right caudate gray matter (GM) volume | 10 | | SMD, -0.06 | 565 | 308 | 257 | 0.626069 | NA | NA | NA | NA | NA | | 0.90 | ns |
| Basal ganglia choline/creatine (Cho/Cr) | 4 | | SMD, 0.06 (-0.32, 0.44) | NA | - | - | 0.76 | NA | NA | NA | NA | NA | | 1.11 | ns |
| Left caudate gray matter (GM) volume | 10 | | SMD, 0.06 | 565 | 308 | 257 | 0.660229 | NA | NA | NA | NA | NA | | 1.11 | ns |
| Occipital N-Acetylaspartate (NAA) | 7 | | SMD, 0.06 | 259 | 127 | 132 | 0.6397 | NA | NA | NA | NA | NA | | 1.11 | ns |
| Right temporal resting-state activation | 19 | | SMD, -0.05 (-0.49, 0.38) | 608 | 307 | 301 | 0.821759 | NA | NA | NA | NA | NA | | 0.91 | ns |
| Anterior cingulate cortex (ACC) choline-containing compounds (Cho) | 9 | | SMD, 0.05 (-0.15, 0.24) | 431 | 234 | 197 | 0.64 | NA | NA | NA | NA | NA | | 1.09 | ns |
| Anterior cingulate cortex (ACC) N-acetylaspartate to creatine ratio (NAA/Cr) | 5 | | SMD, 0.04 (-0.64, 0.71) | NA | - | - | 0.91 | NA | NA | NA | NA | NA | | 1.08 | ns |
| Substantia nigra D2 D3 availability | 5 | | SMD, 0.04 (-0.92, 0.99) | 133 | 61 | 72 | 0.934573 | NA | NA | NA | NA | NA | | 1.08 | ns |
| Temporal white matter (WM) volume, medicated | 12 | | SMD, -0.04 | 1008 | 489 | 519 | 0.69 | NA | NA | NA | NA | NA | | 0.93 | ns |
| Hippocampus glutamate (Glu) | 3 | | SMD, 0.03 | 107 | 47 | 60 | 0.92 | NA | NA | NA | NA | NA | | 1.06 | ns |
| Caudate gray matter (GM) volume, medicated | 28 | | SMD, -0.03 (-0.14, 0.07) | 2255 | 1101 | 1154 | 0.5 | NA | NA | NA | NA | NA | | 0.95 | ns |
| Temporal gray matter (GM) volume, early onset, longitudinal | 3 | | SMD, -0.03 (-0.58, 0.53) | 268 | 121 | 147 | 0.923557 | NA | NA | NA | NA | NA | | 0.95 | ns |
| Medial frontal glutamine/glutamate (Glx) | 13 | | SMD, 0.02 (-0.21, 0.24) | 443 | 235 | 208 | 0.861693 | NA | NA | NA | NA | NA | | 1.04 | ns |
| Brain volume, early onset, longitudinal | 3 | | SMD, -0.02 (-0.32, 0.28) | 171 | 74 | 97 | 0.901862 | NA | NA | NA | NA | NA | | 0.97 | ns |
| Brain white matter (WM) volume | 6 | | SMD, -0.01 (-0.29, 0.27) | 460 | 199 | 261 | 0.933414 | NA | NA | NA | NA | NA | | 0.98 | ns |
| Brain volume, longitudinal | 12 | | [incr] | 751 | 470 | 281 | 0.924419 | NA | NA | NA | NA | NA | | NA | ns |
| Brain white matter (WM) volume, longitudinal | 8 | | [decr] | 458 | 228 | 230 | 0.26184 | NA | NA | NA | NA | NA | | NA | ns |
| Caudate gray matter (GM) volume, longitudinal | 13 | | [incr] | 303 | 190 | 113 | 0.31828 | NA | NA | NA | NA | NA | | NA | ns |
| Cerebrospinal fluid volume, lomgitudinal | 3 | | [decr] | 200 | 65 | 135 | 0.184749 | NA | NA | NA | NA | NA | | NA | ns |
| Frontal gray matter (GM) volume, longitudinal | 15 | | [decr] | 1433 | 891 | 542 | 0.511989 | NA | NA | NA | NA | NA | | NA | ns |
| Parietal gray matter (GM) volume, longitudinal | 14 | | [decr] | 1337 | 818 | 519 | 0.823063 | NA | NA | NA | NA | NA | | NA | ns |
| Temporal gray matter (GM) volume, longitudinal | 14 | | [decr] | 1337 | 818 | 519 | 0.507122 | NA | NA | NA | NA | NA | | NA | ns |

CI – confidence interval, CE – class of evidence, [decr] – decreased value in psychosis sample, eOR – equivalent odds ratio, Egger – significant Egger test, ES – effect size, ESB – excess significance bias, FA - fractional anisotropy, FEP – first-episode of psychosis, [incr] – increased value in psychosis sample, k – number of studies for each factor, LS - largest study with significant effect, N – total number of participants, NA – not assessable, ns – not significant, OR – odds ratio, PI – prediction interval, SMD – standardized mean difference.

**sTable 5.** Level of evidence for the association of neuropathological biomarkers and psychotic disorders^98–101^

| **Factor** | **k** | **ES (95% CI)** | **Features used for classification of level of evidence** | | | | | | | | | **eOR** | **Class** |
| --- | --- | --- | --- | --- | --- | --- | --- | --- | --- | --- | --- | --- | --- |
|  |  |  | **N** | **Cases** | **Controls** | **P** | **I^2^** | **PI 95% CI** | **Egger** | **ESB** | **LS** |  |  |
| 25 kDa synaptosomal-associated protein (SNAP-25)-ventral Internal Capsule | 1 | SMD, -259.43 (-332.43, -186.42) | 28 | 15 | 13 | <0.000001 | NA | NA | NA | No | Yes | <0.01 | IV |
| Phosphorylated neurofilament (PNF)-dorsal Internal Capsule | 1 | SMD, -6.41 (-8.36, -4.46) | 28 | 15 | 13 | <0.000001 | NA | NA | NA | No | Yes | <0.01 | IV |
| Phosphorylated neurofilament (PNF)-ventral Internal Capsule | 1 | SMD, -206.89 (-265.11, -148.67) | 28 | 15 | 13 | <0.000001 | NA | NA | NA | No | Yes | <0.01 | IV |
| Phosphorylcholine (N(CH3), 3.21 ppm) Internal Capsule | 1 | SMD, -340 (-435.68, -244.32) | 28 | 15 | 13 | <0.000001 | NA | NA | NA | No | Yes | <0.01 | IV |
| Glia number Magnocellular Lateral geniculate nucleus (thalamus) | 1 | SMD, -3627.4 (-4608.49, -2646.32) | 30 | 15 | 15 | <0.000001 | NA | NA | NA | No | Yes | <0.01 | IV |
| GFAP ratio Claustrum | 1 | SMD, 23.64 (12.19, 35.09) | 12 | 5 | 7 | 0.000052 | NA | NA | NA | No | Yes | >100 | IV |
| Nuclear area of dopamigergic neurons Substantia Nigra | 1 | SMD, 6.98 (4.74, 9.22) | 25 | 12 | 13 | <0.000001 | NA | NA | NA | No | Yes | >100 | IV |
| [125I]BH–substance P (NK1 receptor) Caudate dorso-lateral | 1 | SMD, 3.97 (1.45, 6.49) | 10 | 5 | 5 | 0.002 | NA | NA | NA | No | Yes | >100 | IV |
| [125I]BH–substance P (NK1 receptor) Caudate dorso-median | 1 | SMD, 3.41 (1.15, 5.66) | 10 | 5 | 5 | 0.0031 | NA | NA | NA | No | Yes | >100 | IV |
| [125I]BH–substance P (NK1 receptor) Caudate ventro-lateral | 1 | SMD, 3.32 (1.11, 5.54) | 10 | 5 | 5 | 0.0033 | NA | NA | NA | No | Yes | >100 | IV |
| [125I]BH–substance P (NK1 receptor) Caudate ventro-median | 1 | SMD, 3.31 (1.1, 5.52) | 10 | 5 | 5 | 0.0034 | NA | NA | NA | No | Yes | >100 | IV |
| [125I]BH–substance P (NK1 receptor) Putamen ventro-median | 1 | SMD, 2.82 (0.83, 4.81) | 10 | 5 | 5 | 0.0055 | NA | NA | NA | No | Yes | >100 | IV |
| Neurons total number (106) Mediodorsal thalamic nuclei | 1 | SMD, -2.59 (-3.72, -1.46) | 24 | 12 | 12 | 0.000007 | NA | NA | NA | No | Yes | 0.01 | IV |
| Substance P (f moles/mg protein) anterior and posterior cortical amydaloid nuclei | 1 | SMD, -2.33 (-3.35, -1.32) | 27 | 12 | 15 | 0.000006 | NA | NA | NA | No | Yes | 0.01 | IV |
| [125I]BH–substance P (NK1 receptor) Putamen dorso-median | 1 | SMD, 2.41 (0.59, 4.23) | 10 | 5 | 5 | 0.0095 | NA | NA | NA | No | Yes | 78.91 | IV |
| Neurons total number (106) Nucleus accumbens, ventromedial part | 1 | SMD, -2.28 (-3.35, -1.22) | 24 | 12 | 12 | 0.000027 | NA | NA | NA | No | Yes | 0.02 | IV |
| Astrocytes total number (106) Nucleus accumbens, lateral part | 1 | SMD, -2.31 (-3.38, -1.24) | 24 | 12 | 12 | 0.000023 | NA | NA | NA | No | Yes | 0.02 | IV |
| Capillary cross-sectional area (µm2) Visual Cortex | 1 | SMD, -2.08 (-2.76, -1.39) | 52 | 26 | 26 | <0.000001 | NA | NA | NA | No | Yes | 0.02 | IV |
| Glial cells number (males) CA3 | 1 | SMD, -2.06 (-4.07, -0.06) | 9 | 2 | 7 | 0.044 | NA | NA | NA | No | Yes | 0.02 | IV |
| Substance P (f moles/mg protein) Anterior amygdaloid area | 1 | SMD, -2.15 (-3.13, -1.17) | 27 | 12 | 15 | 0.000017 | NA | NA | NA | No | Yes | 0.02 | IV |
| Cross-sectional area of astrocytic end-feet (µm2) Prefrontal Cortex | 1 | SMD, 2.11 (1.42, 2.79) | 52 | 26 | 26 | <0.000001 | NA | NA | NA | No | Yes | 45.55 | IV |
| [125I]BH–substance P (NK1 receptor) Nucleus accumbens | 1 | SMD, 1.94 (0.3, 3.58) | 10 | 5 | 5 | 0.02 | NA | NA | NA | No | Yes | 33.71 | IV |
| Amyloid Beta (CSF) Aß1–42 | 1 | SMD, -1.95 (-2.84, -1.05) | 31 | 11 | 20 | 0.000022 | NA | NA | NA | No | Yes | 0.03 | IV |
| Neurons total number (106) Nucleus accumbens, lateral part | 1 | SMD, -1.96 (-2.96, -0.96) | 24 | 12 | 12 | 0.00013 | NA | NA | NA | No | Yes | 0.03 | IV |
| [125I]BH–substance P (NK1 receptor) Putamen dorso-lateral | 1 | SMD, 1.91 (0.28, 3.53) | 10 | 5 | 5 | 0.022 | NA | NA | NA | No | Yes | 31.67 | IV |
| [125I]BH–substance P (NK1 receptor) Putamen ventro-lateral | 1 | SMD, 1.84 (0.24, 3.45) | 10 | 5 | 5 | 0.024 | NA | NA | NA | No | Yes | 28.31 | IV |
| Astrocytes total number (106) Mediodorsal thalamic nuclei | 1 | SMD, -1.76 (-2.73, -0.79) | 24 | 12 | 12 | 0.00036 | NA | NA | NA | No | Yes | 0.04 | IV |
| Oligodendroglia total number (106) Mediodorsal thalamic nuclei | 1 | SMD, -1.72 (-2.67, -0.76) | 24 | 12 | 12 | 0.00045 | NA | NA | NA | No | Yes | 0.04 | IV |
| Astrocytes total number (106) Nucleus accumbens, ventromedial part | 1 | SMD, -1.77 (-2.74, -0.8) | 24 | 12 | 12 | 0.00033 | NA | NA | NA | No | Yes | 0.04 | IV |
| Oligodendroglia total number (106) Nucleus accumbens, ventromedial part | 1 | SMD, -1.72 (-2.67, -0.76) | 24 | 12 | 12 | 0.00045 | NA | NA | NA | No | Yes | 0.04 | IV |
| Oligodendroglia total number (106) Nucleus accumbens, lateral part | 1 | SMD, -1.71 (-2.67, -0.75) | 24 | 12 | 12 | 0.00046 | NA | NA | NA | No | Yes | 0.04 | IV |
| GFAP ratio Sup. temporal cortex | 1 | SMD, -1.85 (-3.3, -0.39) | 12 | 5 | 7 | 0.013 | NA | NA | NA | No | Yes | 0.04 | IV |
| S100b Corpus callosum | 1 | SMD, -1.63 (-2.81, -0.45) | 16 | 9 | 7 | 0.0067 | NA | NA | NA | No | Yes | 0.05 | IV |
| [3H]PK11195 binding (gliosis) Superior parietal cortex Parietal cortex | 1 | SMD, -1.64 (-2.68, -0.6) | 20 | 10 | 10 | 0.0021 | NA | NA | NA | No | Yes | 0.05 | IV |
| [3H]PK11195 binding (gliosis) Putamen Extrapyramidal system | 1 | SMD, -1.57 (-2.59, -0.56) | 21 | 12 | 9 | 0.0023 | NA | NA | NA | No | Yes | 0.06 | IV |
| Amyloid Beta (CSF) Aß1–40 | 1 | SMD, -1.47 (-2.31, -0.64) | 31 | 11 | 20 | 0.00052 | NA | NA | NA | No | Yes | 0.07 | IV |
| ALDH1L1 Putamen | 1 | SMD, 1.44 (0.58, 2.31) | 27 | 12 | 15 | 0.0011 | NA | NA | NA | No | Yes | 13.70 | IV |
| pGFAP-Positive Cells Blood vessels Prefrontal Cortex | 1 | OR, 0.08 (0.01, 0.79) | 30 | 15 | 15 | 0.03 | NA | NA | NA | No | Yes | 0.08 | IV |
| Prepotachykinin A (nCi/g) Basal Amigdala | 1 | SMD, -1.4 (-2.22, -0.57) | 29 | 14 | 15 | 0.00088 | NA | NA | NA | No | Yes | 0.08 | IV |
| NG2 Putamen | 1 | SMD, 1.37 (0.52, 2.23) | 27 | 12 | 15 | 0.0017 | NA | NA | NA | No | Yes | 12.03 | IV |
| Prepotachykinin A (nCi/g) Accesory Basal Amygdala | 1 | SMD, -1.32 (-2.14, -0.51) | 29 | 14 | 15 | 0.0014 | NA | NA | NA | No | Yes | 0.09 | IV |
| HLA-B (non smokers) Hippocampus | 1 | SMD, 1.27 (0.55, 2) | 38 | 14 | 24 | 0.00059 | NA | NA | NA | No | Yes | 10.02 | IV |
| [3H]PK11195 binding (gliosis) Visual area 1 Occipital cortex | 1 | SMD, -1.27 (-2.32, -0.21) | 18 | 11 | 7 | 0.019 | NA | NA | NA | No | Yes | 0.10 | IV |
| Amyloid Beta (CSF) Aß1–37 | 1 | SMD, -1.21 (-2.02, -0.41) | 31 | 11 | 20 | 0.0031 | NA | NA | NA | No | Yes | 0.11 | IV |
| Amyloid Beta (CSF) Aß1–38 | 1 | SMD, -1.22 (-2.03, -0.42) | 31 | 11 | 20 | 0.0029 | NA | NA | NA | No | Yes | 0.11 | IV |
| Amyloid Beta (CSF) Aß1–39 | 1 | SMD, -1.22 (-2.03, -0.42) | 31 | 11 | 20 | 0.0029 | NA | NA | NA | No | Yes | 0.11 | IV |
| Myelin-associated glycoprotein (MAG) Mediodorsal thalamic nuclei | 1 | SMD, -1.19 (-2.01, -0.38) | 28 | 14 | 14 | 0.0041 | NA | NA | NA | No | Yes | 0.11 | IV |
| Prepotachykinin A (nCi/g) Lateral amygdala | 1 | SMD, -1.23 (-2.03, -0.43) | 29 | 14 | 15 | 0.0027 | NA | NA | NA | No | Yes | 0.11 | IV |
| Substance P (f moles/mg protein) Basomedial nuclei | 1 | SMD, 1.22 (0.38, 2.05) | 27 | 12 | 15 | 0.0043 | NA | NA | NA | No | Yes | 9.08 | IV |
| [3H]PK11195 binding (gliosis) N. ruber Extrapyramidal system | 1 | SMD, 1.18 (0.16, 2.2) | 18 | 9 | 9 | 0.023 | NA | NA | NA | No | Yes | 8.52 | IV |
| Amyloid Beta (CSF) Aß1–19 | 1 | SMD, -1.18 (-1.98, -0.38) | 31 | 11 | 20 | 0.0037 | NA | NA | NA | No | Yes | 0.12 | IV |
| Amyloid Beta (CSF) Aß11–40 | 1 | SMD, -1.18 (-1.98, -0.38) | 31 | 11 | 20 | 0.0037 | NA | NA | NA | No | Yes | 0.12 | IV |
| Glia number AB24 Anterior cingulate cortex (ACC) | 1 | SMD, -1.16 (-2, -0.32) | 26 | 12 | 14 | 0.007 | NA | NA | NA | No | Yes | 0.12 | IV |
| Galactosylceramidase (GALC) Internal Capsule | 1 | SMD, -1.14 (-1.93, -0.35) | 29 | 14 | 15 | 0.0049 | NA | NA | NA | No | Yes | 0.13 | IV |
| Lactate (3CH3, 1.31 ppm) Internal Capsule | 1 | SMD, -1.1 (-1.91, -0.3) | 28 | 15 | 13 | 0.0073 | NA | NA | NA | No | Yes | 0.14 | IV |
| HLA-A (inflamation) Hippocampus | 1 | SMD, -1.09 (-2.03, -0.15) | 21 | 12 | 9 | 0.023 | NA | NA | NA | No | Yes | 0.14 | IV |
| Nuclear length of dopaminergic neurons Substantia Nigra | 1 | SMD, 1.06 (0.21, 1.9) | 25 | 12 | 13 | 0.014 | NA | NA | NA | No | Yes | 6.80 | IV |
| Amyloid Beta (CSF) Aß1–18 | 1 | SMD, -1.06 (-1.85, -0.28) | 31 | 11 | 20 | 0.0082 | NA | NA | NA | No | Yes | 0.15 | IV |
| Amyloid Beta (CSF) Aß1–33 | 1 | SMD, -1 (-1.79, -0.22) | 31 | 11 | 20 | 0.012 | NA | NA | NA | No | Yes | 0.16 | IV |
| DIO Anterior Cingulate gyrus deep layers (IV-VI) | 1 | SMD, -1.03 (-1.7, -0.35) | 39 | 18 | 21 | 0.0028 | NA | NA | NA | No | Yes | 0.16 | IV |
| Neurons density Mediodorsal thalamic nuclei | 1 | SMD, -1 (-1.86, -0.14) | 24 | 12 | 12 | 0.022 | NA | NA | NA | No | Yes | 0.16 | IV |
| Oligodendrocyte density Subgenual cingulate cortex White matter base | 1 | SMD, -1.01 (-1.83, -0.2) | 29 | 10 | 19 | 0.015 | NA | NA | NA | No | Yes | 0.16 | IV |
| Cross-sectional area of astrocytic end-feet (µm2) Visual Cortex | 1 | SMD, 0.98 (0.41, 1.56) | 52 | 26 | 26 | 0.00085 | NA | NA | NA | No | Yes | 5.95 | IV |
| Glia density Planum temporale | 1 | SMD, -0.97 (-1.73, -0.21) | 30 | 15 | 15 | 0.013 | NA | NA | NA | No | Yes | 0.17 | IV |
| GFAP inmmunoreactivity A9 Layer 4 Prefrontal Cortex | 1 | SMD, 0.95 (0.19, 1.71) | 30 | 15 | 15 | 0.015 | NA | NA | NA | No | Yes | 5.58 | IV |
| Glia density III Anterior cingulate cortex (ACC) right (layer) | 1 | SMD, 0.95 (0.19, 1.71) | 30 | 15 | 15 | 0.015 | NA | NA | NA | No | Yes | 5.57 | IV |
| Glia density III Anterior cingulate cortex (ACC) left (layer) | 1 | SMD, 0.95 (0.19, 1.71) | 30 | 15 | 15 | 0.015 | NA | NA | NA | No | Yes | 5.57 | IV |
| Myelin-associated glycoprotein (MAG) Internal Capsule | 1 | SMD, -0.94 (-1.71, -0.17) | 29 | 14 | 15 | 0.017 | NA | NA | NA | No | Yes | 0.18 | IV |
| Neurons total number (106) Ventral pallidum | 1 | SMD, -0.94 (-1.79, -0.09) | 24 | 12 | 12 | 0.03 | NA | NA | NA | No | Yes | 0.18 | IV |
| Prepotachykinin A (nCi/g) Temporal Cortex | 1 | SMD, -0.94 (-1.72, -0.17) | 29 | 14 | 15 | 0.017 | NA | NA | NA | No | Yes | 0.18 | IV |
| Substance P (f moles/mg protein) Striatal nuclei | 1 | SMD, -0.94 (-1.75, -0.14) | 27 | 12 | 15 | 0.022 | NA | NA | NA | No | Yes | 0.18 | IV |
| Glia density II Anterior cingulate cortex (ACC) right (layer) | 1 | SMD, 0.94 (0.18, 1.7) | 30 | 15 | 15 | 0.016 | NA | NA | NA | No | Yes | 5.47 | IV |
| Glia density II Anterior cingulate cortex (ACC) left (layer) | 1 | SMD, 0.94 (0.18, 1.7) | 30 | 15 | 15 | 0.016 | NA | NA | NA | No | Yes | 5.47 | IV |
| EAAT2 Anterior Cingulate gyrus deep layers (IV-VI) | 1 | SMD, -0.91 (-1.57, -0.24) | 39 | 18 | 21 | 0.0073 | NA | NA | NA | No | Yes | 0.19 | IV |
| Glia density I Anterior cingulate cortex (ACC) right (layer) | 1 | SMD, 0.9 (0.14, 1.65) | 30 | 15 | 15 | 0.02 | NA | NA | NA | No | Yes | 5.09 | IV |
| Glia density I Aanterior cingulate cortex (ACC) left (layer) | 1 | SMD, 0.9 (0.14, 1.65) | 30 | 15 | 15 | 0.02 | NA | NA | NA | No | Yes | 5.09 | IV |
| Amyloid Beta (CSF) Aß1–17 | 1 | SMD, -0.89 (-1.66, -0.12) | 31 | 11 | 20 | 0.024 | NA | NA | NA | No | Yes | 0.20 | IV |
| Amyloid Beta (CSF) Aß1–34 | 1 | SMD, -0.89 (-1.66, -0.12) | 31 | 11 | 20 | 0.024 | NA | NA | NA | No | Yes | 0.20 | IV |
| Glial density Area 3b Ventral PFC | 1 | SMD, 0.88 (0.06, 1.71) | 25 | 12 | 13 | 0.037 | NA | NA | NA | No | Yes | 4.97 | IV |
| Alanine 3CH3, 147 ppm) Internal Capsule | 1 | SMD, -0.85 (-1.63, -0.07) | 28 | 15 | 13 | 0.033 | NA | NA | NA | No | Yes | 0.21 | IV |
| Astrocytes total number (106) Ventral pallidum | 1 | SMD, -0.86 (-1.7, -0.02) | 24 | 12 | 12 | 0.046 | NA | NA | NA | No | Yes | 0.21 | IV |
| Astrocyte density Substantia Nigra | 1 | SMD, -0.85 (-1.68, -0.02) | 25 | 12 | 13 | 0.044 | NA | NA | NA | No | Yes | 0.21 | IV |
| Glia density Vb Anterior cingulate cortex (ACC) right (layer) | 1 | SMD, 0.85 (0.1, 1.6) | 30 | 15 | 15 | 0.026 | NA | NA | NA | No | Yes | 4.68 | IV |
| Glia density Vb Anterior cingulate cortex (ACC) left (layer) | 1 | SMD, 0.85 (0.1, 1.6) | 30 | 15 | 15 | 0.026 | NA | NA | NA | No | Yes | 4.68 | IV |
| DIO Anterior Cingulate gyrus superficial layers (I-III) | 1 | SMD, 0.84 (0.18, 1.5) | 39 | 18 | 21 | 0.012 | NA | NA | NA | No | Yes | 4.61 | IV |
| Astrocyte density Cortical grey matter | 1 | SMD, -0.83 (-1.63, -0.03) | 29 | 10 | 19 | 0.043 | NA | NA | NA | No | Yes | 0.22 | IV |
| Substance P (f moles/mg protein) Medial nuclei | 1 | SMD, 0.83 (0.04, 1.63) | 27 | 12 | 15 | 0.041 | NA | NA | NA | No | Yes | 4.52 | IV |
| Glia density Va Anterior cingulate cortex (ACC) right (layer) | 1 | SMD, 0.82 (0.07, 1.57) | 30 | 15 | 15 | 0.032 | NA | NA | NA | No | Yes | 4.42 | IV |
| Glia density Va Anterior cingulate cortex (ACC) left (layer) | 1 | SMD, 0.82 (0.07, 1.57) | 30 | 15 | 15 | 0.032 | NA | NA | NA | No | Yes | 4.42 | IV |
| AQP4 Anterior Cingulate gyrus deep layers (IV-VI) | 1 | SMD, -0.8 (-1.46, -0.14) | 39 | 18 | 21 | 0.017 | NA | NA | NA | No | Yes | 0.23 | IV |
| GFAP Internal Capsule | 1 | SMD, 0.79 (0.03, 1.56) | 29 | 14 | 15 | 0.041 | NA | NA | NA | No | Yes | 4.23 | IV |
| Glia density VI Anterior cingulate cortex (ACC) right (layer) | 1 | SMD, 0.79 (0.04, 1.54) | 30 | 15 | 15 | 0.038 | NA | NA | NA | No | Yes | 4.19 | IV |
| Glia density VI Anterior cingulate cortex (ACC) left (layer) | 1 | SMD, 0.79 (0.04, 1.54) | 30 | 15 | 15 | 0.038 | NA | NA | NA | No | Yes | 4.19 | IV |
| GFAP inmmunoreactivity A9 Layer 6 Prefrontal Cortex | 1 | SMD, 0.79 (0.04, 1.53) | 30 | 15 | 15 | 0.039 | NA | NA | NA | No | Yes | 4.18 | IV |
| Amyloid Beta (CSF) Aß1–36 | 1 | SMD, -0.78 (-1.54, -0.02) | 31 | 11 | 20 | 0.046 | NA | NA | NA | No | Yes | 0.24 | IV |
| Galactosylceramidase (GALC) Mediodorsal thalamic nuclei | 1 | SMD, -0.79 (-1.57, -0.02) | 28 | 14 | 14 | 0.044 | NA | NA | NA | No | Yes | 0.24 | IV |
| Glutamine synthetase (GS) Anterior Cingulate gyrus deep layers (IV-VI) | 1 | SMD, -0.78 (-1.43, -0.12) | 39 | 18 | 21 | 0.02 | NA | NA | NA | No | Yes | 0.24 | IV |
| GFAP inmmunoreactivity A11/47 Prefrontal Cortex | 1 | SMD, -0.78 (-1.53, -0.04) | 30 | 15 | 15 | 0.04 | NA | NA | NA | No | Yes | 0.24 | IV |
| Glial density Mediodorsal thalamic nuclei | 1 | SMD, -0.78 (-1.54, -0.02) | 29 | 14 | 15 | 0.044 | NA | NA | NA | No | Yes | 0.24 | IV |
| THBS4 Anterior Cingulate gyrus deep layers (IV-VI) | 1 | SMD, -0.76 (-1.42, -0.11) | 39 | 18 | 21 | 0.022 | NA | NA | NA | No | Yes | 0.25 | IV |
| GFAP inmmunoreactivity A9 Layer 3 Prefrontal Cortex | 1 | SMD, 0.75 (0, 1.49) | 30 | 15 | 15 | 0.049 | NA | NA | NA | No | Yes | 3.88 | IV |
| Glutamine synthetase protein expression Superior temporal gyrus | 1 | SMD, -0.75 (-1.32, -0.17) | 50 | 23 | 27 | 0.011 | NA | NA | NA | No | Yes | 0.26 | IV |
| Glutamine synthetase protein expression Anterior cingulate cortex (ACC) | 1 | SMD, -0.73 (-1.31, -0.15) | 50 | 23 | 27 | 0.013 | NA | NA | NA | No | Yes | 0.27 | IV |
| Capillary diameter (µm) Visual Cortex | 1 | SMD, 0.7 (0.14, 1.26) | 52 | 26 | 26 | 0.015 | NA | NA | NA | No | Yes | 3.56 | IV |
| NR1 protein | 3 | SMD, -0.68 (-1.03, -0.33) | 134 | 67 | 67 | 0.00014 | 0% | -2.95, 1.59 | No | No | Yes | 0.29 | IV |
| Phospate-activated glutaminase (GL) Anterior Cingulate gyrus superficial layers (I-III) | 1 | SMD, -0.69 (-1.34, -0.04) | 39 | 18 | 21 | 0.037 | NA | NA | NA | No | Yes | 0.29 | IV |
| GFAP protein expression Anterior cingulate cortex (ACC) | 1 | SMD, -0.69 (-1.27, -0.12) | 50 | 23 | 27 | 0.018 | NA | NA | NA | No | Yes | 0.29 | IV |
| GFAP 43 BA10 | 1 | SMD, 0.67 (0.03, 1.31) | 40 | 20 | 20 | 0.04 | NA | NA | NA | No | Yes | 3.38 | IV |
| GFAP-positive optical density CA1 | 2 | SMD, -0.66 (-1.27, -0.04) | 45 | 21 | 24 | 0.036 | 0% | NA | NA | No | Yes | 0.30 | IV |
| GFAP-positive optical density Dentate gyrus | 2 | SMD, 0.64 (0.03, 1.25) | 45 | 21 | 24 | 0.041 | 0% | NA | NA | No | No | 3.19 | IV |
| GFAP-positive optical density Enthorrinal cortex | 2 | SMD, 0.63 (0.02, 1.24) | 45 | 21 | 24 | 0.044 | 0% | NA | NA | No | Yes | 3.13 | IV |
| NR1 mRNA | 5 | SMD, -0.62 (-1.04, -0.21) | 176 | 94 | 82 | 0.0035 | 39% | -1.78, 0.53 | No | No | Yes | 0.32 | IV |
| Amyloid Beta (post-mortem) neocortex | 1 | SMD, 0.44 (0.09, 0.78) | 150 | 100 | 50 | 0.013 | NA | NA | NA | No | Yes | 2.20 | IV |
| Amyloid Beta (post-mortem) middle frontal gyrus | 2 | SMD, 5.07 (-7.58, 17.72) | 44 | 33 | 11 | 0.43 | 95% | NA | NA | No | Yes | >100 | ns |
| Glial cells number (males) Presubiculum/parasubiculum | 1 | SMD, -1.86 (-3.79, 0.07) | 9 | 2 | 7 | 0.059 | NA | NA | NA | No | Yes | 0.03 | ns |
| Glial cells numbers (males) Enthorrinal cortex | 1 | SMD, -1.61 (-3.45, 0.24) | 9 | 2 | 7 | 0.088 | NA | NA | NA | No | No | 0.05 | ns |
| Glial cells number (males) CA4 | 1 | SMD, -1.34 (-3.11, 0.42) | 9 | 2 | 7 | 0.14 | NA | NA | NA | No | No | 0.09 | ns |
| GFAP ratio Globus pallidus | 1 | SMD, -1.29 (-2.6, 0.01) | 12 | 5 | 7 | 0.052 | NA | NA | NA | No | Yes | 0.10 | ns |
| [3H]PK11195 binding (gliosis) N. subthalamicus Extrapyramidal system | 1 | SMD, 1.22 (-0.22, 2.65) | 10 | 6 | 4 | 0.096 | NA | NA | NA | No | No | 9.07 | ns |
| Glial cells number (females) CA3 | 1 | SMD, 1.14 (-0.1, 2.37) | 15 | 11 | 4 | 0.072 | NA | NA | NA | No | No | 7.84 | ns |
| GFAP ratio Medial thalamus | 1 | SMD, -1.11 (-2.37, 0.16) | 12 | 5 | 7 | 0.086 | NA | NA | NA | No | No | 0.13 | ns |
| GFAP ratio Lateral thalamus | 1 | SMD, -1.1 (-2.37, 0.16) | 12 | 5 | 7 | 0.087 | NA | NA | NA | No | No | 0.14 | ns |
| Glial cells number (males) Prosubiculum/subiculum | 1 | SMD, -1.1 (-2.8, 0.61) | 9 | 2 | 7 | 0.21 | NA | NA | NA | No | No | 0.14 | ns |
| GFAP ratio Cingulate cortex | 1 | SMD, -1.05 (-2.3, 0.21) | 12 | 5 | 7 | 0.1 | NA | NA | NA | No | No | 0.15 | ns |
| [3H]PK11195 binding (gliosis) N. anterior thalami Thalamus | 1 | SMD, -1.02 (-2.21, 0.16) | 13 | 7 | 6 | 0.091 | NA | NA | NA | No | No | 0.16 | ns |
| Preprotachykinin A (cell percentage expressing, µm2) Caudate Nucleus | 1 | SMD, 0.99 (-0.45, 2.44) | 9 | 4 | 5 | 0.18 | NA | NA | NA | No | No | 6.07 | ns |
| [3H]PK11195 binding (gliosis) Premotor area Precentral area | 1 | SMD, -0.97 (-1.96, 0.02) | 18 | 9 | 9 | 0.055 | NA | NA | NA | No | Yes | 0.17 | ns |
| GFAP ratio Dorsal white matter | 1 | SMD, -0.94 (-2.18, 0.29) | 12 | 5 | 7 | 0.13 | NA | NA | NA | No | No | 0.18 | ns |
| Astrocyte density Anterior corpus callosum midline | 1 | SMD, -0.96 (-2.12, 0.21) | 18 | 4 | 14 | 0.11 | NA | NA | NA | No | No | 0.18 | ns |
| Glial cells number (males) CA1/CA2 | 1 | SMD, -0.95 (-2.63, 0.72) | 9 | 2 | 7 | 0.26 | NA | NA | NA | No | No | 0.18 | ns |
| [3H]PK11195 binding (gliosis) Lateral occipitotemporal cortex Temporal cortex | 1 | SMD, -0.9 (-1.93, 0.12) | 17 | 7 | 10 | 0.084 | NA | NA | NA | No | No | 0.19 | ns |
| Neuronal density III Dorsolateral prefrontal cortex (DLPFC) left (layer) | 1 | SMD, -0.83 (-1.75, 0.09) | 20 | 10 | 10 | 0.078 | NA | NA | NA | No | No | 0.22 | ns |
| Myelin-associated glycoprotein (MAG) Putamen | 1 | SMD, -0.79 (-1.58, 0.01) | 27 | 12 | 15 | 0.052 | NA | NA | NA | No | Yes | 0.24 | ns |
| GFAP ratio Corpus callosum | 1 | SMD, -0.78 (-1.98, 0.43) | 12 | 5 | 7 | 0.21 | NA | NA | NA | No | No | 0.24 | ns |
| GFAP ratio Hypothalamus | 1 | SMD, -0.79 (-1.99, 0.42) | 12 | 5 | 7 | 0.2 | NA | NA | NA | No | No | 0.24 | ns |
| Astrocyte density Subgenual cingulate cortex White matter crown | 1 | SMD, -0.78 (-1.58, 0.01) | 29 | 10 | 19 | 0.054 | NA | NA | NA | No | Yes | 0.24 | ns |
| pGFAP-Positive Cells White matter Prefrontal Cortex | 1 | OR, 0.24 (0.05, 1.13) | 30 | 15 | 15 | 0.072 | NA | NA | NA | No | No | 0.24 | ns |
| Pyramidal Neuronal density II Dorsolateral prefrontal cortex (DLPFC) right (layer) | 1 | SMD, 0.78 (-0.13, 1.7) | 20 | 10 | 10 | 0.094 | NA | NA | NA | No | No | 4.14 | ns |
| Glial cells number (females) Prosubiculum/subiculum | 1 | SMD, 0.78 (-0.41, 1.97) | 15 | 11 | 4 | 0.2 | NA | NA | NA | No | No | 4.13 | ns |
| Galactosylceramidase (GALC) Putamen | 1 | SMD, -0.76 (-1.55, 0.03) | 27 | 12 | 15 | 0.06 | NA | NA | NA | No | No | 0.25 | ns |
| GFAP ratio Parietal cortex | 1 | SMD, -0.77 (-1.97, 0.44) | 12 | 5 | 7 | 0.21 | NA | NA | NA | No | No | 0.25 | ns |
| GFAP ratio Caudate | 1 | SMD, -0.75 (-1.96, 0.45) | 12 | 5 | 7 | 0.22 | NA | NA | NA | No | No | 0.25 | ns |
| Astrocyte density Subgenual cingulate cortex White matter base | 1 | SMD, -0.75 (-1.54, 0.05) | 29 | 10 | 19 | 0.065 | NA | NA | NA | No | No | 0.26 | ns |
| [3H]PK11195 binding (gliosis) Substantia nigra Extrapyramidal system | 1 | SMD, 0.74 (-0.2, 1.68) | 19 | 10 | 9 | 0.12 | NA | NA | NA | No | No | 3.81 | ns |
| Neuronal density VI Dorsolateral prefrontal cortex (DLPFC) right (layer) | 1 | SMD, 0.73 (-0.18, 1.64) | 20 | 10 | 10 | 0.12 | NA | NA | NA | No | No | 3.77 | ns |
| HLA-DRA (RT-PCR) BA46 | 1 | SMD, -0.73 (-1.82, 0.37) | 14 | 7 | 7 | 0.19 | NA | NA | NA | No | No | 0.27 | ns |
| Glia cell number Striatum left | 1 | SMD, -0.73 (-1.69, 0.23) | 18 | 9 | 9 | 0.14 | NA | NA | NA | No | No | 0.27 | ns |
| Glia density VI Anterior cingulate cortex (ACC) (Layers) | 1 | SMD, -0.73 (-1.58, 0.12) | 23 | 11 | 12 | 0.093 | NA | NA | NA | No | No | 0.27 | ns |
| Neuronal density I Dorsolateral prefrontal cortex (DLPFC) left (layer) | 1 | SMD, -0.71 (-1.62, 0.2) | 20 | 10 | 10 | 0.12 | NA | NA | NA | No | No | 0.27 | ns |
| [3H]PK11195 binding (gliosis) Somesthetic cortex Parietal cortex | 1 | SMD, -0.72 (-1.67, 0.22) | 19 | 11 | 8 | 0.14 | NA | NA | NA | No | No | 0.27 | ns |
| Amyloid Beta (CSF) Aß1–35 | 1 | SMD, -0.71 (-1.47, 0.05) | 31 | 11 | 20 | 0.067 | NA | NA | NA | No | No | 0.28 | ns |
| Oligodendrocyte density Nucleus Basalis | 1 | SMD, -0.71 (-1.47, 0.05) | 29 | 13 | 16 | 0.066 | NA | NA | NA | No | No | 0.28 | ns |
| Neuronal density V Dorsolateral prefrontal cortex (DLPFC) left (layer) | 1 | SMD, -0.7 (-1.61, 0.21) | 20 | 10 | 10 | 0.13 | NA | NA | NA | No | No | 0.28 | ns |
| ALDH1L1 Anteroventral | 1 | SMD, 0.7 (-0.08, 1.49) | 27 | 12 | 15 | 0.08 | NA | NA | NA | No | No | 3.57 | ns |
| 3H-SP binding sites density (fmol/mg tissue) Superficial layers Temporal cortex | 1 | SMD, 0.69 (-0.06, 1.44) | 29 | 14 | 15 | 0.073 | NA | NA | NA | No | No | 3.48 | ns |
| Neuronal density II Dorsolateral prefrontal cortex (DLPFC) left (layer) | 1 | SMD, -0.69 (-1.6, 0.22) | 20 | 10 | 10 | 0.14 | NA | NA | NA | No | No | 0.29 | ns |
| Pyramidal Neuronal density VI Dorsolateral prefrontal cortex (DLPFC) right (layer) | 1 | SMD, 0.68 (-0.23, 1.58) | 20 | 10 | 10 | 0.14 | NA | NA | NA | No | No | 3.41 | ns |
| Neurons density Planum temporale | 1 | SMD, 0.67 (-0.07, 1.41) | 30 | 15 | 15 | 0.075 | NA | NA | NA | No | No | 3.37 | ns |
| [3H]PK11195 binding (gliosis) N. centralis medialis Thalamus | 1 | SMD, 0.67 (-0.29, 1.62) | 18 | 9 | 9 | 0.17 | NA | NA | NA | No | No | 3.35 | ns |
| GFAP Putamen | 1 | SMD, 0.66 (-0.12, 1.45) | 27 | 12 | 15 | 0.096 | NA | NA | NA | No | No | 3.34 | ns |
| ALDH1L1 Internal Capsule | 1 | SMD, 0.65 (-0.1, 1.4) | 29 | 14 | 15 | 0.088 | NA | NA | NA | No | No | 3.27 | ns |
| S100 Anterior Cingulate gyrus deep layers (IV-VI) | 1 | SMD, -0.64 (-1.29, 0.01) | 39 | 18 | 21 | 0.053 | NA | NA | NA | No | Yes | 0.31 | ns |
| [3H]PK11195 binding (gliosis) Orbital cortex Prefrontal cortex | 1 | SMD, -0.65 (-1.63, 0.33) | 17 | 9 | 8 | 0.2 | NA | NA | NA | No | No | 0.31 | ns |
| Substance P (f moles/mg protein) Pallidum (externa) | 1 | SMD, 0.64 (-0.29, 1.57) | 19 | 10 | 9 | 0.18 | NA | NA | NA | No | No | 3.19 | ns |
| Myelin OL glycoprotein (MOG) Putamen | 1 | SMD, -0.62 (-1.4, 0.16) | 27 | 12 | 15 | 0.12 | NA | NA | NA | No | No | 0.32 | ns |
| MOP dorsal Internal Capsule | 1 | SMD, 0.62 (-0.2, 1.44) | 25 | 15 | 10 | 0.14 | NA | NA | NA | No | No | 3.08 | ns |
| Neuronal density II Dorsolateral prefrontal cortex (DLPFC) right (layer) | 1 | SMD, 0.62 (-0.28, 1.52) | 20 | 10 | 10 | 0.18 | NA | NA | NA | No | No | 3.07 | ns |
| Nucleolar volume of dopaminergic neurons Substantia Nigra | 1 | SMD, 0.62 (-0.19, 1.42) | 25 | 12 | 13 | 0.13 | NA | NA | NA | No | No | 3.06 | ns |
| GFAP ratio Putamen | 1 | SMD, -0.62 (-1.8, 0.57) | 12 | 5 | 7 | 0.31 | NA | NA | NA | No | No | 0.33 | ns |
| Pyramidal Neuronal density III Dorsolateral prefrontal cortex (DLPFC) left (layer) | 1 | SMD, -0.62 (-1.52, 0.28) | 20 | 10 | 10 | 0.18 | NA | NA | NA | No | No | 0.33 | ns |
| Pyramidal Neuronal density VI Dorsolateral prefrontal cortex (DLPFC) left (layer) | 1 | SMD, -0.61 (-1.51, 0.3) | 20 | 10 | 10 | 0.19 | NA | NA | NA | No | No | 0.33 | ns |
| Vimentin-positive astrocyte counts (per mm2) Subiculum | 1 | SMD, -0.61 (-1.4, 0.18) | 26 | 14 | 12 | 0.13 | NA | NA | NA | No | No | 0.33 | ns |
| GFAP-positive optical density CA3 | 2 | SMD, 0.6 (-0.01, 1.21) | 45 | 21 | 24 | 0.055 | 0% | NA | NA | No | No | 2.96 | ns |
| GFAP ratio Ventral white matter | 1 | SMD, -0.59 (-1.77, 0.59) | 12 | 5 | 7 | 0.33 | NA | NA | NA | No | No | 0.34 | ns |
| Oligodendrocyte density Anterior corpus callosum midline | 1 | SMD, -0.59 (-1.73, 0.54) | 18 | 4 | 14 | 0.3 | NA | NA | NA | No | No | 0.34 | ns |
| Glial cells number (females) CA4 | 1 | SMD, -0.6 (-1.77, 0.57) | 15 | 11 | 4 | 0.32 | NA | NA | NA | No | No | 0.34 | ns |
| PHF-1-positive tangles counts (per mm2) Subiculum | 2 | SMD, 0.59 (-0.02, 1.2) | 45 | 21 | 24 | 0.06 | 0% | NA | NA | No | No | 2.89 | ns |
| Myelin-associated glycoprotein (MAG) Anteroventral | 1 | SMD, -0.58 (-1.36, 0.19) | 27 | 12 | 15 | 0.14 | NA | NA | NA | No | No | 0.35 | ns |
| Choline (N(CH3)3, 3.19 ppm) Internal Capsule | 1 | SMD, -0.58 (-1.34, 0.18) | 28 | 15 | 13 | 0.13 | NA | NA | NA | No | No | 0.35 | ns |
| GFAP optical density Cerebellum | 1 | SMD, -0.58 (-1.32, 0.15) | 30 | 15 | 15 | 0.12 | NA | NA | NA | No | No | 0.35 | ns |
| Glia cell number Caudatus/accumbens left | 1 | SMD, -0.58 (-1.53, 0.37) | 18 | 9 | 9 | 0.23 | NA | NA | NA | No | No | 0.35 | ns |
| ALDH1L1 Mediodorsal thalamic nuclei | 1 | SMD, 0.57 (-0.19, 1.33) | 28 | 14 | 14 | 0.14 | NA | NA | NA | No | No | 2.81 | ns |
| EAAT2 Anterior Cingulate gyrus superficial layers (I-III) | 1 | SMD, 0.57 (-0.07, 1.21) | 39 | 18 | 21 | 0.083 | NA | NA | NA | No | No | 2.81 | ns |
| Amyloid Beta (post-mortem) midfrontal cortex - Thioflavine S | 1 | SMD, 0.57 (-0.53, 1.67) | 15 | 10 | 5 | 0.31 | NA | NA | NA | No | No | 2.80 | ns |
| Astrocyte density Nucleus Basalis | 1 | SMD, -0.56 (-1.31, 0.18) | 29 | 13 | 16 | 0.14 | NA | NA | NA | No | No | 0.36 | ns |
| GFAP Mediodorsal thalamic nuclei | 1 | SMD, 0.56 (-0.2, 1.32) | 28 | 14 | 14 | 0.15 | NA | NA | NA | No | No | 2.77 | ns |
| GFAP inmmunoreactivity A9 Layer 2 Prefrontal Cortex | 1 | SMD, 0.56 (-0.17, 1.29) | 30 | 15 | 15 | 0.13 | NA | NA | NA | No | No | 2.75 | ns |
| GFAP ratio Hippocampus | 1 | SMD, -0.55 (-1.73, 0.63) | 12 | 5 | 7 | 0.36 | NA | NA | NA | No | No | 0.37 | ns |
| GFAP-positive optical density Subiculum | 2 | SMD, 0.55 (-0.19, 1.29) | 45 | 21 | 24 | 0.15 | 31% | NA | NA | No | Yes | 2.69 | ns |
| 3H-SP binding sites density (fmol/mg tissue) Total cortical layers Temporal cortex | 1 | SMD, 0.54 (-0.21, 1.28) | 29 | 14 | 15 | 0.16 | NA | NA | NA | No | No | 2.65 | ns |
| GFAP inmmunoreactivity A9 Layer 1 Prefrontal Cortex | 1 | SMD, 0.54 (-0.19, 1.27) | 30 | 15 | 15 | 0.15 | NA | NA | NA | No | No | 2.64 | ns |
| Glia density (cells/mm3×103) III Insular cortex (layer) | 1 | SMD, 0.53 (-0.2, 1.26) | 30 | 15 | 15 | 0.15 | NA | NA | NA | No | No | 2.64 | ns |
| [3H]PK11195 binding (gliosis) N. lateralis posterior Thalamus | 1 | SMD, -0.54 (-1.43, 0.36) | 20 | 10 | 10 | 0.24 | NA | NA | NA | No | No | 0.38 | ns |
| Glial density Area sg24 Ventral PFC | 1 | SMD, -0.54 (-1.39, 0.32) | 22 | 11 | 11 | 0.22 | NA | NA | NA | No | No | 0.38 | ns |
| Substance P (f moles/mg protein) Central nuclei | 1 | SMD, -0.53 (-1.31, 0.24) | 27 | 12 | 15 | 0.18 | NA | NA | NA | No | No | 0.38 | ns |
| Substance P (f moles/mg protein) Lateral nuclei | 1 | SMD, -0.53 (-1.3, 0.25) | 27 | 12 | 15 | 0.18 | NA | NA | NA | No | No | 0.38 | ns |
| Substance P (f moles/mg protein) Putamen | 1 | SMD, 0.53 (-0.41, 1.48) | 18 | 9 | 9 | 0.27 | NA | NA | NA | No | No | 2.62 | ns |
| Amyloid Beta (post-mortem) frontal | 2 | SMD, -0.52 (-2.05, 1.01) | 24 | 17 | 7 | 0.51 | 61% | NA | NA | No | No | 0.39 | ns |
| Hypocellular gap density of cells Subventricular zone | 1 | SMD, -0.52 (-1.25, 0.21) | 30 | 15 | 15 | 0.16 | NA | NA | NA | No | No | 0.39 | ns |
| Neuronal density IV Dorsolateral prefrontal cortex (DLPFC) left (layer) | 1 | SMD, -0.52 (-1.42, 0.37) | 20 | 10 | 10 | 0.25 | NA | NA | NA | No | No | 0.39 | ns |
| Neuronal density VI Dorsolateral prefrontal cortex (DLPFC) left (layer) | 1 | SMD, -0.52 (-1.41, 0.38) | 20 | 10 | 10 | 0.26 | NA | NA | NA | No | No | 0.39 | ns |
| Pyramidal Neuronal density III Dorsolateral prefrontal cortex (DLPFC) right (layer) | 1 | SMD, 0.51 (-0.39, 1.4) | 20 | 10 | 10 | 0.27 | NA | NA | NA | No | No | 2.50 | ns |
| THBS4 Anterior Cingulate gyrus superficial layers (I-III) | 1 | SMD, -0.51 (-1.15, 0.13) | 39 | 18 | 21 | 0.12 | NA | NA | NA | No | No | 0.40 | ns |
| Astrocytic cytoplams area Hippocampus | 1 | SMD, -0.5 (-1.18, 0.17) | 35 | 19 | 16 | 0.15 | NA | NA | NA | No | No | 0.40 | ns |
| Glial cells number (females) CA1/CA2 | 1 | SMD, -0.5 (-1.67, 0.66) | 15 | 11 | 4 | 0.4 | NA | NA | NA | No | No | 0.40 | ns |
| GFAP inmmunoreactivity A9 Layer 5 Prefrontal Cortex | 1 | SMD, 0.5 (-0.23, 1.23) | 30 | 15 | 15 | 0.18 | NA | NA | NA | No | No | 2.49 | ns |
| Pyramidal Neuronal density I Dorsolateral prefrontal cortex (DLPFC) right (layer) | 1 | SMD, 0.5 (-0.4, 1.39) | 20 | 10 | 10 | 0.28 | NA | NA | NA | No | No | 2.46 | ns |
| N-acetylaspartate (acetyl moiety, 2CH3, 2.01 ppm) Internal Capsule | 1 | SMD, -0.49 (-1.25, 0.27) | 28 | 15 | 13 | 0.2 | NA | NA | NA | No | No | 0.41 | ns |
| Glial cells number (females) Enthorrinal cortex | 1 | SMD, 0.49 (-0.67, 1.65) | 15 | 11 | 4 | 0.41 | NA | NA | NA | No | No | 2.43 | ns |
| Substance P (f moles/mg protein) Caudate | 1 | SMD, 0.49 (-0.43, 1.41) | 19 | 9 | 10 | 0.3 | NA | NA | NA | No | No | 2.42 | ns |
| DIO Anterior Cingulate gyrus White Matter | 1 | SMD, -0.47 (-1.11, 0.16) | 39 | 18 | 21 | 0.15 | NA | NA | NA | No | No | 0.42 | ns |
| Oligodendrocyte density Cortical grey matter | 1 | SMD, -0.48 (-1.26, 0.29) | 29 | 10 | 19 | 0.22 | NA | NA | NA | No | No | 0.42 | ns |
| Pyramidal Neuronal density IV Dorsolateral prefrontal cortex (DLPFC) left (layer) | 1 | SMD, -0.47 (-1.36, 0.42) | 20 | 10 | 10 | 0.3 | NA | NA | NA | No | No | 0.42 | ns |
| [3H]PK11195 binding (gliosis) Pallidum externa Extrapyramidal system | 1 | SMD, -0.48 (-1.45, 0.49) | 17 | 9 | 8 | 0.33 | NA | NA | NA | No | No | 0.42 | ns |
| Microglial cell-density (cells/mm3) Hippocampus-L | 2 | SMD, 0.47 (-0.26, 1.2) | 32 | 20 | 12 | 0.2 | 0% | NA | NA | No | No | 2.36 | ns |
| GFAP ratio Mid. temporal cortex | 1 | SMD, -0.46 (-1.63, 0.71) | 12 | 5 | 7 | 0.44 | NA | NA | NA | No | No | 0.43 | ns |
| Glia cell number Caudatus/accumbens right | 1 | SMD, -0.46 (-1.4, 0.48) | 18 | 9 | 9 | 0.34 | NA | NA | NA | No | No | 0.43 | ns |
| Glial cells numbers CA1 Hippocampus | 1 | SMD, -0.47 (-1.69, 0.75) | 12 | 4 | 8 | 0.45 | NA | NA | NA | No | No | 0.43 | ns |
| NG2 Internal Capsule | 1 | SMD, 0.46 (-0.28, 1.2) | 29 | 14 | 15 | 0.22 | NA | NA | NA | No | No | 2.31 | ns |
| Myo-inositol (2CH, 4.05 ppm) Internal Capsule | 1 | SMD, -0.45 (-1.21, 0.3) | 28 | 15 | 13 | 0.24 | NA | NA | NA | No | No | 0.44 | ns |
| GFAP-positive astrocyte counts (per mm2) CA3 | 2 | SMD, -0.43 (-1.1, 0.24) | 45 | 21 | 24 | 0.21 | 17% | NA | NA | No | No | 0.46 | ns |
| Microglial cell-density (cells/mm3) Hi-L | 1 | SMD, 0.42 (-0.28, 1.12) | 32 | 16 | 16 | 0.24 | NA | NA | NA | No | No | 2.15 | ns |
| GFAP-positive cells nuclear diameter Enthorrinal cortex | 2 | SMD, 0.42 (-0.18, 1.02) | 44 | 21 | 23 | 0.17 | 0% | NA | NA | No | No | 2.13 | ns |
| Amyloid Beta (CSF) Aß10–40 | 1 | SMD, -0.41 (-1.16, 0.33) | 31 | 11 | 20 | 0.28 | NA | NA | NA | No | No | 0.47 | ns |
| ALDH1L1 Anterior Cingulate gyrus deep layers (IV-VI) | 1 | SMD, -0.42 (-1.06, 0.22) | 39 | 18 | 21 | 0.2 | NA | NA | NA | No | No | 0.47 | ns |
| GFAP protein expression Superior temporal gyrus | 1 | SMD, -0.41 (-0.97, 0.15) | 50 | 23 | 27 | 0.15 | NA | NA | NA | No | No | 0.47 | ns |
| Glia density V Anterior cingulate cortex (ACC) (Layers) | 1 | SMD, -0.42 (-1.25, 0.41) | 23 | 11 | 12 | 0.32 | NA | NA | NA | No | No | 0.47 | ns |
| GFAP-positive optical density Midfrontal cortex | 2 | SMD, 0.41 (-0.19, 1.01) | 45 | 21 | 24 | 0.18 | 0% | NA | NA | No | No | 2.10 | ns |
| Microglial cell-density (cells/mm3) DLPC-R | 1 | SMD, -0.4 (-1.1, 0.3) | 32 | 16 | 16 | 0.26 | NA | NA | NA | No | No | 0.48 | ns |
| [3H]PK11195 binding (gliosis) Caudate Extrapyramidal system | 1 | SMD, -0.41 (-1.28, 0.46) | 21 | 11 | 10 | 0.35 | NA | NA | NA | No | No | 0.48 | ns |
| Glial cells number (females) Presubiculum/parasubiculum | 1 | SMD, 0.4 (-0.75, 1.56) | 15 | 11 | 4 | 0.49 | NA | NA | NA | No | No | 2.08 | ns |
| GFAP-positive astrocyte counts (per mm2) Dentate gyrus | 2 | SMD, 0.4 (-0.2, 1) | 45 | 21 | 24 | 0.19 | 0% | NA | NA | No | No | 2.07 | ns |
| Glutamine synthetase (GS) Anterior Cingulate gyrus superficial layers (I-III) | 1 | SMD, 0.4 (-0.24, 1.04) | 39 | 18 | 21 | 0.22 | NA | NA | NA | No | No | 2.07 | ns |
| Bcl-2 BA9 | 1 | SMD, 0.4 (-0.23, 1.02) | 40 | 20 | 20 | 0.21 | NA | NA | NA | No | No | 2.05 | ns |
| Astrocytic nucleus area Hippocampus | 1 | SMD, -0.39 (-1.06, 0.28) | 35 | 19 | 16 | 0.25 | NA | NA | NA | No | No | 0.49 | ns |
| GFAP-positive optical density Visual cortex | 2 | SMD, 0.39 (-0.21, 0.99) | 45 | 21 | 24 | 0.2 | 0% | NA | NA | No | No | 2.04 | ns |
| Glial nuclear size Mediodorsal thalamic nuclei | 1 | SMD, 0.39 (-0.34, 1.13) | 29 | 14 | 15 | 0.29 | NA | NA | NA | No | No | 2.04 | ns |
| Microglial cell-density (cells/mm3) Dorsolateral prefrontal cortex (DLPFC)-L | 2 | SMD, 0.39 (-1.08, 1.86) | 32 | 20 | 12 | 0.61 | 74% | NA | NA | No | No | 2.01 | ns |
| Amyloid Beta (post-mortem) hippocampal | 2 | SMD, -0.38 (-2.22, 1.46) | 24 | 17 | 7 | 0.68 | 73% | NA | NA | No | No | 0.50 | ns |
| Acetate (2CH3, 1.90 ppm) Internal Capsule | 1 | SMD, -0.39 (-1.14, 0.36) | 28 | 15 | 13 | 0.31 | NA | NA | NA | No | No | 0.50 | ns |
| GFAP ratio Lateral amygdala | 1 | SMD, -0.38 (-1.54, 0.79) | 12 | 5 | 7 | 0.53 | NA | NA | NA | No | No | 0.50 | ns |
| GFAP-positive optical density Orbital frontal cortex | 2 | SMD, 0.38 (-0.22, 0.98) | 45 | 21 | 24 | 0.22 | 0% | NA | NA | No | No | 1.99 | ns |
| GFAP Anteroventral | 1 | SMD, 0.38 (-0.39, 1.15) | 27 | 12 | 15 | 0.33 | NA | NA | NA | No | No | 1.99 | ns |
| 3H-SP binding sites density (fmol/mg tissue) Deep layers Temporal cortex | 1 | SMD, 0.38 (-0.36, 1.11) | 29 | 14 | 15 | 0.32 | NA | NA | NA | No | No | 1.98 | ns |
| Substance P (f moles/mg protein) Pallidum (interna) | 1 | SMD, 0.38 (-0.62, 1.38) | 16 | 9 | 7 | 0.46 | NA | NA | NA | No | No | 1.98 | ns |
| GFAP-positive astrocyte counts (per mm2) Midfrontal cortex | 2 | SMD, 0.37 (-0.23, 0.97) | 45 | 21 | 24 | 0.23 | 0% | NA | NA | No | No | 1.95 | ns |
| Bcl-2 BA46 | 1 | SMD, -0.37 (-0.99, 0.26) | 40 | 20 | 20 | 0.25 | NA | NA | NA | No | No | 0.52 | ns |
| Glia total number Mediodorsal thalamic nuclei | 1 | SMD, -0.36 (-1.09, 0.38) | 29 | 14 | 15 | 0.34 | NA | NA | NA | No | No | 0.52 | ns |
| Bcl-2 BA40 | 1 | SMD, 0.35 (-0.28, 0.97) | 40 | 20 | 20 | 0.27 | NA | NA | NA | No | No | 1.89 | ns |
| Creatine/phosphocreatine (N(CH3), 3.03 ppm) Internal Capsule | 1 | SMD, -0.35 (-1.09, 0.4) | 28 | 15 | 13 | 0.37 | NA | NA | NA | No | No | 0.53 | ns |
| GFAP ratio Medial amygdala | 1 | SMD, -0.35 (-1.51, 0.81) | 12 | 5 | 7 | 0.56 | NA | NA | NA | No | No | 0.53 | ns |
| Glia cell number Striatum right | 1 | SMD, -0.35 (-1.28, 0.58) | 18 | 9 | 9 | 0.46 | NA | NA | NA | No | No | 0.53 | ns |
| [3H]PK11195 binding (gliosis) N. lateralis anterior Thalamus | 1 | SMD, -0.35 (-1.35, 0.65) | 16 | 7 | 9 | 0.49 | NA | NA | NA | No | No | 0.53 | ns |
| Preprotachykinin A (grain densities, grain/µm2) Caudate Nucleus | 1 | SMD, -0.35 (-1.68, 0.99) | 9 | 4 | 5 | 0.61 | NA | NA | NA | No | No | 0.53 | ns |
| S100 Anterior Cingulate gyrus superficial layers (I-III) | 1 | SMD, -0.34 (-0.97, 0.3) | 39 | 18 | 21 | 0.3 | NA | NA | NA | No | No | 0.54 | ns |
| Pyramidal Neuronal density V Dorsolateral prefrontal cortex (DLPFC) left (layer) | 1 | SMD, -0.34 (-1.22, 0.54) | 20 | 10 | 10 | 0.45 | NA | NA | NA | No | No | 0.54 | ns |
| Glia density I Anterior cingulate cortex (ACC) (Layers) | 1 | SMD, 0.33 (-0.5, 1.15) | 23 | 11 | 12 | 0.43 | NA | NA | NA | No | No | 1.82 | ns |
| Neuronal density III Dorsolateral prefrontal cortex (DLPFC) right (layer) | 1 | SMD, 0.33 (-0.55, 1.21) | 20 | 10 | 10 | 0.46 | NA | NA | NA | No | No | 1.82 | ns |
| PHF-1-positive tangles counts (per mm2) CA3 | 1 | SMD, -0.33 (-1.11, 0.45) | 26 | 14 | 12 | 0.4 | NA | NA | NA | No | No | 0.55 | ns |
| [3H]PK11195 binding (gliosis) Medial frontal cortex Prefrontal cortex | 1 | SMD, 0.32 (-0.63, 1.28) | 18 | 11 | 7 | 0.51 | NA | NA | NA | No | No | 1.80 | ns |
| Neurons total number (106) Basolateral nucleus of amygdala | 1 | SMD, 0.32 (-0.49, 1.13) | 24 | 12 | 12 | 0.44 | NA | NA | NA | No | No | 1.79 | ns |
| S100 Anterior Cingulate gyrus White Matter | 1 | SMD, -0.32 (-0.95, 0.32) | 39 | 18 | 21 | 0.33 | NA | NA | NA | No | No | 0.56 | ns |
| Capillary diameter (µm) Prefrontal Cortex | 1 | SMD, -0.32 (-0.87, 0.23) | 52 | 26 | 26 | 0.25 | NA | NA | NA | No | No | 0.56 | ns |
| Substance P (f moles/mg protein) Basolateral nuclei | 1 | SMD, 0.32 (-0.45, 1.08) | 27 | 12 | 15 | 0.41 | NA | NA | NA | No | No | 1.78 | ns |
| Glutamine synthetase protein expression Primary visual cortex | 1 | SMD, 0.31 (-0.25, 0.87) | 50 | 23 | 27 | 0.27 | NA | NA | NA | No | No | 1.77 | ns |
| Galactosylceramidase (GALC) Anteroventral | 1 | SMD, -0.31 (-1.07, 0.45) | 27 | 12 | 15 | 0.43 | NA | NA | NA | No | No | 0.57 | ns |
| Glutamine synthetase (GS) Anterior Cingulate gyrus White Matter | 1 | SMD, -0.31 (-0.94, 0.33) | 39 | 18 | 21 | 0.34 | NA | NA | NA | No | No | 0.57 | ns |
| Amyloid Beta (post-mortem) hippocampus | 1 | SMD, 0.31 (-0.52, 1.14) | 23 | 10 | 13 | 0.47 | NA | NA | NA | No | No | 1.75 | ns |
| Microglial cell-density (cells/mm3) Hippocampus-R | 2 | SMD, 0.31 (-0.41, 1.03) | 32 | 20 | 12 | 0.4 | 0% | NA | NA | No | No | 1.75 | ns |
| Glial number Area 3b Ventral PFC | 1 | SMD, 0.3 (-0.49, 1.09) | 25 | 12 | 13 | 0.46 | NA | NA | NA | No | No | 1.73 | ns |
| Oligodendroglia total number (106) Basolateral nucleus of amygdala | 1 | SMD, -0.3 (-1.11, 0.5) | 24 | 12 | 12 | 0.46 | NA | NA | NA | No | No | 0.58 | ns |
| GFAP protein expression Dorsolateral prefrontal cortex (DLPFC) | 1 | SMD, -0.3 (-0.86, 0.26) | 50 | 23 | 27 | 0.29 | NA | NA | NA | No | No | 0.58 | ns |
| NG2 Anteroventral | 1 | SMD, 0.3 (-0.47, 1.06) | 27 | 12 | 15 | 0.45 | NA | NA | NA | No | No | 1.71 | ns |
| Amyloid Beta (post-mortem) orbitofrontal - 2332 | 1 | SMD, 0.29 (-0.37, 0.96) | 37 | 23 | 14 | 0.39 | NA | NA | NA | No | No | 1.70 | ns |
| Pyramidal Neuronal density IV Dorsolateral prefrontal cortex (DLPFC) right (layer) | 1 | SMD, 0.29 (-0.59, 1.18) | 20 | 10 | 10 | 0.51 | NA | NA | NA | No | No | 1.70 | ns |
| GFAP-positive astrocyte counts (per mm2) Subiculum | 2 | SMD, 0.29 (-1.05, 1.63) | 45 | 21 | 24 | 0.68 | 78% | NA | NA | No | Yes | 1.68 | ns |
| PHF-1-positive tangles counts (per mm2) Enthorrinal cortex | 2 | SMD, 0.29 (-0.31, 0.89) | 45 | 21 | 24 | 0.35 | 0% | NA | NA | No | No | 1.68 | ns |
| Astrocytic-corrected density Enthorrinal cortex | 2 | SMD, 0.28 (-0.71, 1.27) | 44 | 21 | 23 | 0.58 | 58% | NA | NA | No | No | 1.67 | ns |
| GFAP 43 BA9 | 1 | SMD, 0.28 (-0.35, 0.9) | 40 | 20 | 20 | 0.38 | NA | NA | NA | No | No | 1.66 | ns |
| Preprotachykinin A (cell area expressing, µm2) Caudate Nucleus | 1 | SMD, 0.28 (-1.05, 1.61) | 9 | 4 | 5 | 0.68 | NA | NA | NA | No | No | 1.66 | ns |
| EAAT2 Anterior Cingulate gyrus White Matter | 1 | SMD, -0.27 (-0.9, 0.36) | 39 | 18 | 21 | 0.41 | NA | NA | NA | No | No | 0.61 | ns |
| Phospate-activated glutaminase (GL) Anterior Cingulate gyrus White Matter | 1 | SMD, -0.27 (-0.9, 0.36) | 39 | 18 | 21 | 0.4 | NA | NA | NA | No | No | 0.61 | ns |
| Glia cell number Putamen right | 1 | SMD, -0.27 (-1.2, 0.66) | 18 | 9 | 9 | 0.56 | NA | NA | NA | No | No | 0.61 | ns |
| [3H]PK11195 binding (gliosis) N. dorsomedialis Thalamus | 1 | SMD, -0.27 (-1.2, 0.66) | 18 | 9 | 9 | 0.57 | NA | NA | NA | No | No | 0.61 | ns |
| Glia density III Anterior cingulate cortex (ACC) (Layers) | 1 | SMD, -0.26 (-1.09, 0.56) | 23 | 11 | 12 | 0.53 | NA | NA | NA | No | No | 0.62 | ns |
| VIM Anterior Cingulate gyrus White Matter | 1 | SMD, 0.26 (-0.37, 0.89) | 39 | 18 | 21 | 0.42 | NA | NA | NA | No | No | 1.61 | ns |
| GFAP protein expression Primary visual cortex | 1 | SMD, -0.25 (-0.81, 0.31) | 50 | 23 | 27 | 0.38 | NA | NA | NA | No | No | 0.63 | ns |
| GFAP ventral Internal Capsule | 1 | SMD, 0.25 (-0.5, 1) | 28 | 15 | 13 | 0.51 | NA | NA | NA | No | No | 1.58 | ns |
| Glial cells numbers CA4 Hippocampus | 1 | SMD, -0.25 (-1.45, 0.96) | 12 | 4 | 8 | 0.69 | NA | NA | NA | No | No | 0.64 | ns |
| [3H]PK11195 binding (gliosis) Pallidum interna Extrapyramidal system | 1 | SMD, -0.25 (-1.23, 0.74) | 16 | 8 | 8 | 0.62 | NA | NA | NA | No | No | 0.64 | ns |
| Amyloid Beta (post-mortem) calcarine - 2332 | 1 | SMD, 0.24 (-0.42, 0.91) | 37 | 23 | 14 | 0.47 | NA | NA | NA | No | No | 1.56 | ns |
| PHF-1-positive tangles counts (per mm2) CA1 | 2 | SMD, 0.25 (-0.35, 0.84) | 45 | 21 | 24 | 0.42 | 0% | NA | NA | No | No | 1.56 | ns |
| Pyramidal Neuronal density I Dorsolateral prefrontal cortex (DLPFC) left (layer) | 1 | SMD, 0.24 (-0.64, 1.12) | 20 | 10 | 10 | 0.6 | NA | NA | NA | No | No | 1.54 | ns |
| GFAP Anterior Cingulate gyrus superficial layers (I-III) | 1 | SMD, -0.23 (-0.87, 0.4) | 39 | 18 | 21 | 0.47 | NA | NA | NA | No | No | 0.65 | ns |
| ALDH1L1 Anterior Cingulate gyrus White Matter | 1 | SMD, -0.24 (-0.87, 0.39) | 39 | 18 | 21 | 0.46 | NA | NA | NA | No | No | 0.65 | ns |
| Amyloid Beta (post-mortem) midfrontal - 2332 | 1 | SMD, 0.23 (-0.44, 0.9) | 37 | 23 | 14 | 0.5 | NA | NA | NA | No | No | 1.52 | ns |
| Brain weight SZ-female | 15 | SMD, -0.23 (-0.5, 0.05) | 503 | 231 | 272 | 0.1 | 52% | -1.01, 0.55 | No | Yes | Yes | 0.66 | ns |
| Pyramidal Neuronal density V Dorsolateral prefrontal cortex (DLPFC) right (layer) | 1 | SMD, -0.23 (-1.11, 0.65) | 20 | 10 | 10 | 0.6 | NA | NA | NA | No | No | 0.66 | ns |
| GFAP Anterior Cingulate gyrus White Matter | 1 | SMD, 0.23 (-0.4, 0.86) | 39 | 18 | 21 | 0.48 | NA | NA | NA | No | No | 1.51 | ns |
| Glia density IV Anterior cingulate cortex (ACC) (Layers) | 1 | SMD, -0.22 (-1.04, 0.6) | 23 | 11 | 12 | 0.6 | NA | NA | NA | No | No | 0.67 | ns |
| ALDH1L1 Anterior Cingulate gyrus superficial layers (I-III) | 1 | SMD, 0.21 (-0.42, 0.85) | 39 | 18 | 21 | 0.51 | NA | NA | NA | No | No | 1.47 | ns |
| Glia nuclear size (µm3) III Insular cortex (layer) | 1 | SMD, 0.21 (-0.51, 0.93) | 30 | 15 | 15 | 0.57 | NA | NA | NA | No | No | 1.46 | ns |
| NG2 Mediodorsal thalamic nuclei | 1 | SMD, 0.21 (-0.54, 0.95) | 28 | 14 | 14 | 0.59 | NA | NA | NA | No | No | 1.45 | ns |
| Microglial cell-density (cells/mm3) Hi-R | 1 | SMD, 0.2 (-0.49, 0.9) | 32 | 16 | 16 | 0.57 | NA | NA | NA | No | No | 1.45 | ns |
| pGFAP-Positive Cells Layer VI Prefrontal Cortex | 1 | OR, 0.69 (0.12, 3.79) | 30 | 15 | 15 | 0.67 | NA | NA | NA | No | No | 0.69 | ns |
| Glia nuclear size (µm3) II Insular cortex (layer) | 1 | SMD, 0.2 (-0.52, 0.92) | 30 | 15 | 15 | 0.58 | NA | NA | NA | No | No | 1.44 | ns |
| Glia number AB32 Anterior cingulate cortex (ACC) | 1 | SMD, -0.2 (-0.97, 0.58) | 26 | 12 | 14 | 0.62 | NA | NA | NA | No | No | 0.70 | ns |
| GFAP dorsal Internal Capsule | 1 | SMD, 0.19 (-0.55, 0.94) | 28 | 15 | 13 | 0.62 | NA | NA | NA | No | No | 1.41 | ns |
| Glutamine synthetase protein expression Hippocampus | 1 | SMD, 0.19 (-0.37, 0.75) | 50 | 23 | 27 | 0.5 | NA | NA | NA | No | No | 1.41 | ns |
| 3H-SP binding sites density (fmol/mg tissue) Accessory basal amygdala | 1 | SMD, 0.19 (-0.54, 0.92) | 29 | 14 | 15 | 0.61 | NA | NA | NA | No | No | 1.41 | ns |
| Microglial cell-density (cells/mm3) ACC-L | 3 | SMD, 0.19 (-0.32, 0.69) | 64 | 36 | 28 | 0.47 | 0% | -3.08, 3.45 | No | No | No | 1.40 | ns |
| Microglial cell-density (cells/mm3) MD-L | 2 | SMD, 0.18 (-0.97, 1.33) | 32 | 20 | 12 | 0.76 | 59% | NA | NA | No | No | 1.38 | ns |
| 25 kDa synaptosomal-associated protein (SNAP-25)-dorsal Internal Capsule | 1 | SMD, -0.17 (-0.92, 0.57) | 28 | 15 | 13 | 0.65 | NA | NA | NA | No | No | 0.73 | ns |
| Neuronal density V Dorsolateral prefrontal cortex (DLPFC) right (layer) | 1 | SMD, -0.17 (-1.05, 0.7) | 20 | 10 | 10 | 0.7 | NA | NA | NA | No | No | 0.73 | ns |
| [3H]PK11195 binding (gliosis) Visual area 2 and 3 Occipital cortex | 1 | SMD, -0.17 (-1.16, 0.82) | 16 | 7 | 9 | 0.74 | NA | NA | NA | No | No | 0.73 | ns |
| Glutamine synthetase protein expression Dorsolateral prefrontal cortex (DLPFC) | 1 | SMD, 0.17 (-0.39, 0.73) | 50 | 23 | 27 | 0.55 | NA | NA | NA | No | No | 1.36 | ns |
| Glia density II anterior cingulate cortex (ACC) (Layers) | 1 | SMD, -0.16 (-0.98, 0.66) | 23 | 11 | 12 | 0.7 | NA | NA | NA | No | No | 0.74 | ns |
| [3H]PK11195 binding (gliosis) Medial and inferior temporal cortex Temporal cortex | 1 | SMD, -0.17 (-1.07, 0.74) | 19 | 9 | 10 | 0.72 | NA | NA | NA | No | No | 0.74 | ns |
| Glia density (cells/mm3×103) II Insular cortex (layer) | 1 | SMD, -0.16 (-0.88, 0.55) | 30 | 15 | 15 | 0.65 | NA | NA | NA | No | No | 0.74 | ns |
| ß-Actin proteins optical density Cerebellum | 1 | SMD, -0.16 (-0.88, 0.56) | 30 | 15 | 15 | 0.66 | NA | NA | NA | No | No | 0.75 | ns |
| Neurons size Planum temporale | 1 | SMD, -0.16 (-0.88, 0.56) | 30 | 15 | 15 | 0.67 | NA | NA | NA | No | No | 0.75 | ns |
| Vimentin-positive astrocyte counts (per mm2) Enthorrinal cortex | 1 | SMD, 0.16 (-0.62, 0.93) | 26 | 14 | 12 | 0.69 | NA | NA | NA | No | No | 1.33 | ns |
| Amyloid Beta (post-mortem) CA1 - 2332 | 1 | SMD, 0.15 (-0.51, 0.82) | 37 | 23 | 14 | 0.65 | NA | NA | NA | No | No | 1.32 | ns |
| [3H]PK11195 binding (gliosis) Orbitofrontal cortex Prefrontal cortex | 1 | SMD, 0.15 (-0.78, 1.09) | 19 | 12 | 7 | 0.75 | NA | NA | NA | No | No | 1.32 | ns |
| Succinate (2CH2, 2.40 ppm) Internal Capsule | 1 | SMD, -0.15 (-0.9, 0.59) | 28 | 15 | 13 | 0.68 | NA | NA | NA | No | No | 0.76 | ns |
| AQP4 Anterior Cingulate gyrus White Matter | 1 | SMD, -0.15 (-0.79, 0.48) | 39 | 18 | 21 | 0.63 | NA | NA | NA | No | No | 0.76 | ns |
| Glial cells numbers CA3 Hippocampus | 1 | SMD, -0.15 (-1.35, 1.05) | 12 | 4 | 8 | 0.81 | NA | NA | NA | No | No | 0.76 | ns |
| GFAP Anterior Cingulate gyrus deep layers (IV-VI) | 1 | SMD, -0.15 (-0.78, 0.48) | 39 | 18 | 21 | 0.65 | NA | NA | NA | No | No | 0.77 | ns |
| Amyloid Beta (CSF) Aß11–42 | 1 | SMD, 0.14 (-0.59, 0.88) | 31 | 11 | 20 | 0.71 | NA | NA | NA | No | No | 1.29 | ns |
| pGFAP-Positive Cells Pial surface Hippocampus | 1 | OR, 1.29 (0.14, 12.07) | 30 | 15 | 15 | 0.82 | NA | NA | NA | No | No | 1.29 | ns |
| Astrocytic cell area Hippocampus | 1 | SMD, -0.14 (-0.8, 0.53) | 35 | 19 | 16 | 0.69 | NA | NA | NA | No | No | 0.78 | ns |
| Astrocytosis in the Molecular Layer Dentate gyrus | 1 | SMD, -0.13 (-1.22, 0.96) | 13 | 6 | 7 | 0.82 | NA | NA | NA | No | No | 0.79 | ns |
| MDNT volume Mediodorsal thalamic nuclei | 1 | SMD, 0.13 (-0.6, 0.86) | 29 | 14 | 15 | 0.73 | NA | NA | NA | No | No | 1.26 | ns |
| GFAP 43 BA46 | 1 | SMD, 0.12 (-0.5, 0.74) | 40 | 20 | 20 | 0.7 | NA | NA | NA | No | No | 1.24 | ns |
| GFAP 41 BA10 | 1 | SMD, -0.12 (-0.74, 0.5) | 40 | 20 | 20 | 0.71 | NA | NA | NA | No | No | 0.81 | ns |
| Density of ependymal cells Subventricular zone | 1 | SMD, -0.12 (-0.83, 0.6) | 30 | 15 | 15 | 0.75 | NA | NA | NA | No | No | 0.81 | ns |
| Bcl-2 BA10 | 1 | SMD, 0.12 (-0.5, 0.74) | 40 | 20 | 20 | 0.71 | NA | NA | NA | No | No | 1.23 | ns |
| [3H]PK11195 binding (gliosis) Superior temporal cortex Temporal cortex | 1 | SMD, 0.11 (-0.79, 1.01) | 19 | 9 | 10 | 0.81 | NA | NA | NA | No | No | 1.22 | ns |
| Glial number Area sg24 Ventral PFC | 1 | SMD, 0.11 (-0.73, 0.95) | 22 | 11 | 11 | 0.8 | NA | NA | NA | No | No | 1.22 | ns |
| GFAP-positive astrocyte counts (per mm2) CA1 | 2 | SMD, -0.11 (-0.7, 0.49) | 45 | 21 | 24 | 0.72 | 0% | NA | NA | No | No | 0.82 | ns |
| THBS4 Anterior Cingulate gyrus White Matter | 1 | SMD, -0.11 (-0.74, 0.52) | 39 | 18 | 21 | 0.73 | NA | NA | NA | No | No | 0.82 | ns |
| GFAP-positive astrocyte counts (per mm2) Orbital frontal cortex | 2 | SMD, -0.1 (-1.28, 1.07) | 45 | 21 | 24 | 0.86 | 72% | NA | NA | No | No | 0.83 | ns |
| [3H]PK11195 binding (gliosis) Angular cortex Parietal cortex | 1 | SMD, -0.1 (-0.96, 0.76) | 21 | 11 | 10 | 0.82 | NA | NA | NA | No | No | 0.83 | ns |
| Amyloid Beta (post-mortem) midfrontal cortex - 2332 | 1 | SMD, -0.09 (-1.33, 1.15) | 10 | 5 | 5 | 0.88 | NA | NA | NA | No | No | 0.84 | ns |
| Brain weight SZ-male | 14 | SMD, -0.09 (-0.34, 0.15) | 603 | 257 | 346 | 0.46 | 47% | -0.82, 0.63 | No | No | Yes | 0.84 | ns |
| GFAP 41 BA9 | 1 | SMD, -0.1 (-0.72, 0.52) | 40 | 20 | 20 | 0.76 | NA | NA | NA | No | No | 0.84 | ns |
| Glia cell number Putamen left | 1 | SMD, -0.1 (-1.02, 0.83) | 18 | 9 | 9 | 0.84 | NA | NA | NA | No | No | 0.84 | ns |
| Glial density Layers V-VI dACC | 1 | SMD, 0.09 (-0.68, 0.87) | 26 | 14 | 12 | 0.81 | NA | NA | NA | No | No | 1.19 | ns |
| [3H]PK11195 binding (gliosis) Eye-movement area Prefrontal cortex | 1 | SMD, 0.09 (-0.78, 0.95) | 21 | 12 | 9 | 0.84 | NA | NA | NA | No | No | 1.17 | ns |
| pGFAP-Positive Cells Layer I interlaminar cells Prefrontal Cortex | 1 | OR, 1.16 (0.18, 7.27) | 30 | 15 | 15 | 0.88 | NA | NA | NA | No | No | 1.16 | ns |
| Microglial cell-density (cells/mm3) DLPC-L | 1 | SMD, 0.08 (-0.61, 0.78) | 32 | 16 | 16 | 0.82 | NA | NA | NA | No | No | 1.16 | ns |
| Neuronal density IV Dorsolateral prefrontal cortex (DLPFC) right (layer) | 1 | SMD, -0.08 (-0.96, 0.8) | 20 | 10 | 10 | 0.86 | NA | NA | NA | No | No | 0.87 | ns |
| HLA-B (smokers) Hippocampus | 1 | SMD, 0.07 (-0.48, 0.62) | 51 | 28 | 23 | 0.8 | NA | NA | NA | No | No | 1.14 | ns |
| Amyloid Beta (post-mortem) entorhinal cortex - Thioflavine S | 2 | SMD, 0.07 (-0.96, 1.09) | 20 | 15 | 5 | 0.9 | 0% | NA | NA | No | No | 1.13 | ns |
| Glutamine synthetase (GS) inmmunoreactivity A11/47 Prefrontal Cortex | 1 | SMD, 0.07 (-0.65, 0.78) | 30 | 15 | 15 | 0.85 | NA | NA | NA | No | No | 1.13 | ns |
| Capillary cross-sectional area (µm2) Prefrontal Cortex | 1 | SMD, -0.06 (-0.61, 0.48) | 52 | 26 | 26 | 0.82 | NA | NA | NA | No | No | 0.89 | ns |
| N-acetylaspartylglutamate (acetyl moiety, 2CH3, 2.04 ppm) Internal Capsule | 1 | SMD, -0.06 (-0.8, 0.69) | 28 | 15 | 13 | 0.88 | NA | NA | NA | No | No | 0.90 | ns |
| Phospate-activated glutaminase (GL) Anterior Cingulate gyrus deep layers (IV-VI) | 1 | SMD, -0.06 (-0.69, 0.57) | 39 | 18 | 21 | 0.86 | NA | NA | NA | No | No | 0.90 | ns |
| Oligodendrocyte density Substantia Nigra | 1 | SMD, -0.06 (-0.85, 0.72) | 25 | 12 | 13 | 0.88 | NA | NA | NA | No | No | 0.90 | ns |
| GFAP 41 BA46 | 1 | SMD, -0.05 (-0.67, 0.57) | 40 | 20 | 20 | 0.86 | NA | NA | NA | No | No | 0.91 | ns |
| Oligodendrocyte density Subgenual cingulate cortex White matter crown | 1 | SMD, -0.05 (-0.82, 0.71) | 29 | 10 | 19 | 0.89 | NA | NA | NA | No | No | 0.91 | ns |
| GFAP-positive astrocyte counts (per mm2) Visual cortex | 2 | SMD, -0.04 (-0.66, 0.57) | 45 | 21 | 24 | 0.89 | 5% | NA | NA | No | No | 0.92 | ns |
| Astrocytes total number (106) Basolateral nucleus of amygdala | 1 | SMD, -0.04 (-0.84, 0.76) | 24 | 12 | 12 | 0.92 | NA | NA | NA | No | No | 0.93 | ns |
| Microglial cell-density (cells/mm3) ACC-R | 3 | SMD, -0.04 (-0.61, 0.52) | 64 | 36 | 28 | 0.89 | 21% | -4.59, 4.51 | No | No | No | 0.93 | ns |
| VIM Anterior Cingulate gyrus superficial layers (I-III) | 1 | SMD, 0.04 (-0.59, 0.67) | 39 | 18 | 21 | 0.91 | NA | NA | NA | No | No | 1.07 | ns |
| Microglial cell-density (cells/mm3) MD-l | 1 | SMD, 0.04 (-0.65, 0.73) | 32 | 16 | 16 | 0.91 | NA | NA | NA | No | No | 1.07 | ns |
| Amyloid Beta (post-mortem) entorhinal cortex - 2332 | 3 | SMD, -0.04 (-0.97, 0.9) | 57 | 38 | 19 | 0.94 | 46% | -9.62, 9.55 | No | No | No | 0.94 | ns |
| Glial cells numbers CA2 Hippocampus | 1 | SMD, -0.03 (-1.23, 1.17) | 12 | 4 | 8 | 0.97 | NA | NA | NA | No | No | 0.95 | ns |
| 3H-SP binding sites density (fmol/mg tissue) Basal amygdala | 1 | SMD, -0.03 (-0.76, 0.7) | 29 | 14 | 15 | 0.93 | NA | NA | NA | No | No | 0.95 | ns |
| GFAP 43 BA40 | 1 | SMD, 0.03 (-0.59, 0.65) | 40 | 20 | 20 | 0.94 | NA | NA | NA | No | No | 1.05 | ns |
| Pyramidal Neuronal density II DLPFC left (layer) | 1 | SMD, 0.03 (-0.85, 0.9) | 20 | 10 | 10 | 0.95 | NA | NA | NA | No | No | 1.05 | ns |
| Glial density Layers II-III dACC | 1 | SMD, 0.03 (-0.67, 0.72) | 32 | 16 | 16 | 0.94 | NA | NA | NA | No | No | 1.05 | ns |
| GFAP protein expression Hippocampus | 1 | SMD, -0.02 (-0.58, 0.54) | 50 | 23 | 27 | 0.94 | NA | NA | NA | No | No | 0.96 | ns |
| HLA-A (no inflamation) Hippocampus | 1 | SMD, -0.02 (-0.5, 0.45) | 68 | 30 | 38 | 0.92 | NA | NA | NA | No | No | 0.96 | ns |
| Glia size Planum temporale | 1 | SMD, -0.02 (-0.74, 0.69) | 30 | 15 | 15 | 0.95 | NA | NA | NA | No | No | 0.96 | ns |
| Amyloid Beta (post-mortem) subiculum - 2332 | 1 | SMD, -0.02 (-0.68, 0.65) | 37 | 23 | 14 | 0.95 | NA | NA | NA | No | No | 0.97 | ns |
| GFAP-positive astrocyte counts (per mm2) Enthorrinal cortex | 2 | SMD, -0.02 (-0.77, 0.73) | 45 | 21 | 24 | 0.97 | 34% | NA | NA | No | No | 0.97 | ns |
| GFAP 41 BA40 | 1 | SMD, 0.01 (-0.61, 0.63) | 40 | 20 | 20 | 0.96 | NA | NA | NA | No | No | 1.03 | ns |
| Glutamine synthetase (GS) inmmunoreactivity A9 Prefrontal Cortex | 1 | SMD, -0.01 (-0.73, 0.7) | 30 | 15 | 15 | 0.98 | NA | NA | NA | No | No | 0.98 | ns |
| Microglial cell-density (cells/mm3) Dorsolateral prefrontal cortex (DLPFC)-R | 2 | SMD, -0.01 (-1.11, 1.08) | 32 | 20 | 12 | 0.99 | 55% | NA | NA | No | No | 0.98 | ns |
| Oligodendroglia total number (106) Ventral pallidum | 1 | SMD, 0.01 (-0.79, 0.81) | 24 | 12 | 12 | 0.98 | NA | NA | NA | No | No | 1.02 | ns |
| [3H]PK11195 binding (gliosis) Supramarginal cortex Parietal cortex | 1 | SMD, 0.01 (-0.89, 0.91) | 19 | 9 | 10 | 0.99 | NA | NA | NA | No | No | 1.01 | ns |
| Amyloid Beta (post-mortem) entorhinal cortex - PHF1 | 2 | SMD, 0 (-1.02, 1.02) | 20 | 15 | 5 | 1.0 | 0% | NA | NA | No | No | 1.00 | ns |
| Amyloid Beta (post-mortem) CA1 - PHF1 | 1 | SMD, 0 (-1.24, 1.24) | 10 | 5 | 5 | 1.0 | NA | NA | NA | No | No | 1.00 | ns |
| MOP ventral Internal Capsule | 1 | SMD, 0 (-0.8, 0.8) | 25 | 15 | 10 | 1.0 | NA | NA | NA | No | No | 1.00 | ns |
| Glycerophosphorylcholine (choline moiety, N(CH3)3, 3.21 ppm) Internal Capsule | 1 | SMD, 0 (-0.74, 0.74) | 28 | 15 | 13 | 1.0 | NA | NA | NA | No | No | 1.00 | ns |
| AQP4 Anterior Cingulate gyrus superficial layers (I-III) | 1 | SMD, 0 (-0.63, 0.63) | 39 | 18 | 21 | 1.0 | NA | NA | NA | No | No | 1.00 | ns |
| VIM Anterior Cingulate gyrus deep layers (IV-VI) | 1 | SMD, 0 (-0.63, 0.63) | 39 | 18 | 21 | 1.0 | NA | NA | NA | No | No | 1.00 | ns |
| GFAP ratio Motor cortex | 1 | SMD, 0 (-1.15, 1.15) | 12 | 5 | 7 | 1.0 | NA | NA | NA | No | No | 1.00 | ns |
| GFAP ratio Inf. temporal cortex | 1 | SMD, 0 (-1.15, 1.15) | 12 | 5 | 7 | 1.0 | NA | NA | NA | No | No | 1.00 | ns |
| GFAP ratio Parahippocampal cortex | 1 | SMD, 0 (-1.15, 1.15) | 12 | 5 | 7 | 1.0 | NA | NA | NA | No | No | 1.00 | ns |
| pGFAP-Positive Cells Layer II Prefrontal Cortex | 1 | OR, 1 (0.06, 17.62) | 30 | 15 | 15 | 1.0 | NA | NA | NA | No | No | 1.00 | ns |
| pGFAP-Positive Cells Hilus Hippocampus | 1 | OR, 1 (0.17, 5.98) | 30 | 15 | 15 | 1.0 | NA | NA | NA | No | No | 1.00 | ns |
| Hypocellular gap width (mm) Subventricular zone | 1 | SMD, 0 (-0.72, 0.72) | 30 | 15 | 15 | 1.0 | NA | NA | NA | No | No | 1.00 | ns |
| Microglial cell-density (cells/mm3) MD-R | 3 | SMD, 0 (-0.88, 0.87) | 64 | 36 | 28 | 1.0 | 60% | -9.6, 9.6 | No | No | No | 1.00 | ns |
| Neuronal density I Dorsolateral prefrontal cortex (DLPFC) right (layer) | 1 | SMD, 0 (-0.88, 0.88) | 20 | 10 | 10 | 1.0 | NA | NA | NA | No | No | 1.00 | ns |
| Glial density (males) III Fusiform cortex right (layer) | 1 | SMD, 0 (-1.09, 1.09) | 13 | 6 | 7 | 1.0 | NA | NA | NA | No | No | 1.00 | ns |
| Glial density (males) V Fusiform cortex right (layer) | 1 | SMD, 0 (-1.09, 1.09) | 13 | 6 | 7 | 1.0 | NA | NA | NA | No | No | 1.00 | ns |
| Glial density (males) III Fusiform cortex left (layer) | 1 | SMD, 0 (-1.09, 1.09) | 13 | 6 | 7 | 1.0 | NA | NA | NA | No | No | 1.00 | ns |
| Glial density (males) V Fusiform cortex left (layer) | 1 | SMD, 0 (-1.09, 1.09) | 13 | 6 | 7 | 1.0 | NA | NA | NA | No | No | 1.00 | ns |
| Glial density (females) III Fusiform cortex right (layer) | 1 | SMD, 0 (-1.19, 1.18) | 11 | 5 | 6 | 1.0 | NA | NA | NA | No | No | 1.00 | ns |
| Glial density (females) V Fusiform cortex right (layer) | 1 | SMD, 0 (-1.19, 1.19) | 11 | 5 | 6 | 1.0 | NA | NA | NA | No | No | 1.00 | ns |
| Glial density (females) III Fusiform cortex left (layer) | 1 | SMD, 0 (-1.19, 1.19) | 11 | 5 | 6 | 1.0 | NA | NA | NA | No | No | 1.00 | ns |
| Glial density (females) V Fusiform cortex left (layer) | 1 | SMD, 0 (-1.19, 1.18) | 11 | 5 | 6 | 1.0 | NA | NA | NA | No | No | 1.00 | ns |
| Glia number Parvocellular Lateral geniculate nucleus (thalamus) | 1 | SMD, 0 (-0.72, 0.72) | 30 | 15 | 15 | 1.0 | NA | NA | NA | No | No | 1.00 | ns |
| 3H-SP binding sites density (fmol/mg tissue) Lateral amygdala | 1 | SMD, 0 (-0.73, 0.73) | 29 | 14 | 15 | 1.0 | NA | NA | NA | No | No | 1.00 | ns |

ALDH1L1 - Aldehyde dehydrogenase 1 family, member L1, CI – confidence interval, CE – class of evidence, CSF – cerebrospinal fluid, eOR – equivalent odds ratio, Egger – significant Egger test, ES – effect size, ESB – excess significance bias, GFAP - glial fibrillary acidic protein, k – number of studies for each factor, LS - largest study with significant effect, N – total number of participants, NA – not assessable, ns – not significant, OR – odds ratio, pGFAP – plasma glial fibrillary acidic protein, PI – prediction interval, SMD – standardized mean difference.

**sTable 6.** Level of evidence for the association of other biomarkers and psychotic disorders^102–110^

| **Factor** | **k** | **ES (95% CI)** | **Features used for classification of level of evidence** | | | | | | | | | **eOR** | **Class** |
| --- | --- | --- | --- | --- | --- | --- | --- | --- | --- | --- | --- | --- | --- |
|  |  |  | **N** | **Cases** | **Controls** | **p** | **I^2^** | **PI 95% CI** | **Egger** | **ESB** | **LS** |  |  |
| Minor physical anomalies | 14 | SMD, 0.99 (0.64, 1.34) | 2160 | 1153 | 1007 | <0.000001 | 93% | -0.45, 2.42 | No | Yes | Yes | 5.99 | II |
| High frequency heart rate variability | 28 | SMD, -0.99 (-1.41, -0.58) | 3008 | 1330 | 1678 | 0.000002 | 98% | -3.24, 1.25 | Yes | Yes | Yes | 0.16 | III |
| Relative abundance of Neisseria_subflava in oropharynx | 1 | SMD, 13.26 (9.73, 16.78) | 32 | 16 | 16 | <0.000001 | NA | NA | NA | No | Yes | >100 | IV |
| Blood anti-Saccharomyces cerevisiae Immunoglobulin G (IgG) level  Saccharomyces cerevisiae | 2 | SMD, 4.05 (3.24, 4.87) | 467 | 260 | 207 | <0.000001 | 82% | NA | NA | No | Yes | >100 | IV |
| Sleep efficiency % averaged night 2+3 | 1 | SMD, 3.67 (2.13, 5.21) | 20 | 10 | 10 | 0.000003 | NA | NA | NA | No | Yes | >100 | IV |
| Neurological Examination Scale | 1 | SMD, 3.28 (2.59, 3.98) | 79 | 29 | 50 | <0.000001 | NA | NA | NA | No | Yes | >100 | IV |
| Relative abundance of Neisseria_flavescens in oropharynx | 1 | SMD, 2.83 (1.82, 3.84) | 32 | 16 | 16 | <0.000001 | NA | NA | NA | No | Yes | >100 | IV |
| Relative abundance of Fusobacterium_periodonticum in oropharynx | 1 | SMD, 2.73 (1.74, 3.73) | 32 | 16 | 16 | <0.000001 | NA | NA | NA | No | Yes | >100 | IV |
| WCST Achieved categories | 1 | SMD, -2.35 (-3.1, -1.6) | 48 | 26 | 22 | <0.000001 | NA | NA | NA | No | Yes | 0.01 | IV |
| Total time in bed | 3 | SMD, 2.39 (0.02, 4.76) | 97 | 50 | 47 | 0.048 | 91% | -27.53, 32.31 | No | No | Yes | 76.34 | IV |
| NES Total score | 1 | SMD, 2.33 (1.58, 3.07) | 48 | 26 | 22 | <0.000001 | NA | NA | NA | No | Yes | 68.21 | IV |
| WCST Correct responses | 1 | SMD, -2.22 (-2.96, -1.49) | 48 | 26 | 22 | <0.000001 | NA | NA | NA | No | Yes | 0.02 | IV |
| Executive function: verbal memory-delayed recall (LDR) | 1 | SMD, -2.13 (-2.72, -1.55) | 73 | 43 | 30 | <0.000001 | NA | NA | NA | No | Yes | 0.02 | IV |
| Delta (0.5-2.0) | 2 | SMD, -2.28 (-3.4, -1.17) | 24 | 12 | 12 | 0.00006 | 0% | NA | NA | No | Yes | 0.02 | IV |
| Nonlinearity scores of EEG-C4 Awake | 1 | SMD, -2.08 (-3.28, -0.88) | 18 | 9 | 9 | 0.00067 | NA | NA | NA | No | Yes | 0.02 | IV |
| NSS scale total score | 2 | SMD, 2.04 (1.01, 3.06) | 84 | 50 | 34 | 0.0001 | 72% | NA | NA | No | Yes | 40.22 | IV |
| Amplitude (µV) delta half-wave count per hour 45-55 | 2 | SMD, -1.85 (-2.87, -0.83) | 24 | 12 | 12 | 0.00037 | 0% | NA | NA | No | Yes | 0.03 | IV |
| Amplitude (µV) delta half-wave count per hour 55< | 2 | SMD, -2.02 (-3.07, -0.96) | 24 | 12 | 12 | 0.00018 | 0% | NA | NA | No | Yes | 0.03 | IV |
| Delta half-wave count per hour in first sleep cycle | 2 | SMD, -1.99 (-3.04, -0.94) | 24 | 12 | 12 | 0.00021 | 0% | NA | NA | No | Yes | 0.03 | IV |
| Relative abundance of Streptococcus_sp._oral_taxon_071 in oropharynx | 1 | SMD, -1.98 (-2.84, -1.11) | 32 | 16 | 16 | 0.000007 | NA | NA | NA | No | Yes | 0.03 | IV |
| NES Sensorial integration | 1 | SMD, 1.78 (1.1, 2.46) | 48 | 26 | 22 | <0.000001 | NA | NA | NA | No | Yes | 25.39 | IV |
| Executive function: verbal memory-immediare recall (LIR) | 1 | SMD, -1.81 (-2.36, -1.25) | 73 | 43 | 30 | <0.000001 | NA | NA | NA | No | Yes | 0.04 | IV |
| Amplitude (µV) delta half-wave count per hour 35-45 | 2 | SMD, -1.73 (-2.72, -0.73) | 24 | 12 | 12 | 0.00065 | 0% | NA | NA | No | Yes | 0.04 | IV |
| Largest Lyapunov exponent of EEG-F4 REM | 1 | SMD, -1.72 (-2.84, -0.6) | 18 | 9 | 9 | 0.0025 | NA | NA | NA | No | Yes | 0.04 | IV |
| Early morning awake time (age adjusted) | 1 | SMD, 1.7 (0.7, 2.7) | 24 | 8 | 16 | 0.00082 | NA | NA | NA | No | Yes | 21.91 | IV |
| Intelligence quotient (IQ) | 1 | SMD, -1.71 (-2.25, -1.16) | 73 | 43 | 30 | <0.000001 | NA | NA | NA | No | Yes | 0.05 | IV |
| Stage 1 Amplitude | 1 | SMD, -1.69 (-2.61, -0.78) | 26 | 13 | 13 | 0.00029 | NA | NA | NA | No | Yes | 0.05 | IV |
| Sleep efficiency % | 1 | SMD, -1.6 (-2.34, -0.86) | 40 | 15 | 25 | 0.000021 | NA | NA | NA | No | Yes | 0.05 | IV |
| NSS total score (Heidelberg scale) | 3 | SMD, 1.63 (1.09, 2.17) | 100 | 78 | 22 | <0.000001 | 0% | -1.86, 5.12 | No | No | Yes | 19.32 | IV |
| WCST Perseverative errors | 1 | SMD, 1.61 (0.95, 2.27) | 48 | 26 | 22 | 0.000002 | NA | NA | NA | No | Yes | 18.61 | IV |
| Awakenings number (>60s) | 1 | SMD, 1.59 (0.75, 2.42) | 30 | 15 | 15 | 0.0002 | NA | NA | NA | No | Yes | 17.74 | IV |
| NSS total score (Neurological Evaluation Scale) | 2 | SMD, 1.58 (1.26, 1.9) | 200 | 98 | 102 | <0.000001 | 0% | NA | NA | No | Yes | 17.50 | IV |
| Delta half-wave count per hour in second sleep cycle | 2 | SMD, -1.54 (-2.5, -0.58) | 24 | 12 | 12 | 0.0016 | 0% | NA | NA | No | Yes | 0.06 | IV |
| Relative abundance of Streptococcus_gordonii in oropharynx | 1 | SMD, -1.56 (-2.37, -0.76) | 32 | 16 | 16 | 0.00014 | NA | NA | NA | No | Yes | 0.06 | IV |
| Postural tremor, hands | 1 | OR, 14.9 (1.78, 125.01) | 92 | 37 | 55 | 0.013 | NA | NA | NA | No | Yes | 14.90 | IV |
| Purdue pegboard test | 1 | SMD, -1.45 (-1.92, -0.99) | 92 | 37 | 55 | <0.000001 | NA | NA | NA | No | Yes | 0.07 | IV |
| Relative alpha spectral amplitude uV in central region | 1 | SMD, -1.46 (-2.26, -0.67) | 40 | 10 | 30 | 0.00029 | NA | NA | NA | No | Yes | 0.07 | IV |
| Spindle density/minute | 1 | SMD, -1.43 (-2.16, -0.71) | 38 | 21 | 17 | 0.0001 | NA | NA | NA | No | Yes | 0.07 | IV |
| Initial Extrapyramidal | 1 | SMD, 1.46 (0.98, 1.95) | 83 | 43 | 40 | <0.000001 | NA | NA | NA | No | Yes | 14.25 | IV |
| Awakenings number (<60s) | 1 | SMD, 1.45 (0.63, 2.26) | 30 | 15 | 15 | 0.00051 | NA | NA | NA | No | Yes | 13.81 | IV |
| REM periods number | 1 | SMD, 1.44 (0.43, 2.44) | 20 | 10 | 10 | 0.0051 | NA | NA | NA | No | Yes | 13.54 | IV |
| Psychomotor functioning: Digit Symbol (DS) | 1 | SMD, -1.38 (-1.9, -0.86) | 73 | 43 | 30 | <0.000001 | NA | NA | NA | No | Yes | 0.08 | IV |
| NES Complex motor acts | 1 | SMD, 1.34 (0.71, 1.98) | 48 | 26 | 22 | 0.000032 | NA | NA | NA | No | Yes | 11.42 | IV |
| Total sleep time (prestudy actigraphy) | 1 | SMD, 1.33 (0.62, 2.04) | 38 | 21 | 17 | 0.00025 | NA | NA | NA | No | Yes | 11.18 | IV |
| Amplitude (µV) delta half-wave count per hour 25-35 | 2 | SMD, -1.35 (-2.27, -0.43) | 24 | 12 | 12 | 0.0042 | 0% | NA | NA | No | Yes | 0.09 | IV |
| Delta half-wave count per hour total | 2 | SMD, -1.35 (-2.27, -0.43) | 24 | 12 | 12 | 0.0042 | 0% | NA | NA | No | Yes | 0.09 | IV |
| Relative abundance of Streptococcus_thermophilus in oropharynx | 1 | SMD, -1.32 (-2.09, -0.54) | 32 | 16 | 16 | 0.00085 | NA | NA | NA | No | Yes | 0.09 | IV |
| Fist–ring R | 2 | OR, 10.95 (1.36, 88.37) | 107 | 60 | 47 | 0.025 | 0% | NA | NA | No | No | 10.95 | IV |
| Fist–ring L | 2 | OR, 10.95 (1.36, 88.37) | 107 | 60 | 47 | 0.025 | 0% | NA | NA | No | No | 10.95 | IV |
| NES total | 2 | SMD, 1.32 (0.34, 2.3) | 66 | 44 | 22 | 0.0085 | 66% | NA | NA | No | Yes | 10.94 | IV |
| AIMS extremity movements | 1 | SMD, 1.32 (0.66, 1.97) | 44 | 22 | 22 | 0.000086 | NA | NA | NA | No | Yes | 10.90 | IV |
| 3-years Follow-up Disinhibition | 1 | SMD, 1.29 (0.81, 1.76) | 83 | 43 | 40 | <0.000001 | NA | NA | NA | No | Yes | 10.30 | IV |
| Oszeretski | 2 | OR, 10.25 (1.25, 83.95) | 107 | 60 | 47 | 0.03 | 0% | NA | NA | No | No | 10.25 | IV |
| Gait-test walking time | 1 | SMD, 1.27 (0.81, 1.72) | 92 | 37 | 55 | <0.000001 | NA | NA | NA | No | Yes | 9.95 | IV |
| Relative beta2 spectral amplitude uV in occipital region | 1 | SMD, 1.26 (0.48, 2.03) | 40 | 10 | 30 | 0.0014 | NA | NA | NA | No | Yes | 9.76 | IV |
| Tendon reflexes, achilles | 1 | OR, 9.47 (3.08, 29.12) | 92 | 37 | 55 | 0.000087 | NA | NA | NA | No | Yes | 9.47 | IV |
| Sleep maintenance % (age adjusted) | 1 | SMD, -1.23 (-2.16, -0.3) | 24 | 8 | 16 | 0.0095 | NA | NA | NA | No | Yes | 0.11 | IV |
| Symbolic dynamic measures of EEG-C4 Awake (word count) | 1 | SMD, -1.22 (-2.25, -0.19) | 18 | 9 | 9 | 0.02 | NA | NA | NA | No | Yes | 0.11 | IV |
| Largest Lyapunov exponent of EEG-C4 REM | 1 | SMD, -1.21 (-2.24, -0.19) | 18 | 9 | 9 | 0.021 | NA | NA | NA | No | Yes | 0.11 | IV |
| Change of center point of force velocity from eyes open to eyes closed condition % | 1 | SMD, -1.23 (-1.96, -0.49) | 36 | 22 | 14 | 0.001 | NA | NA | NA | No | Yes | 0.11 | IV |
| Wake after sleep onset min | 1 | SMD, 1.22 (0.52, 1.91) | 40 | 15 | 25 | 0.00065 | NA | NA | NA | No | Yes | 9.07 | IV |
| Tonus, neck | 1 | OR, 8.52 (1.72, 42.15) | 92 | 37 | 55 | 0.0087 | NA | NA | NA | No | Yes | 8.52 | IV |
| Executive function: Hopkins—immediate recall (HT) | 1 | SMD, -1.15 (-1.65, -0.64) | 73 | 43 | 30 | 0.000008 | NA | NA | NA | No | Yes | 0.12 | IV |
| Memory: Hopkins-delayed recall (HD) | 1 | SMD, -1.19 (-1.7, -0.68) | 73 | 43 | 30 | 0.000004 | NA | NA | NA | No | Yes | 0.12 | IV |
| Total recording period | 1 | SMD, -1.11 (-1.99, -0.22) | 27 | 8 | 19 | 0.014 | NA | NA | NA | No | Yes | 0.13 | IV |
| Symbolic dynamic measures of EEG-C4 REM (word count) | 1 | SMD, -1.14 (-2.15, -0.12) | 18 | 9 | 9 | 0.028 | NA | NA | NA | No | Yes | 0.13 | IV |
| Awake time (age adjusted) | 1 | SMD, 1.12 (0.2, 2.04) | 24 | 8 | 16 | 0.016 | NA | NA | NA | No | Yes | 7.64 | IV |
| Motor coordination (NES) | 2 | SMD, 1.1 (0.55, 1.65) | 66 | 44 | 22 | 0.000086 | 0% | NA | NA | No | Yes | 7.40 | IV |
| Others (NES) | 2 | SMD, 1.1 (0.47, 1.73) | 66 | 44 | 22 | 0.00063 | 23% | NA | NA | No | Yes | 7.37 | IV |
| Gait-test walking steps | 1 | SMD, 1.1 (0.65, 1.55) | 92 | 37 | 55 | 0.000001 | NA | NA | NA | No | Yes | 7.37 | IV |
| Stage 2: 4th Quarter (S2q4) duration | 1 | SMD, 1.09 (0.3, 1.87) | 29 | 14 | 15 | 0.0068 | NA | NA | NA | No | Yes | 7.19 | IV |
| Amplitude (µV) delta half-wave count per hour 15-25 | 2 | SMD, -1.09 (-1.97, -0.21) | 24 | 12 | 12 | 0.015 | 0% | NA | NA | No | No | 0.14 | IV |
| Relative alpha spectral amplitude uV in temporal region | 1 | SMD, -1.08 (-1.84, -0.33) | 40 | 10 | 30 | 0.0051 | NA | NA | NA | No | Yes | 0.14 | IV |
| The slow component CoM cm, eyes open on yielding surface, antero-posterior | 1 | SMD, 1.07 (0.26, 1.88) | 29 | 11 | 18 | 0.0093 | NA | NA | NA | No | Yes | 6.97 | IV |
| Tendon reflexes, biceps | 1 | OR, 6.91 (2.04, 23.42) | 92 | 37 | 55 | 0.0019 | NA | NA | NA | No | Yes | 6.91 | IV |
| Sleep latency | 20 | SMD, 1.06 (0.62, 1.49) | 570 | 282 | 288 | 0.000002 | 82% | -0.83, 2.95 | Yes | Yes | Yes | 6.82 | IV |
| 3-years Follow-up Sensory integration | 1 | SMD, 1.06 (0.6, 1.52) | 83 | 43 | 40 | 0.000007 | NA | NA | NA | No | Yes | 6.80 | IV |
| 6-minute walking distance | 2 | SMD, -1.06 (-1.42, -0.69) | 180 | 140 | 40 | <0.000001 | 0% | NA | NA | No | Yes | 0.15 | IV |
| Memory: Hopkins-recognition (HREC) | 1 | SMD, -1.04 (-1.54, -0.54) | 73 | 43 | 30 | 0.000041 | NA | NA | NA | No | Yes | 0.15 | IV |
| Memory: nonverbal memory-immediate visual reproduction (VRI) | 1 | SMD, -1.04 (-1.54, -0.54) | 73 | 43 | 30 | 0.000042 | NA | NA | NA | No | Yes | 0.15 | IV |
| Spectral Power during Stage 2 sleep µV2/Hz - Theta (4-8Hz) | 1 | SMD, -1.04 (-1.72, -0.35) | 40 | 15 | 25 | 0.0029 | NA | NA | NA | No | Yes | 0.15 | IV |
| Spindle density/min | 1 | SMD, -1.06 (-1.75, -0.38) | 40 | 15 | 25 | 0.0023 | NA | NA | NA | No | Yes | 0.15 | IV |
| Symbolic dynamic measures of EEG-F4 REM (SD of the word sequence) | 1 | SMD, -1.03 (-2.03, -0.03) | 18 | 9 | 9 | 0.043 | NA | NA | NA | No | Yes | 0.15 | IV |
| 3-years Follow-up Motor coordination | 1 | SMD, 1.04 (0.58, 1.5) | 83 | 43 | 40 | 0.00001 | NA | NA | NA | No | Yes | 6.56 | IV |
| NES Motor coordination | 1 | SMD, 1.01 (0.41, 1.62) | 48 | 26 | 22 | 0.0011 | NA | NA | NA | No | Yes | 6.25 | IV |
| Attention: Verbal Fluency total (VFT) | 1 | SMD, -1 (-1.5, -0.51) | 73 | 43 | 30 | 0.000073 | NA | NA | NA | No | Yes | 0.16 | IV |
| REM sleep latency min | 1 | SMD, -1 (-1.94, -0.06) | 20 | 10 | 10 | 0.037 | NA | NA | NA | No | Yes | 0.16 | IV |
| Symbolic dynamic measures of EEG-C4 REM (SD of the word sequence) | 1 | SMD, -1.03 (-2.03, -0.03) | 18 | 9 | 9 | 0.044 | NA | NA | NA | No | Yes | 0.16 | IV |
| Memory: nonverbal memory-delayed visual reproduction (VRD) | 1 | SMD, -0.97 (-1.46, -0.48) | 73 | 43 | 30 | 0.00012 | NA | NA | NA | No | Yes | 0.17 | IV |
| Amplitude (µV) delta half-wave count per hour 5-15 | 2 | SMD, 0.97 (0.1, 1.83) | 24 | 12 | 12 | 0.029 | 0% | NA | NA | No | No | 5.77 | IV |
| Sleep stage shifts number | 1 | SMD, 0.96 (0.02, 1.89) | 20 | 10 | 10 | 0.045 | NA | NA | NA | No | Yes | 5.68 | IV |
| Root mean square of successive R–R interval differences (RMSSD) | 22 | SMD, -0.95 (-1.38, -0.52) | 2359 | 951 | 1408 | 0.000017 | 89% | -3.05, 1.16 | No | No | Yes | 0.18 | IV |
| Frequency (Hz) delta half-wave count per hour 2.6 -3.0 | 2 | SMD, -0.93 (-1.79, -0.07) | 24 | 12 | 12 | 0.034 | 0% | NA | NA | No | No | 0.19 | IV |
| Relative alpha spectral amplitude uV in occipital region | 1 | SMD, -0.92 (-1.67, -0.17) | 40 | 10 | 30 | 0.016 | NA | NA | NA | No | Yes | 0.19 | IV |
| Spectral Power during Stage 2 sleep µV2/Hz - Sigma (12-15Hz) | 1 | SMD, -0.92 (-1.59, -0.24) | 40 | 15 | 25 | 0.0077 | NA | NA | NA | No | Yes | 0.19 | IV |
| Spindle number | 2 | SMD, -0.9 (-1.63, -0.17) | 78 | 36 | 42 | 0.016 | 57% | NA | NA | No | No | 0.20 | IV |
| NREM latency | 1 | SMD, 0.89 (0.12, 1.65) | 29 | 14 | 15 | 0.024 | NA | NA | NA | No | Yes | 4.99 | IV |
| Fist edge–palm R | 2 | OR, 4.94 (1.55, 15.78) | 107 | 60 | 47 | 0.0071 | 0% | NA | NA | No | Yes | 4.94 | IV |
| Stage 3 latency | 1 | SMD, 0.86 (0.21, 1.51) | 40 | 20 | 20 | 0.0093 | NA | NA | NA | No | Yes | 4.79 | IV |
| Mean sway in the medial-lateral direction cm | 1 | SMD, 0.85 (0.37, 1.34) | 72 | 36 | 36 | 0.00054 | NA | NA | NA | No | Yes | 4.70 | IV |
| Mean sway in the anterior-posterior direction cm | 1 | SMD, 0.85 (0.37, 1.34) | 72 | 36 | 36 | 0.00054 | NA | NA | NA | No | Yes | 4.70 | IV |
| Mean sway cm | 1 | SMD, 0.85 (0.37, 1.34) | 72 | 36 | 36 | 0.00054 | NA | NA | NA | No | Yes | 4.70 | IV |
| AIMS global judgment | 1 | SMD, 0.84 (0.22, 1.46) | 44 | 22 | 22 | 0.008 | NA | NA | NA | No | Yes | 4.56 | IV |
| Individual Splindle Sigma coherence (0-1) | 1 | SMD, -0.83 (-1.5, -0.16) | 38 | 21 | 17 | 0.015 | NA | NA | NA | No | Yes | 0.22 | IV |
| REM latency minus awake | 1 | SMD, -0.82 (-1.52, -0.12) | 35 | 20 | 15 | 0.021 | NA | NA | NA | No | Yes | 0.23 | IV |
| Relative alpha spectral amplitude uV in frontal region | 1 | SMD, -0.8 (-1.54, -0.06) | 40 | 10 | 30 | 0.034 | NA | NA | NA | No | Yes | 0.24 | IV |
| Sleep efficiency | 18 | SMD, -0.8 (-1.18, -0.41) | 482 | 235 | 247 | 0.000055 | 72% | -2.35, 0.76 | No | Yes | Yes | 0.24 | IV |
| Position 8 mean sway cm | 1 | SMD, 0.78 (0.3, 1.26) | 72 | 36 | 36 | 0.0014 | NA | NA | NA | No | Yes | 4.13 | IV |
| Pronation-supination test | 1 | SMD, 0.78 (0.35, 1.21) | 92 | 37 | 55 | 0.00042 | NA | NA | NA | No | Yes | 4.10 | IV |
| Relative spectral amplitude uV, beta2 | 1 | SMD, 0.76 (0.03, 1.5) | 40 | 10 | 30 | 0.042 | NA | NA | NA | No | Yes | 4.00 | IV |
| Total sleep period | 3 | SMD, -0.77 (-1.16, -0.37) | 108 | 55 | 53 | 0.00013 | 0% | -3.32, 1.78 | No | No | Yes | 0.25 | IV |
| Spectral Power during Stage 2 sleep µV2/Hz - Delta (1-4Hz) | 1 | SMD, -0.77 (-1.43, -0.1) | 40 | 15 | 25 | 0.023 | NA | NA | NA | No | Yes | 0.25 | IV |
| Position 8 sway in the medial-lateral direction cm | 1 | SMD, 0.75 (0.27, 1.23) | 72 | 36 | 36 | 0.0021 | NA | NA | NA | No | Yes | 3.91 | IV |
| Position 5 sway in the anterior-posterior direction cm | 1 | SMD, 0.73 (0.26, 1.21) | 72 | 36 | 36 | 0.0026 | NA | NA | NA | No | Yes | 3.78 | IV |
| Sway area mm2 | 1 | SMD, 0.73 (0.21, 1.24) | 64 | 27 | 37 | 0.0055 | NA | NA | NA | No | Yes | 3.73 | IV |
| Stage 4 duration | 4 | SMD, -0.71 (-1.38, -0.04) | 129 | 63 | 66 | 0.038 | 68% | -3.57, 2.15 | No | No | Yes | 0.27 | IV |
| Tendon reflexes, patella | 1 | OR, 3.7 (1.15, 11.95) | 92 | 37 | 55 | 0.028 | NA | NA | NA | No | Yes | 3.70 | IV |
| Fist edge–palm L | 2 | OR, 3.59 (1.22, 10.57) | 107 | 60 | 47 | 0.02 | 0% | NA | NA | No | No | 3.59 | IV |
| Low sigma power µV2/Hz | 1 | SMD, -0.71 (-1.37, -0.05) | 38 | 21 | 17 | 0.035 | NA | NA | NA | No | Yes | 0.28 | IV |
| Spindles amplitude µV | 1 | SMD, -0.69 (-1.35, -0.03) | 40 | 15 | 25 | 0.04 | NA | NA | NA | No | Yes | 0.28 | IV |
| WCST Failure to maintain set | 1 | SMD, 0.7 (0.11, 1.28) | 48 | 26 | 22 | 0.019 | NA | NA | NA | No | Yes | 3.55 | IV |
| NSS total score | 1 | SMD, 0.7 (0.28, 1.12) | 96 | 58 | 38 | 0.0012 | NA | NA | NA | No | Yes | 3.54 | IV |
| Blood anti-Saccharomyces cerevisiae Immunoglobulin G (IgG) level (seropositivity)  Saccharomyces cerevisiae | 1 | OR, 3.52 (1.48, 8.39) | 245 | 38 | 207 | 0.0045 | NA | NA | NA | No | Yes | 3.52 | IV |
| Sway area eyes open mm2 | 1 | SMD, 0.68 (0.17, 1.19) | 64 | 27 | 37 | 0.0087 | NA | NA | NA | No | Yes | 3.45 | IV |
| Initial Motor coordination | 1 | SMD, 0.67 (0.22, 1.11) | 83 | 43 | 40 | 0.0032 | NA | NA | NA | No | Yes | 3.34 | IV |
| Sway area eyes closed mm2 | 1 | SMD, 0.66 (0.15, 1.17) | 64 | 27 | 37 | 0.011 | NA | NA | NA | No | Yes | 3.34 | IV |
| No of finger taps per 10 s, preferred hand, occasion 1 | 1 | SMD, -0.65 (-1.08, -0.22) | 92 | 37 | 55 | 0.0028 | NA | NA | NA | No | Yes | 0.31 | IV |
| Position 5 mean sway cm | 1 | SMD, 0.64 (0.17, 1.12) | 72 | 36 | 36 | 0.0081 | NA | NA | NA | No | Yes | 3.20 | IV |
| WCST Non-perseverative errors | 1 | SMD, 0.64 (0.06, 1.22) | 48 | 26 | 22 | 0.031 | NA | NA | NA | No | Yes | 3.19 | IV |
| Soluble cluster of differentiation 14 (CD14) | 1 | OR, 3.09 (1.23, 7.8) | 219 | 141 | 78 | 0.017 | NA | NA | NA | No | Yes | 3.09 | IV |
| Initial Disinhibition | 1 | SMD, 0.6 (0.16, 1.04) | 83 | 43 | 40 | 0.0078 | NA | NA | NA | No | Yes | 2.96 | IV |
| Position 8 sway in the anterior-posterior direction cm | 1 | SMD, 0.58 (0.11, 1.06) | 72 | 36 | 36 | 0.016 | NA | NA | NA | No | Yes | 2.88 | IV |
| Position 3 mean sway cm | 1 | SMD, 0.58 (0.11, 1.06) | 72 | 36 | 36 | 0.016 | NA | NA | NA | No | Yes | 2.88 | IV |
| 3-years Follow-up Catatonia | 1 | SMD, 0.58 (0.14, 1.02) | 83 | 43 | 40 | 0.0099 | NA | NA | NA | No | Yes | 2.86 | IV |
| Initial Sensory integration | 1 | SMD, 0.55 (0.11, 0.99) | 83 | 43 | 40 | 0.013 | NA | NA | NA | No | Yes | 2.73 | IV |
| Initial Catatonia | 1 | SMD, 0.55 (0.11, 0.99) | 83 | 43 | 40 | 0.014 | NA | NA | NA | No | Yes | 2.72 | IV |
| Attention: Continuous Performance Test—attentiveness (CPTA) | 1 | SMD, -0.55 (-1.02, -0.07) | 73 | 43 | 30 | 0.024 | NA | NA | NA | No | Yes | 0.37 | IV |
| Stage 2 duration | 8 | SMD, -0.56 (-1.01, -0.1) | 190 | 97 | 93 | 0.016 | 53% | -1.81, 0.7 | No | No | Yes | 0.37 | IV |
| Position 9 sway in the anterior-posterior direction cm | 1 | SMD, 0.55 (0.07, 1.02) | 72 | 36 | 36 | 0.023 | NA | NA | NA | No | Yes | 2.69 | IV |
| Stage 3 duration | 5 | SMD, -0.54 (-0.87, -0.21) | 155 | 76 | 79 | 0.0015 | 4% | -1.11, 0.03 | No | No | Yes | 0.38 | IV |
| Position 6 sway in the medial-lateral direction cm | 1 | SMD, 0.53 (0.06, 1) | 72 | 36 | 36 | 0.028 | NA | NA | NA | No | Yes | 2.61 | IV |
| Position 6 mean sway cm | 1 | SMD, 0.53 (0.06, 1) | 72 | 36 | 36 | 0.028 | NA | NA | NA | No | Yes | 2.61 | IV |
| Awake time | 10 | SMD, 0.52 (0, 1.04) | 241 | 120 | 121 | 0.048 | 68% | -1.17, 2.21 | No | Yes | No | 2.57 | IV |
| Premorbid intelligence quotient (IQ) | 1 | SMD, -0.5 (-0.98, -0.03) | 73 | 43 | 30 | 0.038 | NA | NA | NA | No | Yes | 0.40 | IV |
| Position 10 sway in the medial-lateral direction cm | 1 | SMD, 0.49 (0.02, 0.96) | 72 | 36 | 36 | 0.04 | NA | NA | NA | No | Yes | 2.44 | IV |
| Position 10 mean sway cm | 1 | SMD, 0.48 (0.01, 0.95) | 72 | 36 | 36 | 0.045 | NA | NA | NA | No | Yes | 2.39 | IV |
| Total sleep time | 22 | SMD, -0.48 (-0.78, -0.19) | 749 | 371 | 378 | 0.0014 | 69% | -1.71, 0.74 | No | Yes | No | 0.42 | IV |
| Attention: Trail Making (TM) | 1 | SMD, 0.48 (0, 0.95) | 73 | 43 | 30 | 0.048 | NA | NA | NA | No | Yes | 2.38 | IV |
| Position 9 mean sway cm | 1 | SMD, 0.47 (0.01, 0.94) | 72 | 36 | 36 | 0.048 | NA | NA | NA | No | Yes | 2.36 | IV |
| 3-years Follow-up Pyramidal | 1 | SMD, 0.44 (0, 0.87) | 83 | 43 | 40 | 0.049 | NA | NA | NA | No | Yes | 2.21 | IV |
| Stage 1 duration | 8 | SMD, 0.42 (0.06, 0.77) | 190 | 97 | 93 | 0.023 | 21% | -0.39, 1.22 | No | No | No | 2.12 | IV |
| Stage 4 % | 9 | SMD, -0.39 (-0.72, -0.07) | 398 | 198 | 200 | 0.016 | 51% | -1.3, 0.51 | No | No | No | 0.49 | IV |
| Finger-thumb tapping right | 1 | SMD, 0.39 (0.08, 0.71) | 161 | 68 | 93 | 0.015 | NA | NA | NA | No | Yes | 2.03 | IV |
| Ozeretsky test | 1 | SMD, 0.38 (0.07, 0.7) | 161 | 68 | 93 | 0.017 | NA | NA | NA | No | Yes | 2.01 | IV |
| Fist-edge palm test right | 1 | SMD, 0.38 (0.07, 0.7) | 161 | 68 | 93 | 0.017 | NA | NA | NA | No | Yes | 2.00 | IV |
| REM time | 9 | SMD, -0.37 (-0.63, -0.1) | 230 | 114 | 116 | 0.0066 | 0% | -0.69, -0.05 | No | No | No | 0.51 | IV |
| Integrative abnormalities | 1 | SMD, 0.37 (0.11, 0.63) | 239 | 93 | 146 | 0.0058 | NA | NA | NA | No | Yes | 1.95 | IV |
| Total neurological abnormalities | 1 | SMD, 0.32 (0.05, 0.58) | 239 | 93 | 146 | 0.018 | NA | NA | NA | No | Yes | 1.78 | IV |
| Primary neurological abnormalities | 1 | SMD, 0.29 (0.03, 0.55) | 239 | 93 | 146 | 0.03 | NA | NA | NA | No | Yes | 1.69 | IV |
| Stage 1 % | 24 | SMD, 0.26 (0.06, 0.46) | 809 | 396 | 413 | 0.012 | 41% | -0.41, 0.93 | No | Yes | No | 1.59 | IV |
| Stage 3 % | 10 | SMD, -0.19 (-0.37, -0.01) | 491 | 247 | 244 | 0.04 | 0% | -0.4, 0.02 | No | No | No | 0.71 | IV |
| Total A-B ridge count (TABRC) | 14 | SMD, -0.17 (-0.29, -0.04) | 2021 | 1031 | 990 | 0.0091 | 46% | -0.53, 0.19 | No | Yes | No | 0.74 | IV |
| Early morning awake time | 2 | SMD, 1.24 (-0.23, 2.71) | 59 | 28 | 31 | 0.099 | 82% | NA | NA | No | No | 9.45 | ns |
| D-serine (Serum) | 6 | SMD, -1.05 (-2.35, 0.26) | 606 | 320 | 286 | 0.12 | 95% | -5.89, 3.8 | Yes | Yes | Yes | 0.15 | ns |
| Symbolic dynamic measures of EEG-F4 Awake (word count) | 1 | SMD, -0.95 (-1.94, 0.04) | 18 | 9 | 9 | 0.059 | NA | NA | NA | No | Yes | 0.18 | ns |
| Symbolic dynamic measures of EEG-F4 REM (word count) | 1 | SMD, -0.95 (-1.94, 0.04) | 18 | 9 | 9 | 0.059 | NA | NA | NA | No | Yes | 0.18 | ns |
| Symbolic dynamic measures of EEG-F4 Stage 1/2 (SD of the word sequence) | 1 | SMD, -0.94 (-1.93, 0.04) | 18 | 9 | 9 | 0.061 | NA | NA | NA | No | No | 0.18 | ns |
| Symbolic dynamic measures of EEG-F4 Stage 1/2 (word count) | 1 | SMD, -0.88 (-1.86, 0.1) | 18 | 9 | 9 | 0.077 | NA | NA | NA | No | No | 0.20 | ns |
| Graphaesthesia L | 2 | OR, 4.9 (0.56, 42.86) | 107 | 60 | 47 | 0.15 | 0% | NA | NA | No | No | 4.90 | ns |
| Delta half-wave count per hour in third sleep cycle | 2 | SMD, -0.85 (-1.7, 0) | 24 | 12 | 12 | 0.05 | 0% | NA | NA | No | No | 0.21 | ns |
| Sleep efficiency % 1st night | 1 | SMD, -0.87 (-1.79, 0.06) | 20 | 10 | 10 | 0.066 | NA | NA | NA | No | No | 0.21 | ns |
| Sleep efficiency % 2nd night | 1 | SMD, -0.87 (-1.79, 0.06) | 20 | 10 | 10 | 0.066 | NA | NA | NA | No | No | 0.21 | ns |
| Nonlinearity scores of EEG-F4 Awake | 1 | SMD, -0.86 (-1.84, 0.11) | 18 | 9 | 9 | 0.084 | NA | NA | NA | No | No | 0.21 | ns |
| Symbolic dynamic measures of EEG-C4 Awake (SD of the word sequence) | 1 | SMD, -0.83 (-1.81, 0.14) | 18 | 9 | 9 | 0.093 | NA | NA | NA | No | No | 0.22 | ns |
| Frequency (Hz) delta half-wave count per hour 1.5 -2.1 | 2 | SMD, -0.8 (-1.65, 0.05) | 24 | 12 | 12 | 0.064 | 0% | NA | NA | No | No | 0.23 | ns |
| Sleep efficiency % 3rd night | 1 | SMD, -0.82 (-1.74, 0.1) | 20 | 10 | 10 | 0.081 | NA | NA | NA | No | No | 0.23 | ns |
| REM sleep without atonia % | 1 | SMD, 0.8 (-0.12, 1.72) | 20 | 10 | 10 | 0.089 | NA | NA | NA | No | No | 4.25 | ns |
| Frequency (Hz) delta half-wave count per hour 1.0 -1.5 | 2 | SMD, -0.79 (-1.64, 0.06) | 24 | 12 | 12 | 0.068 | 0% | NA | NA | No | No | 0.24 | ns |
| Symbolic dynamic measures of EEG-C4 Stage 1/2 (word count) | 1 | SMD, -0.78 (-1.74, 0.19) | 18 | 9 | 9 | 0.12 | NA | NA | NA | No | No | 0.24 | ns |
| REM % 1st night | 1 | SMD, 0.78 (-0.14, 1.7) | 20 | 10 | 10 | 0.095 | NA | NA | NA | No | No | 4.13 | ns |
| Sleep latency min 1st night | 1 | SMD, 0.75 (-0.17, 1.66) | 20 | 10 | 10 | 0.11 | NA | NA | NA | No | No | 3.89 | ns |
| Stage 3 Amplitude | 1 | SMD, -0.74 (-1.54, 0.06) | 26 | 13 | 13 | 0.068 | NA | NA | NA | No | No | 0.26 | ns |
| REM time (age adjusted) | 1 | SMD, -0.74 (-1.62, 0.14) | 24 | 8 | 16 | 0.1 | NA | NA | NA | No | No | 0.26 | ns |
| S2q4 fast sigma power C4 % | 1 | SMD, -0.74 (-1.5, 0.02) | 29 | 14 | 15 | 0.055 | NA | NA | NA | No | No | 0.26 | ns |
| S2q4 spindle density C4 min-1 | 1 | SMD, -0.75 (-1.51, 0.01) | 29 | 14 | 15 | 0.052 | NA | NA | NA | No | Yes | 0.26 | ns |
| Symbolic dynamic measures of EEG-F4 Stage 3/4 (SD of the word sequence) | 1 | SMD, -0.74 (-1.7, 0.23) | 18 | 9 | 9 | 0.13 | NA | NA | NA | No | No | 0.26 | ns |
| Fluctuating asymmetry A–B ridge count (FABRC) | 4 | SMD, 0.74 (-0.65, 2.14) | 539 | 241 | 298 | 0.3 | 98% | -6.02, 7.51 | No | No | Yes | 3.84 | ns |
| Sleep latency min 3rd night | 1 | SMD, 0.72 (-0.19, 1.64) | 20 | 10 | 10 | 0.12 | NA | NA | NA | No | No | 3.72 | ns |
| Intermediate Sleep scored "at the expense" of REM sleep % | 1 | SMD, 0.72 (-0.19, 1.63) | 20 | 10 | 10 | 0.12 | NA | NA | NA | No | No | 3.71 | ns |
| Sigma (11.4-16.7) | 2 | SMD, 0.72 (-0.12, 1.56) | 24 | 12 | 12 | 0.092 | 0% | NA | NA | No | No | 3.69 | ns |
| Snout reflex | 2 | OR, 3.67 (0.38, 35.12) | 107 | 60 | 47 | 0.26 | 0% | NA | NA | No | No | 3.67 | ns |
| The fast component CoP-CoM cm, eyes open on firm surface, antero-posterior | 1 | SMD, 0.71 (-0.07, 1.49) | 29 | 11 | 18 | 0.073 | NA | NA | NA | No | No | 3.62 | ns |
| REM sleep without rapid eye movement % | 1 | SMD, 0.7 (-0.21, 1.61) | 20 | 10 | 10 | 0.13 | NA | NA | NA | No | No | 3.57 | ns |
| Total Dissociated Stages of Sleep % | 1 | SMD, 0.7 (-0.21, 1.61) | 20 | 10 | 10 | 0.13 | NA | NA | NA | No | No | 3.54 | ns |
| First REM duration (1st night) | 1 | SMD, 0.69 (-0.22, 1.6) | 20 | 10 | 10 | 0.14 | NA | NA | NA | No | No | 3.48 | ns |
| The slow component CoM cm, eyes open on firm surface, antero-posterior | 1 | SMD, 0.69 (-0.09, 1.46) | 29 | 11 | 18 | 0.082 | NA | NA | NA | No | No | 3.48 | ns |
| The fast component CoP-CoM cm, eyes open on yielding surface, medio-lateral | 1 | SMD, 0.68 (-0.09, 1.46) | 29 | 11 | 18 | 0.083 | NA | NA | NA | No | No | 3.46 | ns |
| Stage 2 % 1st night | 1 | SMD, -0.69 (-1.6, 0.22) | 20 | 10 | 10 | 0.14 | NA | NA | NA | No | No | 0.29 | ns |
| Stage 2 % 3rd night | 1 | SMD, -0.68 (-1.59, 0.22) | 20 | 10 | 10 | 0.14 | NA | NA | NA | No | No | 0.29 | ns |
| Lateral Shift during eye closure cm | 1 | SMD, -0.69 (-1.38, 0) | 36 | 22 | 14 | 0.052 | NA | NA | NA | No | Yes | 0.29 | ns |
| The slow component CoM cm, eyes open on yielding surface, medio-lateral | 1 | SMD, 0.68 (-0.09, 1.45) | 29 | 11 | 18 | 0.085 | NA | NA | NA | No | No | 3.43 | ns |
| The fast component CoP-CoM cm, eyes open on yielding surface, antero-posterior | 1 | SMD, 0.67 (-0.1, 1.44) | 29 | 11 | 18 | 0.088 | NA | NA | NA | No | No | 3.39 | ns |
| Slow wave sleep latency | 2 | SMD, 0.66 (-0.09, 1.42) | 72 | 37 | 35 | 0.086 | 58% | NA | NA | No | No | 3.32 | ns |
| Sleep latency min averaged night 2+3 | 1 | SMD, 0.66 (-0.25, 1.56) | 20 | 10 | 10 | 0.15 | NA | NA | NA | No | No | 3.30 | ns |
| The fast component CoP-CoM cm, eyes open on firm surface, medio-lateral | 1 | SMD, 0.66 (-0.11, 1.43) | 29 | 11 | 18 | 0.095 | NA | NA | NA | No | No | 3.30 | ns |
| Stage 3 % 2nd night | 1 | SMD, -0.64 (-1.54, 0.27) | 20 | 10 | 10 | 0.17 | NA | NA | NA | No | No | 0.31 | ns |
| Frequency (Hz) delta half-wave count per hour 0.33-0.5 | 2 | SMD, 0.64 (-0.19, 1.47) | 24 | 12 | 12 | 0.13 | 0% | NA | NA | No | No | 3.19 | ns |
| Relative beta2 spectral amplitude uV in frontal region | 1 | SMD, 0.64 (-0.09, 1.37) | 40 | 10 | 30 | 0.087 | NA | NA | NA | No | No | 3.18 | ns |
| Nonlinearity scores of EEG-C4 Stage 3/4 | 1 | SMD, 0.63 (-0.32, 1.58) | 18 | 9 | 9 | 0.19 | NA | NA | NA | No | No | 3.14 | ns |
| Largest Lyapunov exponent of EEG-C4 Awake | 1 | SMD, -0.63 (-1.58, 0.33) | 18 | 9 | 9 | 0.2 | NA | NA | NA | No | No | 0.32 | ns |
| Intermediate Sleep % | 1 | SMD, 0.62 (-0.28, 1.53) | 20 | 10 | 10 | 0.18 | NA | NA | NA | No | No | 3.10 | ns |
| Beta (16.0-30.0) | 2 | SMD, 0.62 (-0.21, 1.45) | 24 | 12 | 12 | 0.14 | 0% | NA | NA | No | No | 3.08 | ns |
| Stage 2 Amplitude | 1 | SMD, -0.62 (-1.41, 0.17) | 26 | 13 | 13 | 0.13 | NA | NA | NA | No | No | 0.33 | ns |
| Relative beta2 spectral amplitude uV in temporal region | 1 | SMD, 0.6 (-0.13, 1.33) | 40 | 10 | 30 | 0.11 | NA | NA | NA | No | No | 2.98 | ns |
| Nonlinearity scores of EEG-F4 Stage 1/2 | 1 | SMD, 0.6 (-0.35, 1.55) | 18 | 9 | 9 | 0.21 | NA | NA | NA | No | No | 2.98 | ns |
| Relative abundance of Neisseria_sicca in oropharynx | 1 | SMD, 0.6 (-0.11, 1.31) | 32 | 16 | 16 | 0.097 | NA | NA | NA | No | Yes | 2.97 | ns |
| Awake time 1st sleep cycle %NREM period | 1 | SMD, 0.6 (-0.02, 1.22) | 42 | 22 | 20 | 0.059 | NA | NA | NA | No | No | 2.95 | ns |
| REM number (3rd night) | 1 | SMD, -0.59 (-1.49, 0.31) | 20 | 10 | 10 | 0.2 | NA | NA | NA | No | No | 0.34 | ns |
| Largest Lyapunov exponent of EEG-C4 Stage 3/4 | 1 | SMD, -0.6 (-1.55, 0.35) | 18 | 9 | 9 | 0.21 | NA | NA | NA | No | No | 0.34 | ns |
| First REM duration (2nd night) | 1 | SMD, 0.58 (-0.31, 1.48) | 20 | 10 | 10 | 0.2 | NA | NA | NA | No | No | 2.89 | ns |
| Frequency (Hz) delta half-wave count per hour 2.1 -2.6 | 2 | SMD, -0.58 (-1.4, 0.25) | 24 | 12 | 12 | 0.17 | 0% | NA | NA | No | No | 0.35 | ns |
| Stage 1 Incidence | 1 | SMD, -0.58 (-1.37, 0.21) | 26 | 13 | 13 | 0.15 | NA | NA | NA | No | No | 0.35 | ns |
| Theta (2.5-7.1) | 2 | SMD, -0.57 (-1.4, 0.25) | 24 | 12 | 12 | 0.17 | 0% | NA | NA | No | No | 0.35 | ns |
| Total sleep time, min | 1 | SMD, -0.58 (-1.48, 0.32) | 20 | 10 | 10 | 0.21 | NA | NA | NA | No | No | 0.35 | ns |
| Total sleep time min | 1 | SMD, -0.58 (-1.23, 0.07) | 40 | 15 | 25 | 0.082 | NA | NA | NA | No | No | 0.35 | ns |
| AIMS total | 1 | SMD, 0.57 (-0.03, 1.18) | 44 | 22 | 22 | 0.064 | NA | NA | NA | No | No | 2.82 | ns |
| Relative beta2 spectral amplitude uV in central region | 1 | SMD, 0.57 (-0.16, 1.3) | 40 | 10 | 30 | 0.13 | NA | NA | NA | No | No | 2.80 | ns |
| Relative spectral amplitude uV, alpha | 1 | SMD, -0.56 (-1.29, 0.17) | 40 | 10 | 30 | 0.13 | NA | NA | NA | No | No | 0.36 | ns |
| Stage shifts | 1 | SMD, -0.56 (-1.19, 0.08) | 40 | 20 | 20 | 0.085 | NA | NA | NA | No | No | 0.36 | ns |
| Spindles duration s | 1 | SMD, -0.56 (-1.22, 0.09) | 40 | 15 | 25 | 0.09 | NA | NA | NA | No | No | 0.36 | ns |
| Relative abundance of Neisseria_meningitidis in oropharynx | 1 | SMD, 0.55 (-0.16, 1.26) | 32 | 16 | 16 | 0.13 | NA | NA | NA | No | Yes | 2.71 | ns |
| Slow wave sleep 2nd sleep cycle %NREM period | 1 | SMD, -0.54 (-1.16, 0.08) | 42 | 22 | 20 | 0.086 | NA | NA | NA | No | No | 0.37 | ns |
| Stage 2 % averaged night 2+3 | 1 | SMD, -0.55 (-1.45, 0.34) | 20 | 10 | 10 | 0.23 | NA | NA | NA | No | No | 0.37 | ns |
| Spectral Power during Stage 2 sleep µV2/Hz - Slow (0.5-1Hz) | 1 | SMD, -0.55 (-1.21, 0.1) | 40 | 15 | 25 | 0.096 | NA | NA | NA | No | No | 0.37 | ns |
| Stage 1 % (age adjusted) | 1 | SMD, 0.53 (-0.33, 1.4) | 24 | 8 | 16 | 0.23 | NA | NA | NA | No | No | 2.64 | ns |
| Stage 3 Incidence | 1 | SMD, -0.53 (-1.32, 0.25) | 26 | 13 | 13 | 0.18 | NA | NA | NA | No | No | 0.38 | ns |
| Spectral Power during Stage 2 sleep µV2/Hz - Alpha (8-12Hz) | 1 | SMD, -0.54 (-1.19, 0.12) | 40 | 15 | 25 | 0.11 | NA | NA | NA | No | No | 0.38 | ns |
| Mean power frequency Hz, eyes open on yielding surface, antero-posterior | 1 | SMD, 0.53 (-0.23, 1.3) | 29 | 11 | 18 | 0.17 | NA | NA | NA | No | No | 2.63 | ns |
| REM sleep efficiency % | 1 | SMD, 0.52 (-0.37, 1.42) | 20 | 10 | 10 | 0.25 | NA | NA | NA | No | No | 2.59 | ns |
| Center Point of Force Velocity during eyes open cm/s | 1 | SMD, 0.52 (-0.16, 1.2) | 36 | 22 | 14 | 0.13 | NA | NA | NA | No | No | 2.58 | ns |
| Sleep latency min 2nd night | 1 | SMD, 0.52 (-0.38, 1.41) | 20 | 10 | 10 | 0.26 | NA | NA | NA | No | No | 2.55 | ns |
| Awake time % 2nd night | 1 | SMD, 0.52 (-0.38, 1.41) | 20 | 10 | 10 | 0.26 | NA | NA | NA | No | No | 2.55 | ns |
| Awakenings number (2nd night) | 1 | SMD, 0.51 (-0.38, 1.4) | 20 | 10 | 10 | 0.26 | NA | NA | NA | No | No | 2.52 | ns |
| Intra-REM sleep periods Intermediate Sleep % | 1 | SMD, 0.5 (-0.39, 1.4) | 20 | 10 | 10 | 0.27 | NA | NA | NA | No | No | 2.49 | ns |
| Awakenings number (averaged night 2+3) | 1 | SMD, 0.49 (-0.4, 1.39) | 20 | 10 | 10 | 0.28 | NA | NA | NA | No | No | 2.45 | ns |
| Mean power frequency Hz, eyes open on firm surface, antero-posterior | 1 | SMD, 0.49 (-0.27, 1.25) | 29 | 11 | 18 | 0.21 | NA | NA | NA | No | No | 2.44 | ns |
| First REM density (1st night) | 1 | SMD, -0.49 (-1.38, 0.41) | 20 | 10 | 10 | 0.29 | NA | NA | NA | No | No | 0.41 | ns |
| REM latency + Biperiden | 1 | SMD, -0.48 (-1.33, 0.38) | 22 | 12 | 10 | 0.27 | NA | NA | NA | No | No | 0.42 | ns |
| Stage 1 % 3rd night | 1 | SMD, 0.48 (-0.42, 1.37) | 20 | 10 | 10 | 0.3 | NA | NA | NA | No | No | 2.37 | ns |
| Relative abundance of Haemophilus_influenzae in oropharynx | 1 | SMD, 0.47 (-0.23, 1.18) | 32 | 16 | 16 | 0.19 | NA | NA | NA | No | Yes | 2.37 | ns |
| Position 2 sway in the medial-lateral direction cm | 1 | SMD, 0.47 (0, 0.93) | 72 | 36 | 36 | 0.051 | NA | NA | NA | No | Yes | 2.33 | ns |
| Position 3 sway in the medial-lateral direction cm | 1 | SMD, 0.47 (0, 0.93) | 72 | 36 | 36 | 0.051 | NA | NA | NA | No | Yes | 2.33 | ns |
| Position 5 sway in the medial-lateral direction cm | 1 | SMD, 0.47 (0, 0.93) | 72 | 36 | 36 | 0.051 | NA | NA | NA | No | Yes | 2.33 | ns |
| Position 2 mean sway cm | 1 | SMD, 0.47 (0, 0.93) | 72 | 36 | 36 | 0.051 | NA | NA | NA | No | Yes | 2.33 | ns |
| The slow component CoM cm, eyes closed on firm surface, medio-lateral | 1 | SMD, 0.46 (-0.3, 1.22) | 29 | 11 | 18 | 0.23 | NA | NA | NA | No | No | 2.31 | ns |
| Slow wave sleep duration | 5 | SMD, -0.45 (-1.18, 0.28) | 89 | 44 | 45 | 0.23 | 64% | -2.82, 1.92 | No | Yes | Yes | 0.44 | ns |
| D-serine (brain tissue) Parietal cortex | 2 | SMD, -0.45 (-1.03, 0.12) | 48 | 24 | 24 | 0.12 | 0% | NA | NA | No | No | 0.44 | ns |
| Symbolic dynamic measures of EEG-F4 Awake (SD of the word sequence) | 1 | SMD, -0.46 (-1.4, 0.48) | 18 | 9 | 9 | 0.34 | NA | NA | NA | No | No | 0.44 | ns |
| Sequencing of motor complex acts (NES) | 2 | SMD, 0.45 (-0.07, 0.97) | 66 | 44 | 22 | 0.091 | 0% | NA | NA | No | No | 2.25 | ns |
| Position 10 sway in the anterior-posterior direction cm | 1 | SMD, 0.45 (-0.02, 0.91) | 72 | 36 | 36 | 0.062 | NA | NA | NA | No | No | 2.24 | ns |
| REM number (averaged night 2+3) | 1 | SMD, -0.44 (-1.33, 0.45) | 20 | 10 | 10 | 0.33 | NA | NA | NA | No | No | 0.45 | ns |
| REM latency averaged night 2+3 | 1 | SMD, -0.44 (-1.33, 0.45) | 20 | 10 | 10 | 0.33 | NA | NA | NA | No | No | 0.45 | ns |
| Sleep maintenance % | 7 | SMD, -0.44 (-1.04, 0.16) | 209 | 100 | 109 | 0.15 | 68% | -2.28, 1.4 | No | Yes | No | 0.45 | ns |
| Stage 1 shift | 1 | SMD, -0.44 (-1.07, 0.18) | 40 | 20 | 20 | 0.17 | NA | NA | NA | No | No | 0.45 | ns |
| REM % (age adjusted) | 1 | SMD, -0.43 (-1.29, 0.42) | 24 | 8 | 16 | 0.32 | NA | NA | NA | No | No | 0.45 | ns |
| D-serine (brain tissue) Prefrontal cortex | 1 | SMD, -0.44 (-1.75, 0.86) | 13 | 3 | 10 | 0.51 | NA | NA | NA | No | No | 0.45 | ns |
| Symbolic dynamic measures of EEG-F4 Stage 3/4 (word count) | 1 | SMD, -0.44 (-1.37, 0.5) | 18 | 9 | 9 | 0.36 | NA | NA | NA | No | No | 0.45 | ns |
| Anterior-posterior shift during eye closure cm | 1 | SMD, -0.45 (-1.12, 0.23) | 36 | 22 | 14 | 0.2 | NA | NA | NA | No | No | 0.45 | ns |
| Awakening | 1 | SMD, 0.44 (-0.19, 1.07) | 40 | 20 | 20 | 0.17 | NA | NA | NA | No | No | 2.22 | ns |
| Awakenings number | 9 | SMD, 0.44 (-0.12, 0.99) | 219 | 107 | 112 | 0.12 | 67% | -1.34, 2.21 | Yes | No | Yes | 2.21 | ns |
| Mean power frequency Hz, eyes open on firm surface | 1 | SMD, 0.43 (-0.33, 1.19) | 29 | 11 | 18 | 0.27 | NA | NA | NA | No | No | 2.18 | ns |
| Largest Lyapunov exponent of EEG-F4 Awake | 1 | SMD, -0.43 (-1.36, 0.51) | 18 | 9 | 9 | 0.37 | NA | NA | NA | No | No | 0.46 | ns |
| Position 1 mean sway cm | 1 | SMD, 0.43 (-0.04, 0.89) | 72 | 36 | 36 | 0.074 | NA | NA | NA | No | No | 2.17 | ns |
| First REM density | 4 | SMD, 0.42 (-0.08, 0.93) | 131 | 65 | 66 | 0.1 | 49% | -1.48, 2.33 | No | Yes | No | 2.16 | ns |
| Relative abundance of Aggregatibacter_aphrophilus in oropharynx | 1 | SMD, 0.42 (-0.28, 1.12) | 32 | 16 | 16 | 0.24 | NA | NA | NA | No | Yes | 2.13 | ns |
| First REM density 2nd night (age adjusted) | 1 | SMD, -0.41 (-1.27, 0.44) | 24 | 8 | 16 | 0.34 | NA | NA | NA | No | No | 0.47 | ns |
| Slow wave sleep 1st sleep cycle %NREM period | 1 | SMD, -0.42 (-1.03, 0.19) | 42 | 22 | 20 | 0.18 | NA | NA | NA | No | No | 0.47 | ns |
| Rapid eye movement density during REM sleep | 1 | SMD, 0.41 (-0.33, 1.15) | 29 | 14 | 15 | 0.28 | NA | NA | NA | No | No | 2.10 | ns |
| Position 1 sway in the medial-lateral direction cm | 1 | SMD, 0.41 (-0.06, 0.88) | 72 | 36 | 36 | 0.087 | NA | NA | NA | No | No | 2.10 | ns |
| The fast component CoP-CoM cm, eyes closed on firm surface, medio-lateral | 1 | SMD, 0.41 (-0.35, 1.16) | 29 | 11 | 18 | 0.29 | NA | NA | NA | No | No | 2.09 | ns |
| Second REM duration | 1 | SMD, -0.41 (-1.13, 0.31) | 30 | 15 | 15 | 0.27 | NA | NA | NA | No | No | 0.48 | ns |
| Stage 2 % (age adjusted) | 1 | SMD, -0.4 (-1.26, 0.46) | 24 | 8 | 16 | 0.36 | NA | NA | NA | No | No | 0.48 | ns |
| Symbolic dynamic measures of EEG-C4 Stage 3/4 (word count) | 1 | SMD, -0.4 (-1.34, 0.53) | 18 | 9 | 9 | 0.4 | NA | NA | NA | No | No | 0.48 | ns |
| Position 9 sway in the medial-lateral direction cm | 1 | SMD, 0.4 (-0.06, 0.87) | 72 | 36 | 36 | 0.09 | NA | NA | NA | No | No | 2.08 | ns |
| Sensory integration (NES) | 1 | SMD, 0.4 (-0.2, 0.99) | 44 | 22 | 22 | 0.19 | NA | NA | NA | No | No | 2.05 | ns |
| Executive function: Wisconsin Card Sorting Test—categories completed (WCC) | 1 | SMD, -0.38 (-0.85, 0.09) | 73 | 43 | 30 | 0.11 | NA | NA | NA | No | No | 0.50 | ns |
| REM latency 3rd night | 1 | SMD, -0.39 (-1.27, 0.5) | 20 | 10 | 10 | 0.39 | NA | NA | NA | No | No | 0.50 | ns |
| REM density (second sleep cycle) | 1 | SMD, -0.38 (-0.99, 0.23) | 42 | 22 | 20 | 0.22 | NA | NA | NA | No | No | 0.50 | ns |
| Stages 3 and 4 % | 9 | SMD, -0.39 (-1.07, 0.3) | 220 | 102 | 118 | 0.27 | 77% | -2.71, 1.94 | No | Yes | No | 0.50 | ns |
| Awake time % averaged night 2+3 | 1 | SMD, 0.38 (-0.51, 1.27) | 20 | 10 | 10 | 0.4 | NA | NA | NA | No | No | 2.00 | ns |
| The slow component CoM cm, eyes open on firm surface, medio-lateral | 1 | SMD, 0.38 (-0.38, 1.14) | 29 | 11 | 18 | 0.33 | NA | NA | NA | No | No | 1.99 | ns |
| Executive function: Wisconsin Card Sorting Test—total perseverative errors (WTPE) | 1 | SMD, 0.38 (-0.09, 0.85) | 73 | 43 | 30 | 0.12 | NA | NA | NA | No | No | 1.98 | ns |
| REM density 2nd night | 1 | SMD, 0.38 (-0.51, 1.26) | 20 | 10 | 10 | 0.4 | NA | NA | NA | No | No | 1.98 | ns |
| Intermediate Sleep scored "at the expense" of REM sleep % of REM sleep duration | 1 | SMD, 0.38 (-0.51, 1.26) | 20 | 10 | 10 | 0.4 | NA | NA | NA | No | No | 1.98 | ns |
| Nonlinearity scores of EEG-F4 Stage 3/4 | 1 | SMD, 0.37 (-0.56, 1.31) | 18 | 9 | 9 | 0.43 | NA | NA | NA | No | No | 1.97 | ns |
| Saccade target | 2 | OR, 0.51 (0.04, 5.75) | 107 | 60 | 47 | 0.58 | 0% | NA | NA | No | No | 0.51 | ns |
| Saccade command | 2 | OR, 0.51 (0.04, 5.75) | 107 | 60 | 47 | 0.58 | 0% | NA | NA | No | No | 0.51 | ns |
| Grasp reflex R | 2 | OR, 0.51 (0.04, 5.75) | 107 | 60 | 47 | 0.58 | 0% | NA | NA | No | No | 0.51 | ns |
| Grasp reflex L | 2 | OR, 0.51 (0.04, 5.75) | 107 | 60 | 47 | 0.58 | 0% | NA | NA | No | No | 0.51 | ns |
| Stereognosis R | 2 | OR, 0.51 (0.04, 5.75) | 107 | 60 | 47 | 0.58 | 0% | NA | NA | No | No | 0.51 | ns |
| Rapid alternate movements R | 2 | OR, 0.51 (0.04, 5.75) | 107 | 60 | 47 | 0.58 | 0% | NA | NA | No | No | 0.51 | ns |
| Rapid alternate movements L | 2 | OR, 0.51 (0.04, 5.75) | 107 | 60 | 47 | 0.58 | 0% | NA | NA | No | No | 0.51 | ns |
| Slow-wave sleep (SWS) (stages 3+4) % | 1 | SMD, -0.37 (-1.26, 0.51) | 20 | 10 | 10 | 0.41 | NA | NA | NA | No | No | 0.51 | ns |
| Subjects with REM latency<65 min | 1 | OR, 0.52 (0.1, 2.58) | 26 | 13 | 13 | 0.42 | NA | NA | NA | No | No | 0.52 | ns |
| REM sleep % | 1 | SMD, 0.36 (-0.52, 1.25) | 20 | 10 | 10 | 0.42 | NA | NA | NA | No | No | 1.92 | ns |
| Position 3 sway in the anterior-posterior direction cm | 1 | SMD, 0.36 (-0.11, 0.82) | 72 | 36 | 36 | 0.13 | NA | NA | NA | No | No | 1.91 | ns |
| Position 6 sway in the anterior-posterior direction cm | 1 | SMD, 0.36 (-0.11, 0.82) | 72 | 36 | 36 | 0.13 | NA | NA | NA | No | No | 1.91 | ns |
| The slow component CoM cm, eyes closed on firm surface, antero-posterior | 1 | SMD, 0.36 (-0.4, 1.11) | 29 | 11 | 18 | 0.35 | NA | NA | NA | No | No | 1.91 | ns |
| Awakenings number (3rd night) | 1 | SMD, 0.35 (-0.53, 1.24) | 20 | 10 | 10 | 0.43 | NA | NA | NA | No | No | 1.90 | ns |
| Position 7 sway in the anterior-posterior direction cm | 1 | SMD, 0.35 (-0.11, 0.82) | 72 | 36 | 36 | 0.14 | NA | NA | NA | No | No | 1.90 | ns |
| High sigma µV2/Hz | 1 | SMD, -0.35 (-1, 0.29) | 38 | 21 | 17 | 0.28 | NA | NA | NA | No | No | 0.53 | ns |
| Relative spectral amplitude uV, theta | 1 | SMD, -0.35 (-1.07, 0.37) | 40 | 10 | 30 | 0.34 | NA | NA | NA | No | No | 0.53 | ns |
| Slow wave sleep % | 10 | SMD, -0.35 (-0.82, 0.12) | 257 | 126 | 131 | 0.14 | 69% | -1.86, 1.16 | No | No | No | 0.53 | ns |
| Stage 2 latency | 2 | SMD, 0.35 (-0.14, 0.83) | 66 | 33 | 33 | 0.16 | 0% | NA | NA | No | No | 1.88 | ns |
| Electroencephalographically-mixed Intermediate Sleep % | 1 | SMD, 0.35 (-0.54, 1.23) | 20 | 10 | 10 | 0.44 | NA | NA | NA | No | No | 1.87 | ns |
| Symbolic dynamic measures of EEG-C4 Stage 3/4 (SD of the word sequence) | 1 | SMD, 0.34 (-0.59, 1.28) | 18 | 9 | 9 | 0.47 | NA | NA | NA | No | No | 1.87 | ns |
| R/L confusion | 2 | OR, 1.86 (0.68, 5.08) | 107 | 60 | 47 | 0.23 | 0% | NA | NA | No | No | 1.86 | ns |
| Awake time % | 4 | SMD, 0.34 (-0.18, 0.86) | 247 | 124 | 123 | 0.2 | 64% | -1.82, 2.5 | No | No | No | 1.86 | ns |
| Second REM density | 1 | SMD, 0.34 (-0.38, 1.06) | 30 | 15 | 15 | 0.35 | NA | NA | NA | No | No | 1.86 | ns |
| Blood anti-C. albicans Immunoglobulin G (IgG) level Candida albicans | 2 | OR, 1.86 (0.64, 5.38) | 538 | 261 | 277 | 0.25 | 81% | NA | NA | No | No | 1.86 | ns |
| REM activity (units) (age adjusted) | 1 | SMD, -0.34 (-1.19, 0.52) | 24 | 8 | 16 | 0.44 | NA | NA | NA | No | No | 0.54 | ns |
| REM latency (-wake time) | 1 | SMD, -0.33 (-0.95, 0.28) | 42 | 22 | 20 | 0.28 | NA | NA | NA | No | No | 0.54 | ns |
| Stages 3 and 4 duration | 2 | SMD, -0.34 (-0.87, 0.19) | 59 | 28 | 31 | 0.21 | 0% | NA | NA | No | No | 0.54 | ns |
| Stage 3 % averaged night 2+3 | 1 | SMD, -0.33 (-1.22, 0.55) | 20 | 10 | 10 | 0.46 | NA | NA | NA | No | No | 0.54 | ns |
| REMS density Nb/minute REM sleep | 1 | SMD, -0.34 (-1.22, 0.55) | 20 | 10 | 10 | 0.46 | NA | NA | NA | No | No | 0.54 | ns |
| Frequency (Hz) delta half-wave count per hour 0.5 - 1.0 | 2 | SMD, 0.33 (-0.48, 1.14) | 24 | 12 | 12 | 0.42 | 0% | NA | NA | No | No | 1.82 | ns |
| REM % 3rd night | 1 | SMD, 0.33 (-0.55, 1.21) | 20 | 10 | 10 | 0.46 | NA | NA | NA | No | No | 1.82 | ns |
| D-serine (CSF) | 3 | SMD, -0.32 (-0.89, 0.25) | 102 | 56 | 46 | 0.27 | 49% | -6.1, 5.46 | Yes | No | No | 0.56 | ns |
| NREM sleep with rapid eye movement % | 1 | SMD, -0.32 (-1.2, 0.57) | 20 | 10 | 10 | 0.48 | NA | NA | NA | No | No | 0.56 | ns |
| Finger–thumb opposition L | 2 | OR, 1.78 (0.18, 17.36) | 107 | 60 | 47 | 0.62 | 0% | NA | NA | No | No | 1.78 | ns |
| Mean power frequency Hz, eyes closed on firm surface, medio-lateral | 1 | SMD, 0.31 (-0.44, 1.07) | 29 | 11 | 18 | 0.42 | NA | NA | NA | No | No | 1.76 | ns |
| Fluctuating asymmetry finger ridge count (FAFRC) | 4 | SMD, 0.31 (-0.43, 1.05) | 531 | 233 | 298 | 0.41 | 94% | -3.2, 3.82 | No | Yes | Yes | 1.76 | ns |
| S2q4 fast sigma power C4–C3 % | 1 | SMD, -0.31 (-1.05, 0.42) | 29 | 14 | 15 | 0.4 | NA | NA | NA | No | No | 0.57 | ns |
| REM % averaged night 2+3 | 1 | SMD, 0.3 (-0.58, 1.19) | 20 | 10 | 10 | 0.5 | NA | NA | NA | No | No | 1.74 | ns |
| First REM activity (units) | 2 | SMD, 0.3 (-0.23, 0.83) | 59 | 28 | 31 | 0.27 | 0% | NA | NA | No | No | 1.73 | ns |
| Stage 2 % 2nd night | 1 | SMD, -0.3 (-1.18, 0.58) | 20 | 10 | 10 | 0.51 | NA | NA | NA | No | No | 0.58 | ns |
| Position 1 sway in the anterior-posterior direction cm | 1 | SMD, 0.29 (-0.17, 0.76) | 72 | 36 | 36 | 0.22 | NA | NA | NA | No | No | 1.70 | ns |
| First REM density 1st night (age adjusted) | 1 | SMD, 0.28 (-0.58, 1.13) | 24 | 8 | 16 | 0.53 | NA | NA | NA | No | No | 1.65 | ns |
| Center Point of Force Velocity during eyes closed cm/s | 1 | SMD, -0.27 (-0.95, 0.4) | 36 | 22 | 14 | 0.43 | NA | NA | NA | No | No | 0.61 | ns |
| Finger-thumb tapping left | 1 | SMD, 0.27 (-0.05, 0.58) | 161 | 68 | 93 | 0.095 | NA | NA | NA | No | No | 1.62 | ns |
| No of finger taps per 10 s, non-preferred hand, occasion 2 | 1 | SMD, 0.27 (-0.15, 0.68) | 92 | 37 | 55 | 0.21 | NA | NA | NA | No | No | 1.62 | ns |
| Position 2 sway in the anterior-posterior direction cm | 1 | SMD, 0.26 (-0.2, 0.73) | 72 | 36 | 36 | 0.26 | NA | NA | NA | No | No | 1.62 | ns |
| Position 7 mean sway cm | 1 | SMD, 0.26 (-0.2, 0.73) | 72 | 36 | 36 | 0.26 | NA | NA | NA | No | No | 1.62 | ns |
| Awakenings number (1st night) | 1 | SMD, -0.26 (-1.14, 0.62) | 20 | 10 | 10 | 0.56 | NA | NA | NA | No | No | 0.62 | ns |
| NREM duration (second sleep cycle) | 1 | SMD, -0.26 (-0.87, 0.35) | 42 | 22 | 20 | 0.4 | NA | NA | NA | No | No | 0.62 | ns |
| Mirror movements L | 2 | OR, 1.59 (0.23, 10.95) | 107 | 60 | 47 | 0.64 | 0% | NA | NA | No | No | 1.59 | ns |
| Fingertip patter asymetry-three pattern classification | 5 | SMD, 0.25 (-0.08, 0.59) | 476 | 249 | 227 | 0.14 | 66% | -0.86, 1.37 | No | No | Yes | 1.59 | ns |
| No of finger taps per 10 s, preferred hand, occasion 2 | 1 | SMD, -0.25 (-0.67, 0.17) | 92 | 37 | 55 | 0.25 | NA | NA | NA | No | No | 0.64 | ns |
| First REM duration (3rd night) | 1 | SMD, -0.25 (-1.13, 0.64) | 20 | 10 | 10 | 0.59 | NA | NA | NA | No | No | 0.64 | ns |
| Individual Splindle Sigma power µV2/Hz | 1 | SMD, -0.25 (-0.89, 0.4) | 38 | 21 | 17 | 0.45 | NA | NA | NA | No | No | 0.64 | ns |
| Individual Splindle Duration sec | 1 | SMD, -0.24 (-0.88, 0.4) | 38 | 21 | 17 | 0.47 | NA | NA | NA | No | No | 0.65 | ns |
| Spindles frecuency Hz | 1 | SMD, -0.23 (-0.88, 0.41) | 40 | 15 | 25 | 0.48 | NA | NA | NA | No | No | 0.65 | ns |
| No of finger taps per 10 s, non-preferred hand, occasion 3 | 1 | SMD, 0.24 (-0.18, 0.65) | 92 | 37 | 55 | 0.27 | NA | NA | NA | No | No | 1.53 | ns |
| REM density | 9 | SMD, 0.24 (-0.11, 0.58) | 382 | 185 | 197 | 0.19 | 56% | -0.78, 1.25 | No | No | No | 1.53 | ns |
| Nonlinearity scores of EEG-C4 Stage 1/2 | 1 | SMD, 0.24 (-0.69, 1.16) | 18 | 9 | 9 | 0.62 | NA | NA | NA | No | No | 1.53 | ns |
| Awake time 2nd sleep cycle %NREM period | 1 | SMD, 0.22 (-0.38, 0.83) | 42 | 22 | 20 | 0.47 | NA | NA | NA | No | No | 1.50 | ns |
| Stage 1 % 1st night | 1 | SMD, 0.22 (-0.66, 1.1) | 20 | 10 | 10 | 0.62 | NA | NA | NA | No | No | 1.50 | ns |
| REM latency | 20 | SMD, -0.22 (-0.45, 0.01) | 490 | 238 | 252 | 0.056 | 30% | -0.87, 0.43 | No | No | No | 0.67 | ns |
| Stage 1 % averaged night 2+3 | 1 | SMD, 0.22 (-0.66, 1.1) | 20 | 10 | 10 | 0.62 | NA | NA | NA | No | No | 1.49 | ns |
| Intra-REM sleep periods Intermediate Sleep % of REM sleep duration | 1 | SMD, 0.22 (-0.66, 1.1) | 20 | 10 | 10 | 0.63 | NA | NA | NA | No | No | 1.48 | ns |
| Synkinesis | 2 | OR, 0.68 (0.11, 4.28) | 107 | 60 | 47 | 0.68 | 0% | NA | NA | No | No | 0.68 | ns |
| Tone increase R | 2 | OR, 0.68 (0.11, 4.26) | 107 | 60 | 47 | 0.68 | 0% | NA | NA | No | No | 0.68 | ns |
| Tone increase L | 2 | OR, 0.68 (0.11, 4.26) | 107 | 60 | 47 | 0.68 | 0% | NA | NA | No | No | 0.68 | ns |
| Plantar R | 2 | OR, 0.68 (0.11, 4.26) | 107 | 60 | 47 | 0.68 | 0% | NA | NA | No | No | 0.68 | ns |
| Plantar L | 2 | OR, 0.68 (0.11, 4.26) | 107 | 60 | 47 | 0.68 | 0% | NA | NA | No | No | 0.68 | ns |
| Glabellar tap | 2 | OR, 0.68 (0.11, 4.26) | 107 | 60 | 47 | 0.68 | 0% | NA | NA | No | No | 0.68 | ns |
| Suck reflex | 2 | OR, 0.68 (0.11, 4.26) | 107 | 60 | 47 | 0.68 | 0% | NA | NA | No | No | 0.68 | ns |
| Finger–nose R | 2 | OR, 0.68 (0.11, 4.26) | 107 | 60 | 47 | 0.68 | 0% | NA | NA | No | No | 0.68 | ns |
| Finger–nose L | 2 | OR, 0.68 (0.11, 4.26) | 107 | 60 | 47 | 0.68 | 0% | NA | NA | No | No | 0.68 | ns |
| REM % | 22 | SMD, -0.22 (-0.44, 0.01) | 747 | 364 | 383 | 0.056 | 47% | -0.98, 0.55 | No | No | No | 0.68 | ns |
| REM latency 1st night | 1 | SMD, -0.21 (-1.09, 0.67) | 20 | 10 | 10 | 0.64 | NA | NA | NA | No | No | 0.68 | ns |
| Stage 4 latency | 1 | SMD, -0.21 (-0.83, 0.41) | 40 | 20 | 20 | 0.51 | NA | NA | NA | No | No | 0.68 | ns |
| Slow wave sleep % 2nd night | 1 | SMD, -0.21 (-1.09, 0.67) | 20 | 10 | 10 | 0.64 | NA | NA | NA | No | No | 0.68 | ns |
| Finger-thumb opposition left | 1 | SMD, 0.21 (-0.1, 0.53) | 161 | 68 | 93 | 0.18 | NA | NA | NA | No | No | 1.47 | ns |
| No of finger taps per 10 s, preferred hand, occasion 3 | 1 | SMD, 0.2 (-0.22, 0.62) | 92 | 37 | 55 | 0.34 | NA | NA | NA | No | No | 1.44 | ns |
| REM time (second sleep cycle) | 1 | SMD, 0.2 (-0.41, 0.81) | 42 | 22 | 20 | 0.52 | NA | NA | NA | No | No | 1.43 | ns |
| Mean power frequency Hz, eyes open on yielding surface, medio-lateral | 1 | SMD, 0.2 (-0.55, 0.95) | 29 | 11 | 18 | 0.61 | NA | NA | NA | No | No | 1.43 | ns |
| Stage 4 % 1st night | 1 | SMD, 0.19 (-0.69, 1.07) | 20 | 10 | 10 | 0.67 | NA | NA | NA | No | No | 1.42 | ns |
| First REM density (2nd night) | 1 | SMD, -0.19 (-1.07, 0.69) | 20 | 10 | 10 | 0.68 | NA | NA | NA | No | No | 0.71 | ns |
| Spindle density | 1 | SMD, 0.19 (-0.65, 1.02) | 22 | 11 | 11 | 0.66 | NA | NA | NA | No | No | 1.40 | ns |
| Slow wave sleep % 3rd night | 1 | SMD, 0.18 (-0.7, 1.06) | 20 | 10 | 10 | 0.69 | NA | NA | NA | No | No | 1.39 | ns |
| REM activity (units) | 2 | SMD, -0.18 (-0.71, 0.34) | 59 | 28 | 31 | 0.49 | 0% | NA | NA | No | No | 0.72 | ns |
| REM density 3rd night | 1 | SMD, -0.18 (-1.06, 0.69) | 20 | 10 | 10 | 0.68 | NA | NA | NA | No | No | 0.72 | ns |
| Finger-thumb opposition right | 1 | SMD, 0.18 (-0.14, 0.49) | 161 | 68 | 93 | 0.27 | NA | NA | NA | No | No | 1.38 | ns |
| Position 4 sway in the medial-lateral direction cm | 1 | SMD, 0.18 (-0.29, 0.64) | 72 | 36 | 36 | 0.46 | NA | NA | NA | No | No | 1.38 | ns |
| Position 4 mean sway cm | 1 | SMD, 0.18 (-0.29, 0.64) | 72 | 36 | 36 | 0.46 | NA | NA | NA | No | No | 1.38 | ns |
| REM number | 3 | SMD, -0.17 (-0.58, 0.23) | 99 | 48 | 51 | 0.4 | 0% | -2.78, 2.43 | Yes | No | No | 0.73 | ns |
| The fast component CoP-CoM cm, eyes closed on firm surface, antero-posterior | 1 | SMD, 0.17 (-0.58, 0.92) | 29 | 11 | 18 | 0.66 | NA | NA | NA | No | No | 1.36 | ns |
| Chorea R | 2 | OR, 1.35 (0.13, 13.74) | 107 | 60 | 47 | 0.8 | 0% | NA | NA | No | No | 1.35 | ns |
| Chorea L | 2 | OR, 1.35 (0.13, 13.74) | 107 | 60 | 47 | 0.8 | 0% | NA | NA | No | No | 1.35 | ns |
| Relative spectral amplitude uV, beta1 | 1 | SMD, 0.16 (-0.56, 0.88) | 40 | 10 | 30 | 0.66 | NA | NA | NA | No | No | 1.34 | ns |
| First REM density (averaged night 2+3) | 1 | SMD, -0.16 (-1.03, 0.72) | 20 | 10 | 10 | 0.73 | NA | NA | NA | No | No | 0.75 | ns |
| Symbolic dynamic measures of EEG-C4 Stage 1/2 (SD of the word sequence) | 1 | SMD, -0.16 (-1.09, 0.76) | 18 | 9 | 9 | 0.73 | NA | NA | NA | No | No | 0.75 | ns |
| Fist-edge palm test left | 1 | SMD, 0.16 (-0.16, 0.47) | 161 | 68 | 93 | 0.33 | NA | NA | NA | No | No | 1.33 | ns |
| AIMS | 2 | OR, 1.33 (0.28, 6.46) | 107 | 60 | 47 | 0.72 | 45% | NA | NA | No | No | 1.33 | ns |
| REM density averaged night 2+3 | 1 | SMD, 0.16 (-0.72, 1.04) | 20 | 10 | 10 | 0.73 | NA | NA | NA | No | No | 1.33 | ns |
| Romberg | 2 | OR, 0.76 (0.16, 3.56) | 107 | 60 | 47 | 0.73 | 0% | NA | NA | No | No | 0.76 | ns |
| Tremor L | 2 | OR, 0.76 (0.16, 3.56) | 107 | 60 | 47 | 0.73 | 0% | NA | NA | No | No | 0.76 | ns |
| First REM duration | 4 | SMD, -0.15 (-0.5, 0.2) | 131 | 65 | 66 | 0.39 | 0% | -0.92, 0.61 | No | No | No | 0.76 | ns |
| Spindles sigma power µV2/Hz | 1 | SMD, 0.15 (-0.49, 0.79) | 40 | 15 | 25 | 0.65 | NA | NA | NA | No | No | 1.31 | ns |
| Stage 3 % 3rd night | 1 | SMD, 0.14 (-0.73, 1.02) | 20 | 10 | 10 | 0.75 | NA | NA | NA | No | No | 1.30 | ns |
| Relative abundance of Neisseria_sp._oral_taxon_014 in oropharynx | 1 | SMD, 0.15 (-0.55, 0.84) | 32 | 16 | 16 | 0.68 | NA | NA | NA | No | Yes | 1.30 | ns |
| Spindles | 1 | SMD, -0.15 (-0.47, 0.17) | 150 | 75 | 75 | 0.37 | NA | NA | NA | No | No | 0.77 | ns |
| REM density 1st night | 1 | SMD, -0.15 (-1.02, 0.73) | 20 | 10 | 10 | 0.75 | NA | NA | NA | No | No | 0.77 | ns |
| Stage 4 % 3rd night | 1 | SMD, 0.14 (-0.74, 1.01) | 20 | 10 | 10 | 0.76 | NA | NA | NA | No | No | 1.28 | ns |
| Mirror movements R | 2 | OR, 1.27 (0.22, 7.35) | 107 | 60 | 47 | 0.79 | 0% | NA | NA | No | No | 1.27 | ns |
| No of finger taps per 10 s, non-preferred hand, occasion 1 | 1 | SMD, -0.13 (-0.55, 0.29) | 92 | 37 | 55 | 0.54 | NA | NA | NA | No | No | 0.79 | ns |
| Mean power frequency Hz, eyes closed on firm surface, antero-posterior | 1 | SMD, -0.13 (-0.88, 0.62) | 29 | 11 | 18 | 0.74 | NA | NA | NA | No | No | 0.79 | ns |
| Cranial palsy R | 2 | OR, 0.81 (0.21, 3.19) | 107 | 60 | 47 | 0.77 | 0% | NA | NA | No | No | 0.81 | ns |
| Cranial palsy L | 2 | OR, 0.81 (0.21, 3.2) | 107 | 60 | 47 | 0.77 | 0% | NA | NA | No | No | 0.81 | ns |
| Tremor R | 2 | OR, 0.81 (0.21, 3.19) | 107 | 60 | 47 | 0.77 | 0% | NA | NA | No | No | 0.81 | ns |
| Finger–thumb opposition R | 2 | OR, 0.81 (0.2, 3.26) | 107 | 60 | 47 | 0.77 | 0% | NA | NA | No | No | 0.81 | ns |
| Individual Spindles Amplitude µV | 1 | SMD, -0.12 (-0.76, 0.52) | 38 | 21 | 17 | 0.72 | NA | NA | NA | No | No | 0.81 | ns |
| D-serine (Plasma) | 4 | SMD, -0.12 (-0.66, 0.42) | 419 | 272 | 147 | 0.67 | 85% | -2.57, 2.34 | No | No | Yes | 0.81 | ns |
| Diadochokinesia left | 1 | SMD, 0.12 (-0.2, 0.43) | 161 | 68 | 93 | 0.47 | NA | NA | NA | No | No | 1.23 | ns |
| Sleep spindles density nb/hour stage 2 | 1 | SMD, -0.11 (-0.99, 0.77) | 20 | 10 | 10 | 0.81 | NA | NA | NA | No | No | 0.82 | ns |
| Total finger ridge count (TFRC) | 14 | SMD, -0.11 (-0.28, 0.05) | 1919 | 964 | 955 | 0.19 | 63% | -0.67, 0.45 | No | Yes | No | 0.82 | ns |
| Stage 2 % | 24 | SMD, 0.11 (-0.2, 0.41) | 809 | 396 | 413 | 0.49 | 72% | -1.23, 1.44 | No | Yes | No | 1.21 | ns |
| REM latency 2nd night | 1 | SMD, -0.1 (-0.98, 0.78) | 20 | 10 | 10 | 0.82 | NA | NA | NA | No | No | 0.83 | ns |
| Stage 3 % 1st night | 1 | SMD, -0.1 (-0.98, 0.78) | 20 | 10 | 10 | 0.82 | NA | NA | NA | No | No | 0.83 | ns |
| Stage 4 % averaged night 2+3 | 1 | SMD, 0.1 (-0.78, 0.97) | 20 | 10 | 10 | 0.83 | NA | NA | NA | No | No | 1.19 | ns |
| Tandem walk | 2 | OR, 0.85 (0.24, 2.95) | 107 | 60 | 47 | 0.79 | 0% | NA | NA | No | No | 0.85 | ns |
| REM efficiency | 2 | SMD, -0.09 (-0.64, 0.46) | 51 | 25 | 26 | 0.76 | 0% | NA | NA | No | No | 0.85 | ns |
| Slow wave sleep % averaged night 2+3 | 1 | SMD, -0.09 (-0.96, 0.79) | 20 | 10 | 10 | 0.84 | NA | NA | NA | No | No | 0.85 | ns |
| Stage 2: 4th Quarter (S2q4) % | 1 | SMD, 0.09 (-0.64, 0.81) | 29 | 14 | 15 | 0.82 | NA | NA | NA | No | No | 1.17 | ns |
| Awake time % 3rd night | 1 | SMD, 0.09 (-0.79, 0.97) | 20 | 10 | 10 | 0.84 | NA | NA | NA | No | No | 1.17 | ns |
| Stage 1 % 2nd night | 1 | SMD, 0.08 (-0.79, 0.96) | 20 | 10 | 10 | 0.85 | NA | NA | NA | No | No | 1.16 | ns |
| REM number (1st night) | 1 | SMD, 0.08 (-0.8, 0.95) | 20 | 10 | 10 | 0.86 | NA | NA | NA | No | No | 1.15 | ns |
| Position 7 sway in the medial-lateral direction cm | 1 | SMD, 0.08 (-0.39, 0.54) | 72 | 36 | 36 | 0.75 | NA | NA | NA | No | No | 1.15 | ns |
| Hypereflexia R | 2 | OR, 0.87 (0.08, 9.86) | 107 | 60 | 47 | 0.91 | 0% | NA | NA | No | No | 0.87 | ns |
| Hypereflexia L | 2 | OR, 0.87 (0.08, 9.86) | 107 | 60 | 47 | 0.91 | 0% | NA | NA | No | No | 0.87 | ns |
| Stereognosis L | 2 | OR, 0.87 (0.08, 9.86) | 107 | 60 | 47 | 0.91 | 0% | NA | NA | No | No | 0.87 | ns |
| Lipopolysaccharide binding protein | 1 | SMD, -0.08 (-0.35, 0.2) | 219 | 141 | 78 | 0.58 | NA | NA | NA | No | No | 0.87 | ns |
| First REM activity (units) (age adjusted) | 1 | SMD, 0.07 (-0.78, 0.92) | 24 | 8 | 16 | 0.87 | NA | NA | NA | No | No | 1.14 | ns |
| Slow wave sleep % 1st night | 1 | SMD, 0.07 (-0.81, 0.95) | 20 | 10 | 10 | 0.87 | NA | NA | NA | No | No | 1.14 | ns |
| Stage 4 % 2nd night | 1 | SMD, 0.07 (-0.81, 0.95) | 20 | 10 | 10 | 0.88 | NA | NA | NA | No | No | 1.13 | ns |
| Initial Pyramidal | 1 | SMD, 0.06 (-0.37, 0.49) | 83 | 43 | 40 | 0.77 | NA | NA | NA | No | No | 1.12 | ns |
| REM % 2nd night | 1 | SMD, 0.06 (-0.81, 0.94) | 20 | 10 | 10 | 0.89 | NA | NA | NA | No | No | 1.12 | ns |
| REM latency (age adjusted) | 1 | SMD, 0.06 (-0.79, 0.91) | 24 | 8 | 16 | 0.88 | NA | NA | NA | No | No | 1.12 | ns |
| Individual Splindle Frequency Hz | 1 | SMD, -0.06 (-0.7, 0.58) | 38 | 21 | 17 | 0.85 | NA | NA | NA | No | No | 0.90 | ns |
| Position 4 sway in the anterior-posterior direction cm | 1 | SMD, 0.06 (-0.4, 0.52) | 72 | 36 | 36 | 0.8 | NA | NA | NA | No | No | 1.11 | ns |
| Hypokinesia | 1 | OR, 0.91 (0.23, 3.56) | 92 | 37 | 55 | 0.9 | NA | NA | NA | No | No | 0.91 | ns |
| Extinction | 2 | OR, 0.91 (0.33, 2.52) | 107 | 60 | 47 | 0.85 | 0% | NA | NA | No | No | 0.91 | ns |
| First REM density (3rd night) | 1 | SMD, -0.05 (-0.93, 0.83) | 20 | 10 | 10 | 0.91 | NA | NA | NA | No | No | 0.91 | ns |
| Diadochokinesia right | 1 | SMD, 0.05 (-0.27, 0.36) | 161 | 68 | 93 | 0.78 | NA | NA | NA | No | No | 1.09 | ns |
| First REM duration (averaged night 2+3) | 1 | SMD, 0.05 (-0.83, 0.92) | 20 | 10 | 10 | 0.92 | NA | NA | NA | No | No | 1.09 | ns |
| Number of rapid eye movements during REM sleep | 1 | SMD, 0.05 (-0.68, 0.78) | 29 | 14 | 15 | 0.89 | NA | NA | NA | No | No | 1.09 | ns |
| Graphaesthesia R | 2 | OR, 0.92 (0.34, 2.44) | 107 | 60 | 47 | 0.86 | 0% | NA | NA | No | No | 0.92 | ns |
| Awakenings number/hour | 1 | SMD, -0.05 (-0.69, 0.59) | 38 | 21 | 17 | 0.89 | NA | NA | NA | No | No | 0.92 | ns |
| Awake time % 1st night | 1 | SMD, -0.04 (-0.92, 0.83) | 20 | 10 | 10 | 0.92 | NA | NA | NA | No | No | 0.92 | ns |
| Relative spectral amplitude uV, delta | 1 | SMD, 0.04 (-0.67, 0.76) | 40 | 10 | 30 | 0.91 | NA | NA | NA | No | No | 1.08 | ns |
| Smooth pursuit eye movement | 1 | OR, 0.93 (0.27, 3.26) | 92 | 37 | 55 | 0.91 | NA | NA | NA | No | No | 0.93 | ns |
| Smooth pursuit | 2 | OR, 0.93 (0.37, 2.35) | 107 | 60 | 47 | 0.88 | 0% | NA | NA | No | No | 0.93 | ns |
| Targeting of Abnormal Kinetic Effects scale (TAKE) | 2 | OR, 0.94 (0.38, 2.34) | 107 | 60 | 47 | 0.89 | 0% | NA | NA | No | No | 0.94 | ns |
| Audiovisual integration | 2 | OR, 0.94 (0.38, 2.32) | 107 | 60 | 47 | 0.89 | 0% | NA | NA | No | No | 0.94 | ns |
| REM sleep min | 1 | SMD, 0.03 (-0.85, 0.91) | 20 | 10 | 10 | 0.95 | NA | NA | NA | No | No | 1.06 | ns |
| Alpha (8.0-12.0) | 2 | SMD, -0.03 (-0.83, 0.77) | 24 | 12 | 12 | 0.95 | 0% | NA | NA | No | No | 0.95 | ns |
| First REM duration (age adjusted) | 1 | SMD, -0.03 (-0.88, 0.82) | 24 | 8 | 16 | 0.95 | NA | NA | NA | No | No | 0.95 | ns |
| Stage 2 Incidence | 1 | SMD, 0.03 (-0.74, 0.8) | 26 | 13 | 13 | 0.95 | NA | NA | NA | No | No | 1.05 | ns |
| Dyskinesia | 5 | OR, 0.96 (0.55, 1.67) | 407 | 189 | 218 | 0.89 | 0% | 0.39, 2.35 | No | NA | NA | 0.96 | ns |
| Parkinsonism | 3 | OR, 0.98 (0.46, 2.05) | 234 | 84 | 150 | 0.95 | 0% | 0.01, 120.35 | NA | NA | NA | 0.98 | ns |
| Facial expression | 1 | OR, 0.99 (0.4, 2.46) | 92 | 37 | 55 | 0.98 | NA | NA | NA | No | No | 0.99 | ns |
| ATD angle | 6 | SMD, 0.01 (-0.36, 0.37) | 582 | 311 | 271 | 0.97 | 77% | -1.21, 1.23 | No | No | No | 1.01 | ns |
| AIMS facial and oral movements | 1 | SMD, 0 (-0.59, 0.59) | 44 | 22 | 22 | 1.0 | NA | NA | NA | No | No | 1.00 | ns |
| REM number (2nd night) | 1 | SMD, 0 (-0.88, 0.88) | 20 | 10 | 10 | 1.0 | NA | NA | NA | No | No | 1.00 | ns |
| Spectral Power during Stage 2 sleep µV2/Hz - Beta (15-30Hz) | 1 | SMD, 0 (-0.64, 0.64) | 40 | 15 | 25 | 1.0 | NA | NA | NA | No | No | 1.00 | ns |
| Nonlinearity scores of EEG-C4 REM | 1 | SMD, 0 (-0.92, 0.92) | 18 | 9 | 9 | 1.0 | NA | NA | NA | No | No | 1.00 | ns |
| Nonlinearity scores of EEG-F4 REM | 1 | SMD, 0 (-0.92, 0.92) | 18 | 9 | 9 | 1.0 | NA | NA | NA | No | No | 1.00 | ns |
| Largest Lyapunov exponent of EEG-C4 Stage 1/2 | 1 | SMD, 0 (-0.92, 0.92) | 18 | 9 | 9 | 1.0 | NA | NA | NA | No | No | 1.00 | ns |
| Largest Lyapunov exponent of EEG-F4 Stage 1/2 | 1 | SMD, 0 (-0.92, 0.92) | 18 | 9 | 9 | 1.0 | NA | NA | NA | No | No | 1.00 | ns |
| Largest Lyapunov exponent of EEG-F4 Stage 3/4 | 1 | SMD, 0 (-0.92, 0.92) | 18 | 9 | 9 | 1.0 | NA | NA | NA | No | No | 1.00 | ns |

AIMS - Abnormal Involuntary Movements scale, CI – confidence interval, CE – class of evidence, CoM – center of mass, CoP – center of pressure, CSF - cerebrospinal fluid, EEG - electroencephalogram, eOR – equivalent odds ratio, Egger – significant Egger test, ES – effect size, ESB – excess significance bias, k – number of studies for each factor, L – left, LS - largest study with significant effect, N – total number of participants, NA – not assessable, NES - Neurological Evaluation Scale, NREM - non-rapid eye movement, ns – not significant, NSS – neurological soft signs, OR – odds ratio, PI – prediction interval, R – right, REM – rapid eye movement, SD – standard deviation, SMD – standardized mean difference, WCST - Wisconsin Card Sorting Test.

**REFERENCES**

1. Ezeoke A, Mellor A, Buckley A, Miller B. A systematic, quantitative review of blood autoantibodies in schizophrenia. Schizophr Bull. 2013;150(1):245-251.

2. Wang D, Zhai J xia, Liu D wu. Serum folate levels in schizophrenia: a meta-analysis. Psychiatry Res. 2016;235:83-89.

3. Rodrigues-Amorim D, Rivera-Baltanás T, López M, Spuch C, Olivares JM, Agís-Balboa RC. Schizophrenia: a review of potential biomarkers. J Psychiatr Res. 2017;93:37-49.

4. Miller BJ, Buckley P, Seabolt W, Mellor A, Kirkpatrick B. Meta-analysis of cytokine alterations in schizophrenia: clinical status and antipsychotic effects. Biol Psychiatry. 2011;70(7):663-671.

5. Goldsmith DR, Rapaport MH, Miller BJ. A meta-analysis of blood cytokine network alterations in psychiatric patients: comparisons between schizophrenia, bipolar disorder and depression. Mol Psychiatry. 2016;21(12):1696-1709.

6. Wang AK, Miller BJ. Meta-analysis of cerebrospinal fluid cytokine and tryptophan catabolite alterations in psychiatric patients: comparisons between schizophrenia, bipolar disorder, and depression. Schizophr Bull. 2018;44(1):75-83.

7. Marcolin MA, Davis JM. Platelet monoamine oxidase in schizophrenia: a meta-analysis. Schizophr Res. 1992;7(3):249-267.

8. Karanikas E, Antoniadis D, Garyfallos GD. The role of cortisol in first episode of psychosis: a systematic review. Curr Psychiatry Rep. 2014;16(11):1-10.

9. Zorn J V., Schür RR, Boks MP, Kahn RS, Joëls M, Vinkers CH. Cortisol stress reactivity across psychiatric disorders: a systematic review and meta-analysis. Psychoneuroendocrinology. 2017;77:25-36.

10. Adamson J, Lally J, Gaughran F, Krivoy A, Allen L, Stubbs B. Correlates of vitamin D in psychotic disorders: a comprehensive systematic review. Psychiatry Res. 2017;249(November 2016):78-85.

11. Bartoli F, Lax A, Crocamo C, Clerici M, Carrà G. Plasma adiponectin levels in schizophrenia and role of second-generation antipsychotics: a meta-analysis. Psychoneuroendocrinology. 2015;56:179-189.

12. Chiam JTW, Dobson RJB, Kiddle SJ, Sattlecker M. Are blood-based protein biomarkers for Alzheimer’s disease also involved in other brain disorders? A systematic review. J Alzheimer’s Dis. 2014;43(1):303-314.

13. Grignon S, Marc J. Assessment of malondialdehyde levels in schizophrenia: a meta-analysis and some methodological considerations. Prog Neuropsychopharmacol Biol Psychiatry. 2007;31:365-369.

14. Girshkin L, Matheson SL, Shepherd AM, Green MJ. Morning cortisol levels in schizophrenia and bipolar disorder: a meta-analysis. Psychoneuroendocrinology. 2014;49(1):187-206.

15. González-Blanco L, Greenhalgh AMD, Garcia-Rizo C, Fernandez-Egea E, Miller BJ, Kirkpatrick B. Prolactin concentrations in antipsychotic-naïve patients with schizophrenia and related disorders: a meta-analysis. Schizophr Res. 2016;174(1-3):156-160.

16. Grain R, Lally J, Stubbs B, et al. Autoantibodies against voltage-gated potassium channel and glutamic acid decarboxylase in psychosis: a systematic review, meta-analysis, and case series. Psychiatry Clin Neurosci. 2017;71(10):678-689.

17. Guo J, Liu C, Wang Y, Feng B, Zhang X. Role of T helper lymphokines in the immune-inflammatory pathophysiology of schizophrenia: systematic review and meta-analysis. Nord J Psychiatry. 2015;69(5):364-372.

18. Mitchell RHB, Goldstein BI. Inflammation in children and adolescents with neuropsychiatric disorders: a systematic review. J Am Acad Child Adolesc Psychiatry. 2014;53(3):274-296.

19. Pillinger T, Beck K, Stubbs B, Howes OD. Cholesterol and triglyceride levels in first-episode psychosis: systematic review and meta-analysis. Br J Psychiatry. 2017;211(6):339-349.

20. Plitman E, Iwata Y, Caravaggio F, et al. Kynurenic acid in schizophrenia: a systematic review and meta-analysis. Schizophr Bull. 2017;43(4):764-777.

21. Polho GB, De-Paula VJ, Cardillo G, dos Santos B, Kerr DS. Leukocyte telomere length in patients with schizophrenia: a meta-analysis. Schizophr Res. 2015;165(2-3):195-200.

22. Schümberg K, Polyakova M, Steiner J, Schroeter ML. Serum s100b is related to illness duration and clinical symptoms in schizophrenia—a meta-regression analysis. Front Cell Neurosci. 2016;10(FEB):1-11.

23. Tuckwell HC, Koziol JA. A meta-analysis of homovanillic acid concentrations in schizophrenia. Int J Neurosci. 1993;73(1-2):109–114.

24. Davison J, Gorman AO, Brennan L, Cotter DR. A systematic review of metabolite biomarkers of schizophrenia. Schizophr Res. 2018;195:32-50.

25. Tuckwell HC, Koziol JA. On the concentration of 5-hydroxyindoleacetic acid in schizophrenia: a meta-analysis. Psychiatry Res. 1996;59(3):239-244.

26. Upthegrove R, Manzanares-Teson N, Barnes NM. Cytokine function in medication-naive first episode psychosis: a systematic review and meta-analysis. Schizophr Res. 2014;155(1-3):101-108.

27. Valipour G, Saneei P, Esmaillzadeh A. Serum vitamin D levels in relation to schizophrenia: a systematic review and meta-analysis of observational studies. J Clin Endocrinol Metab. 2014;99(10):3863-3872.

28. Wang DF, Cao B, Xu MY, et al. Meta-analyses of manganese superoxide dismutase activity, gene Ala-9Val polymorphism, and the risk of schizophrenia. Med (United States). 2015;94(36):1-7.

29. Rao S, Kota LN, Li Z, et al. Accelerated leukocyte telomere erosion in schizophrenia: evidence from the present study and a meta-analysis. J Psychiatr Res. 2016;79:50-56.

30. Qin XY, Wu HT, Cao C, Loh YP, Cheng Y. A meta-analysis of peripheral blood nerve growth factor levels in patients with schizophrenia. Mol Psychiatry. 2017;22(9):1306-1312.

31. Buoli M, Caldiroli A, Melter CC, Serati M, de Nijs J, Altamura AC. Biological aspects and candidate biomarkers for psychotic bipolar disorder: a systematic review. Psychiatry Clin Neurosci. 2016;70:227-244.

32. Miller BJ, Culpepper N, Rapaport MH. C-reactive protein levels in schizophrenia: a review and meta-analysis. Clin Schizophr Relat Psychoses. 2014;7(4):223-230.

33. Miller BJ, Gassama B, Sebastian D, Buckley P, Mellor A. Meta-analysis of lymphocytes in schizophrenia: clinical status and antipsychotic effects. Biol Psychiatry. 2013;73(10):993-999.

34. Flatow J, Buckley P, Miller BJ. Meta-analysis of oxidative stress in schizophrenia. Biol Psychiatry. 2013;74(6):400-409.

35. Rowbotham IM, Orsucci FF, Mansour MF, Chamberlain SR, Raja HY. Relevance of brain-derived neurotrophic factor levels in schizophrenia: a systematic review and meta-analysis. AIMS Neurosci. 2015;2(4):280-293.

36. Cui H, Jin Y, Wang J, Weng X, Li C. Serum brain-derived neurotrophic factor (BDNF) levels in schizophrenia: a systematic review. Shanghai Arch Psychiatry. 2012;24(5):250-261.

37. Green MJ, Matheson SL, Shepherd A, Weickert CS, Carr VJ. Brain-derived neurotrophic factor levels in schizophrenia: a systematic review with meta-analysis. Mol Psychiatry. 2011;16:960-972.

38. Toll A, Mané A. Brain-derived neurotrophic factor levels in first episode of psychosis: a systematic review. World J Psychiatry. 2015;5(1):154-159.

39. Nishi A, Numata S, Tajima A, et al. Meta-analyses of blood homocysteine levels for gender and genetic association studies of the MTHFR C677T polymorphism in schizophrenia. Schizophr Bull. 2014;40(5):1154-1163.

40. Thuné H, Recasens M, Uhlhaas PJ. The 40-Hz auditory steady-state response in patients with schizophrenia a meta-analysis. JAMA Psychiatry. 2016;73(11):1145-1153.

41. Ferreira-Santos F, Silveira C, Almeida PR, Palha A, Barbosa F, Marques-Teixeira J. The auditory P200 is both increased and reduced in schizophrenia? A meta-analytic dissociation of the effect for standard and target stimuli in the oddball task. Clin Neurophysiol. 2012;123(7):1300-1308.

42. Bramon E, Rabe-Hesketh S, Sham P, Murray RM, Frangou S. Meta-analysis of the P300 and P50 waveforms in schizophrenia. Schizophr Res. 2004;70:315-329.

43. Qiu Y qin, Tang Y xiang, Chan RCK, Sun X yang, He J. P300 aberration in first-episode schizophrenia patients: a meta-analysis. PLoS One. 2014;9(6):1-8.

44. Patterson J V, Hetrick WP, Boutros NN, et al. P50 sensory gating ratios in schizophrenics and controls: a review and data analysis. Psychiatry Res. 2008;158:226-247.

45. Cheng C hsiung, Chan P ying S, Liu C yih, Hsu S chieh. Auditory sensory gating in patients with bipolar disorders: a meta-analysis. J Affect Disord. 2016;203:199-203.

46. de Wilde OM, Bour LJ, Dingemans PM, Koelman JHTMTM, Linszen DH. A meta-analysis of P50 studies in patients with schizophrenia and relatives: differences in methodology between research groups. Schizophr Res. 2007;97(1-3):137-151.

47. Kremláček J, Kreegipuu K, Tales A, et al. Visual mismatch negativity (vMMN): a review and meta-analysis of studies in psychiatric and neurological disorders. Cortex. 2016;80:76-112.

48. Xiong Y, Li X, Zhao L, Wang C. Mismatch negativity in Han Chinese patients with schizophrenia: a meta-analysis. Shanghai Arch Psychiatry. 2017;29(5):259-267.

49. McCleery A, Lee J, Joshi A, Wynn JK, Hellemann GS, Green MF. Meta-analysis of face processing event-related potentials in schizophrenia. Biol Psychiatry. 2015;77(2):116-126.

50. Feuerriegel D, Churches O, Hofmann J, Keage HAD. The N170 and face perception in psychiatric and neurological disorders: a systematic review. Clin Neurophysiol. 2015;126(6):1141-1158.

51. Earls HA, Curran T, Mittal V. Deficits in early stages of face processing in schizophrenia: a systematic review of the P100 component. Schizophr Bull. 2016;42(2):519-527.

52. Rieger K, Hernandez LD, Baenninger A, Koenig T. 15 years of microstate research in schizophrenia - where are we? A meta-analysis. Front Psychiatry. 2016;7(FEB):1-7.

53. Avissar M, Xie S, Vail B, Lopez-Calderon J, Wang Y, Javitt DC. Meta-analysis of mismatch negativity to simple versus complex deviants in schizophrenia. Schizophr Res. 2018;191:25-34.

54. Haigh SM, Coffman BA, Salisbury DF. Mismatch negativity in first-episode schizophrenia: a meta-analysis. Clin EEG Neurosci. 2017;48(1):3-10.

55. Umbricht D, Krljes S. Mismatch negativity in schizophrenia: a meta-analysis. Schizophr Res. 2005;76:1-23.

56. Wang K, Cheung EFC, Gong Q yong, Chan RCK. Semantic processing disturbance in patients with schizophrenia: a meta-analysis of the N400 component. PLoS One. 2011;6(10).

57. Sayo A, Jennings RG, van Horn JD. Study factors influencing ventricular enlargement in schizophrenia: a 20 year follow-up meta-analysis. Neuroimage. 2012;59(1):154-167.

58. Brugger S, Davis JM, Leucht S, Stone JM. Proton Magnetic Resonance Spectroscopy and Illness Stage in Schizophrenia—A Systematic Review and Meta-Analysis. Biol Psychiatry. 2011;69(5):495-503.

59. Merritt K, Egerton A, Kempton MJ, Taylor MJ, McGuire PK. Nature of glutamate alterations in schizophrenia a meta-analysis of proton magnetic resonance spectroscopy studies. JAMA Psychiatry. 2016;73(7):665-674.

60. Laruelle M. Imaging dopamine transmission in schizophrenia. Quaterly J Nucl Med. 1998;42(3):211-221.

61. Olabi B, Ellison-Wright I, McIntosh AM, Wood SJ, Bullmore E, Lawrie SM. Are there progressive brain changes in schizophrenia? A meta-analysis of structural magnetic resonance imaging studies. Biol Psychiatry. 2011;70(1):88-96.

62. De Peri L, Crescini A, Deste G, Fusar-Poli P, Sacchetti E, Vita A. Brain structural abnormalities at the onset of schizophrenia and bipolar disorder: a meta-analysis of controlled magnetic resonance imaging studies. Curr Pharm Des. 2012;18(4):486-494.

63. Shepherd AM, Matheson SL, Laurens KR, Carr VJ, Green MJ. Systematic meta-analysis of insula volume in schizophrenia. Biol Psychiatry. 2012;72(9):775-784.

64. Fusar-Poli P, Meyer-Lindenberg A. Striatal presynaptic dopamine in schizophrenia, part I: meta-analysis of dopamine active transporter (DAT) density. Schizophr Bull. 2013;39(1):22-32.

65. Kempton MJ, Stahl D, Williams SCRR, DeLisi LE. Progressive lateral ventricular enlargement in schizophrenia: A meta-analysis of longitudinal MRI studies. Schizophr Res. 2010;120(1-3):54-62.

66. Kraguljac NV, Reid M, White D, et al. Neurometabolites in schizophrenia and bipolar disorder - a systematic review and meta-analysis. Psychiatry Res - Neuroimaging. 2012;203(2-3):111-125.

67. Shahab S, Stefanik L, Foussias G, Lai MC, Anderson KK, Voineskos AN. Sex and diffusion tensor imaging of white matter in schizophrenia: a systematic review plus meta-analysis of the corpus callosum. Schizophr Bull. 2018;44(1):203-221.

68. Geoffroy PA, Houenou J, Duhamel A, et al. The arcuate fasciculus in auditory-verbal hallucinations: a meta-analysis of diffusion-tensor-imaging studies. Schizophr Res. 2014;159(1):234-237.

69. Wright IC, Rabe-Hesketh S, Woodruff PWR, David AS, Murray RM, Bullmore ET. Meta-analysis of regional brain volumes in schizophrenia. Am J Psychiatry. 2000;157(1):16-25.

70. Modinos G, Costafreda SG, Van Tol MJ, McGuire PK, Aleman A, Allen P. Neuroanatomy of auditory verbal hallucinations in schizophrenia: a quantitative meta-analysis of voxel-based morphometry studies. Cortex. 2013;49(4):1046-1055.

71. Adriano F, Spoletini I, Caltagirone C, Spalletta G. Updated meta-analyses reveal thalamus volume reduction in patients with first-episode and chronic schizophrenia. Schizophr Res. 2010;123(1):1-14.

72. Marsman A, Van Den Heuvel MP, Klomp DWJ, Kahn RS, Luijten PR, Hulshoff Pol HE. Glutamate in schizophrenia: a focused review and meta-analysis of 1H-MRS studies. Schizophr Bull. 2013;39(1):120-129.

73. Trzesniak C, Kempton MJ, Busatto GF, et al. Adhesio interthalamica alterations in schizophrenia spectrum disorders: a systematic review and meta-analysis. Prog Neuro-Psychopharmacology Biol Psychiatry. 2011;35(4):877-886.

74. Hill K, Mann L, Laws KR, Stephenson CME, Nimmo-Smith, McKenna PJ. Hypofrontality in schizophrenia: a meta-analysis of functional imaging studies. Acta Psychiatr Scand. 2004;110(4):243-256.

75. Ward KE, Friedman L, Wise A, Schulz SC. Meta-analysis of brain and cranial size in schizophrenia. Schizophr Res. 1996;22(3):197-213.

76. Trzesniak C, Oliveira IR, Kempton MJ, et al. Are cavum septum pellucidum abnormalities more common in schizophrenia spectrum disorders? a systematic review and meta-analysis. Schizophr Res. 2011;125(1):1-12.

77. Arnone D, McIntosh AM, Tan GMY, Ebmeier KP. Meta-analysis of magnetic resonance imaging studies of the corpus callosum in schizophrenia. Schizophr Res. 2008;101(1-3):124-132.

78. Anticevic A, Van Snellenberg JX, Cohen RE, Repovs G, Dowd EC, Barch DM. Amygdala recruitment in schizophrenia in response to aversive emotional material: a meta-analysis of neuroimaging studies. Schizophr Bull. 2012;38(3):608-621.

79. Woods BT, Ward KE, Johnson EH. Meta-analysis of the time-course of brain volume reduction in schizophrenia: implications for pathogenesis and early treatment. Schizophr Res. 2005;73(2-3):221-228.

80. Vita A, De Peri L, Silenzi C, Dieci M. Brain morphology in first-episode schizophrenia: a meta-analysis of quantitative magnetic resonance imaging studies. Schizophr Res. 2006;82(1):75-88.

81. Steen RG, Hamer RM, Lieberman JA. Measurement of brain metabolites by 1H magnetic resonance spectroscopy in patients with schizophrenia: a systematic review and meta-analysis. Neuropsychopharmacology. 2005;30(11):1949-1962.

82. Radua J, Schmidt A, Borgwardt S, et al. Ventral striatal activation during reward processing in psychosis a neurofunctional meta-analysis. JAMA Psychiatry. 2015;72(12):1243-1251.

83. Fusar-Poli P, Perez J, Broome M, et al. Neurofunctional correlates of vulnerability to psychosis: a systematic review and meta-analysis. Neurosci Biobehav Rev. 2007;31(4):465-484.

84. Nordholm D, Krogh J, Mondelli V, Dazzan P, Pariante C, Nordentoft M. Pituitary gland volume in patients with schizophrenia, subjects at ultra high-risk of developing psychosis and healthy controls: a systematic review and meta-analysis. Psychoneuroendocrinology. 2013;38(11):2394-2404.

85. Walter A, Suenderhauf C, Harrisberger F, et al. Hippocampal volume in subjects at clinical high-risk for psychosis: a systematic review and meta-analysis. Neurosci Biobehav Rev. 2016;71:680-690.

86. Van Snellenberg JX, Torres IJ, Thornton AE. Functional neuroimaging of working memory in schizophrenia: task performance as a moderating variable. Neuropsychology. 2006;20(5):497-510.

87. Schür RR, Draisma LWR, Wijnen JP, et al. Brain GABA levels across psychiatric disorders: a systematic literature review and meta-analysis of 1H-MRS studies. Hum Brain Mapp. 2016;37(9):3337-3352.

88. Kambeitz J, Abi-Dargham A, Kapur S, Howes OD. Alterations in cortical and extrastriatal subcortical dopamine function in schizophrenia: systematic review and meta-analysis of imaging studies. Br J Psychiatry. 2014;204(6):420-429.

89. Vita A, De Peri L, Deste G, Barlati S, Sacchetti E. The effect of antipsychotic treatment on cortical gray matter changes in schizophrenia: does the class matter? A meta-analysis and meta-regression of longitudinal magnetic resonance imaging studies. Biol Psychiatry. 2015;78(6):403-412.

90. Zhuo C, Liu M, Wang L, Tian H, Tang J. Diffusion tensor MR imaging evaluation of callosal abnormalities in schizophrenia: a meta-analysis. PLoS One. 2016;11(8):1-12.

91. Fusar-Poli P, Meyer-Lindenberg A. Striatal presynaptic dopamine in schizophrenia, part II: meta-analysis of [18F/11C]-DOPA PET studies. Schizophr Bull. 2013;39(1):33-42.

92. Fusar-Poli P, Smieskova R, Kempton MJ, Ho BC, Andreasen NC, Borgwardt S. Progressive brain changes in schizophrenia related to antipsychotic treatment? A meta-analysis of longitudinal MRI studies. Neurosci Biobehav Rev. 2013;37(8):1680-1691.

93. Davidson LL, Heinrichs RW. Quantification of frontal and temporal lobe brain-imaging findings in schizophrenia: a meta-analysis. Psychiatry Res - Neuroimaging. 2003;122(2):69-87.

94. Fraguas D, Díaz-Caneja CM, Pina-Camacho L, Janssen J, Arango C. Progressive brain changes in children and adolescents with early-onset psychosis: a meta-analysis of longitudinal MRI studies. Schizophr Res. 2016;173(3):132-139.

95. Baiano M, David A, Versace A, Churchill R, Balestrieri M, Brambilla P. Anterior cingulate volumes in schizophrenia: a systematic review and a meta-analysis of MRI studies. Schizophr Res. 2007;93(1-3):1-12.

96. Sommer I, Aleman A, Ramsey N, Bouma A, Kahn R. Handedness, language lateralisation and anatomical asymmetry in schizophrenia: meta-analysis. Br J Psychiatry. 2001;178(APR.):344-351.

97. Haijma S V., Van Haren N, Cahn W, Koolschijn PCMP, Hulshoff Pol HE, Kahn RS. Brain volumes in schizophrenia: a meta-analysis in over 18 000 subjects. Schizophr Bull. 2013;39(5):1129-1138.

98. Catts VS, Lai YL, Weickert CS, Weickert TW, Catts S V. A quantitative review of the postmortem evidence for decreased cortical N-methyl-d-aspartate receptor expression levels in schizophrenia: how can we link molecular abnormalities to mismatch negativity deficits? Biol Psychol. 2016;116:57-67.

99. Harrison PJ, Freemantle N, Geddes JR. Meta-analysis of brain weight in schizophrenia. Schizophr Res. 2003;64(1):25-34.

100. Trépanier MO, Hopperton KE, Mizrahi R, Mechawar N, Bazinet RP. Postmortem evidence of cerebral in flammation in schizophrenia: a systematic review. Mol Psychiatry. 2016;21:1009-1026.

101. Chung JK, Nakajima S, Plitman E, et al. Beta-amyloid burden is not associated with cognitive impairment in schizophrenia: a systematic review. Am J Geriatr Psychiatry. 2016;24(10):923-939.

102. Bachmann S, Degen C, Geider FJ, Schröder J. Neurological soft signs in the clinical course of schizophrenia: results of a meta-analysis. Front Psychiatry. 2014;5(DEC):14-18.

103. Cho SE, Na KS, Cho SJ, Kang SG. Low D-serine levels in schizophrenia: a systematic review and meta-analysis. Neurosci Lett. 2016;634:42-51.

104. Clamor A, Lincoln TM, Thayer JF, Koenig J. Resting vagal activity in schizophrenia: meta-analysis of heart rate variability as a potential endophenotype. Br J Psychiatry. 2016;208:9-16.

105. Davies G, Haddock G, Yung AR, Mulligan LD, Kyle SD. A systematic review of the nature and correlates of sleep disturbance in early psychosis. Sleep Med Rev. 2017;31:25-38.

106. Fujino H, Imura O. Postural sway and clinical characteristics in patients with psychotic disorders: a review. Psychiatr Q. 2015;86(4):603-614.

107. Golembo-Smith S, Walder DJ, Daly MP, et al. The presentation of dermatoglyphic abnormalities in schizophrenia: a meta-analytic review. Schizophr Res. 2012;142(1-3):1-11.

108. Koning JPF, Tenback DE, Van Os J, Aleman A, Kahn RS, Van Harten PN. Dyskinesia and parkinsonism in antipsychotic-naive patients with schizophrenia, first-degree relatives and healthy controls: a meta-analysis. Schizophr Bull. 2010;36(4):723-731.

109. Xu T, Chan RCK, Compton MT. Minor physical anomalies in patients with schizophrenia, unaffected first-degree relatives, and healthy controls: a meta-analysis. PLoS One. 2011;6(9):2-7.

110. Bernard P, Romain AJ, Vancampfort D, Baillot A, Esseul E, Ninot G. Six minutes walk test for individuals with schizophrenia. Disabil Rehabil. 2015;37(11):921-927.
